# Golgi-retained TNFR1 variants induce stress-driven inflammation in a subset of systemic juvenile idiopathic arthritis

**DOI:** 10.1172/JCI196975

**Published:** 2026-11-16

**Authors:** Anthony C. Cruz, Krisztian Csomos, Hiroto Nakano, Boglarka Ujhazi, Paul M. Panipinto, Sophia R. Chou, Marissa Krantz, Emily D. Rosenbaum, Mariana Correia Marques, Christopher Felicelli, Tian-Min Fu, Dirk Foell, Rae S.M. Yeung, Pamela F. Weiss, Faiza Naz, Ly-Lan Bergeron, Michelle Millwood, Carol Lake, Keisuke Nagao, Paul Schaughency, Vicky Chen, Justin Lack, Emely L. Verweyen, Grant Schulert, Kang Yu, INCHARGE Consortium, Stefania Dell’Orso, Hao Wu, Zuoming Deng, Davide Randazzo, Michael J. Ombrello

**Affiliations:** 1 Translational Genetics and Genomics Section, National Institute of Arthritis and Musculoskeletal and Skin Diseases (NIAMS), National Institutes of Health (NIH), Bethesda, Maryland, USA.; 2 Department of Pathology, University of Massachusetts Chan Medical School, Worcester, Massachusetts USA.; 3 Department of Pediatric Rheumatology and Immunology, University Hospital Muenster, Muenster, Germany.; 4 Departments of Pediatrics and Immunology, University of Toronto, Toronto, Ontario, Canada.; 5 University of Pennsylvania and Children’s Hospital of Philadelphia, Philadelphia, Pennsylvania, USA.; 6 Genomic Technology Section, Office of Science and Technology, NIAMS, NIH, Bethesda, Maryland, USA.; 7 Office of the Clinical Director, NIAMS, NIH, Bethesda, Maryland, USA.; 8 Cutaneous Leukocyte Biology Section, Dermatology Branch, NIAMS, NIH, Bethesda, Maryland, USA.; 9 Department of Dermatology, Keio University School of Medicine, Tokyo, Japan.; 10 Integrated Data Sciences Section, Research Technologies Branch, National Institute of Allergy and Infectious Diseases, NIH, Bethesda, Maryland, USA.; 11 Cincinnati Children’s Hospital Medical Center, Cincinnati, Ohio, USA.; 12 Lymphocyte Cell Biology Section, Molecular Immunology and Inflammation Branch, NIAMS, NIH, Bethesda, Maryland, USA.; 13 See Appendix A in Supplemental Materials for membership details.; 14 Department of Biological Chemistry and Molecular Pharmacology, Harvard Medical School, Boston, Massachusetts, USA.; 15 Biodata Mining and Discover Section, NIAMS, NIH, Bethesda, Maryland, USA.; 16 Light Imaging Section, NIAMS, NIH, Bethesda, Maryland, USA.

**Keywords:** Genetics, Immunology, Inflammation, Cell stress, Innate immunity, Monocytes

## Abstract

Systemic juvenile idiopathic arthritis (sJIA) is a complex inflammatory disease characterized by recurrent fevers, arthritis, and systemic hyperinflammation; however, its underlying etiology remains poorly understood. Tumor necrosis factor receptor 1 (TNFR1, *TNFRSF1A*), the prototypical TNF superfamily receptor, signals through its ligand (tumor necrosis factor, TNF) to activate NF-κB–dependent proinflammatory pathways while also promoting cell death. Through family-based and targeted sequencing, we identified multiple rare *TNFRSF1A* variants, including a de novo truncating variant in a patient with monophasic sJIA. Integrating single cell RNA-seq with in vitro and ex vivo functional assays, we uncovered a patient-specific, unique monocyte population marked by high inflammatory cytokine production. These cells display impaired endosomal and Golgi trafficking, dysregulated autophagy, and increased markers of cellular stress and immune activation. Confocal microscopy revealed aberrant TNFR1 retention within the Golgi apparatus. These monocytes were refractory to LPS-induced cell death, maintained a M1-like inflammatory phenotype and produced proinflammatory cytokines even at steady state. Despite conferring a loss of function in TNFR1 signaling, these variants paradoxically drive a hyperinflammatory phenotype. We propose that this results from combined effects of reduced TNFR1 signaling and cellular stress induced by retention of the mutant receptor in the Golgi.

## Abstract 2

Novel variants of TNFR1 induce an inflammatory phenotype caused by cellular stress through dysfunction of multiple cellular trafficking pathways.

## Graphical abstract

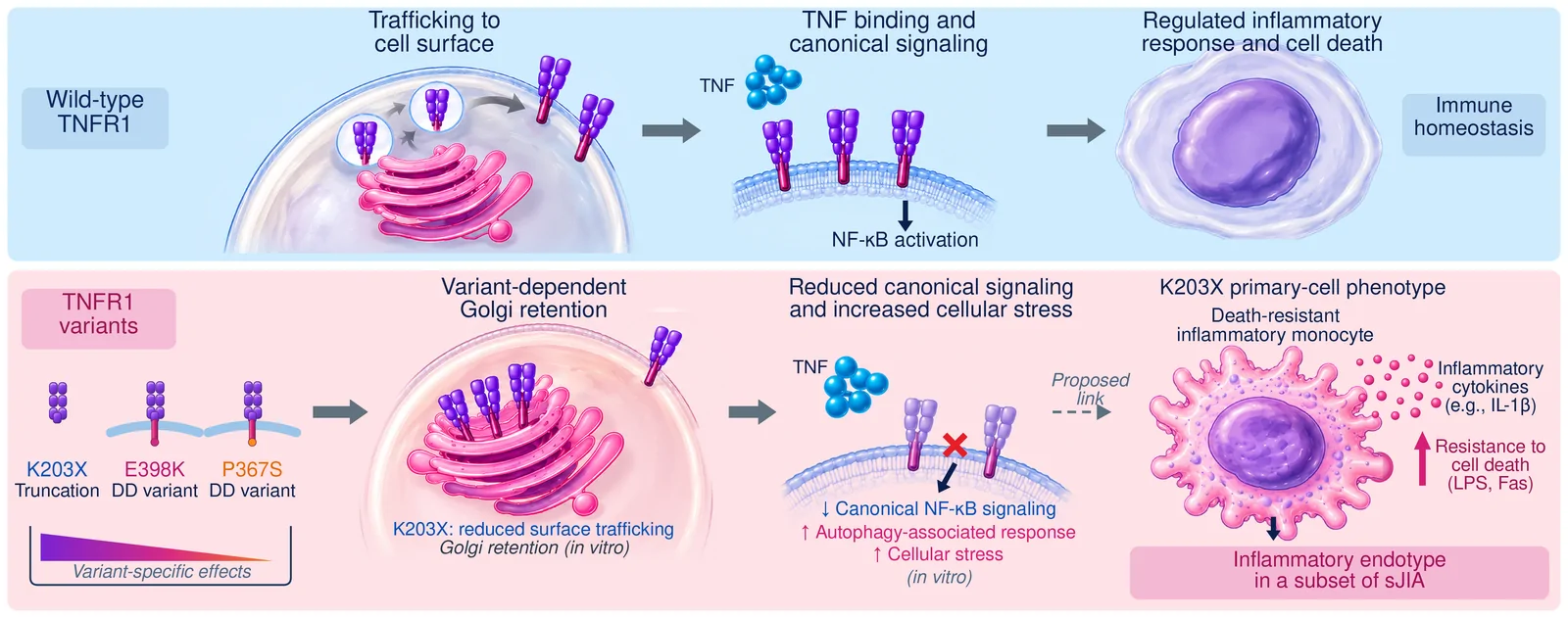

## Introduction

Still’s disease, also known as systemic juvenile idiopathic arthritis (sJIA) in children and adult-onset Still’s disease (AOSD) in adults, is a rare inflammatory disease characterized by recurrent fevers, chronic arthritis, a salmon pink skin evanescent rash, lymphadenopathy, hepatosplenomegaly, and serositis (1–3). People with Still’s disease can suffer life-threatening complications, including macrophage activation syndrome (1, 4), pericarditis, and, in recent years, interstitial lung disease (5, 6). It is a genetically complex disease involving genetic and environmental factors (1, 7), and while some of the clinical features of Still’s disease align with autoinflammatory diseases such as NOMID and TRAPS (8), variants in the genes that cause these monogenic diseases have not been found to contribute to sJIA (9). There is strong association between sJIA and the MHC class-II locus (10–13), and variants in the interleukin (IL)-1 receptor antagonist (*IL1RN)* and genes that cause hemophagocytic lymphohistiocytosis (HLH) genes also associate with sJIA (9, 14). Despite a lack of genetic association, IL-1 family cytokines themselves are also implicated in sJIA (9, 15–17).

TNFR1 is a ubiquitously expressed, prototypical member of the TNF Receptor Superfamily (TNFRSF). It has pleiotropic signaling capabilities, and, upon engagement by TNF, adaptor molecules are recruited to the death domain (DD) of TNFR1 to form signaling complexes that propagate downstream signaling (18–20). TNFR1 serum levels are a correlative biomarker for rheumatoid arthritis (21, 22) and are generally coupled to other periodic fever syndromes. TNFR1/TNFR2 ratios are elevated in patients with sJIA in both active and inactive disease (23), and the TNFR2/TNFR1 ratio is a proposed indicator of sJIA MAS development (24). However, the extent of TNFR1 involvement in sJIA remains unclear. Patients with sJIA often respond poorly to anti-TNF therapy, despite elevated TNF levels and strong NF-κB involvement, while therapies targeting IL-1 or IL-6 are more commonly effective (25). Chronic stimulation of TNFR1 typically promotes cell death, but in certain settings the TNF-TNFR1 signaling axis can induce a hyperinflammatory state, pushing cells towards a phenotype similar to cellular senescence (26–29).

Here, we describe novel variants of *TNFRSF1A* located in the DD of the receptor, identified in a cohort of patients with sJIA. Among these was a de novo variant of TNFR1 that encodes a truncated protein lacking the transmembrane and DD. These variants show variant-dependent impairment of canonical TNFR1 function, including reduced TNF-induced NF-κB activation and cell death. The patient harboring the truncated variant showed a proinflammatory transcriptional and cytokine profile, together with a distinct M1-like monocyte population that was resistant to cell death. In vitro and primary-cell analyses of K203X further implicated TNFR1 Golgi retention, cellular stress, and autophagy-associated dysregulation. We propose that these TNFR1 variants promote persistence of proinflammatory myeloid cells that propagate a hyperresponsive immune state, driven, in part, by TNFR1 Golgi retention, cellular stress, and autophagy-associated dysregulation.

## Results

### Ultra-rare variants of TNFRSF1A involving the DD in children with sJIA.

A female with early-onset sJIA fulfilling ILAR criteria (30) was evaluated in the Still’s disease clinic at the NIH Clinical Center. With onset at 3 years of age, her presentation was characterized by persistent fevers, rash, arthritis, anemia, leukocytosis with neutrophilia, thrombocytosis, hyperferritinemia (3,791 ng/ml), elevated erythrocyte sedimentation rate (106 mm/hr), and elevated C-reactive protein (13.9 mg/dl; Supplemental Table 1; supplemental material available online with this article; https://doi.org/10.1172/JCI196975DS1). Following a negative evaluation for infectious or autoimmune etiologies, she was treated with glucocorticoids and subsequently canakinumab, with rapid resolution of inflammatory features after her first dose, return to her previous healthy baseline, and discontinuation of steroids 6 weeks later. At study enrollment, after 3 doses of canakinumab, she was approaching clinical remission. Canakinumab was discontinued after 16 months of therapy, and she has remained in untreated remission since that time. At her second study visit, untreated remission had been present for 31 months. Despite this, IL-18 remained modestly elevated (658 pg/ml), and she was profoundly monocytopenic at both study visits (Figure 1A).

Trio exome sequencing revealed a de novo nonsense variant of *TNFRSF1A* encoding a truncated form of TNFR1 (K203X) lacking both the transmembrane and intracellular DDs (Figure 1B). K203X had a high CADD damage prediction score of 34, was predicted to result in loss of function (pLOF), and was absent among 590,031 individuals of non-Finnish European ancestry (NFE) in gnomAD (Figure 1C). To further explore rare *TNFRSF1A* variation in sJIA, we performed targeted sequencing in 525 children from the INCHARGE sJIA cohort (31). Although no second K203X carrier was identified, we found 2 additional ultra-rare missense *TNFRSF1A* variants, P367S and E398K, each carried by one individual (Figure 1, B–D). Like K203X, both variants involved the TNFR1 DD (Figure 1D) were ultra rare in gnomAD NFE (variant frequencies 1.3 × 10^–7^ and 4.2 × 10^–7^, respectively), and had high CADD scores (23 and 27, respectively; Figure 1C). Clinical features of these participants are shown in Supplemental Table 1.

Molecular modeling showed that both missense variants involved solvent-exposed residues situated on different faces of the DD (Figure 1D). E398 was predicted to interact with K343 in the DD of an adjacent TNFR1 molecule, suggesting that E398K could disrupt DD-dependent TNFR1 oligomerization (Figure 1E). P367 was predicted to interact with Q274 of the adaptor protein TRADD, suggesting that P367S could impair DD-dependent TRADD recruitment (Figure 1F). K203X, lacking both the transmembrane domain and DD, would be incapable of DD-dependent oligomerization or TRADD recruitment.

### TNFRSF1A variants found in sJIA produce defective DD-mediated interactions and reduce recruitment of proximal signaling components.

Given the predicted detrimental effects of these variants on DD-dependent oligomerization, we used fluorescent resonance energy transfer (FRET) to compare interactions of WT and variant TNFR1 in HEK293T cells. FRET between WT receptors showed a strong signal, whereas heterotypic interactions between WT TNFR1 and either E398K or K203X were severely reduced, similar to a membrane-bound negative control lacking the DD (ΔDD; Figure 2A). In contrast, P367S interacted robustly with WT TNFR1 (Figure 2A). A similar pattern was observed in homotypic interactions between paired variant receptors (Supplemental Figure 1, A and B). We also examined DD-dependent TRADD recruitment by FRET, which showed that K203X behaved similarly to the ΔDD negative control, whereas E398K and, to a lesser degree, P367S had reduced interaction with the TRADD DD relative to WT TNFR1 (Figure 2B).

TNFR1 engagement by TNF induces sequential recruitment of TRADD, TRAF2, and RIP as part of the proximal signaling complex, termed Complex I (19). To determine whether Complex I assembly was altered by variant TNFR1 proteins, we immunoprecipitated overexpressed WT or variant TNFR1 following TNF stimulation and assessed recruitment of Complex I components by Western blotting. WT TNFR1 showed spontaneous interaction with TRADD, TRAF2, and RIP that was enhanced by TNF stimulation. In contrast, each variant showed reduced recruitment of these binding partners under both steady-state and TNF-stimulated conditions (Supplemental Figure 1C-E). Complex I recruitment was most severely reduced by E398K and K203X and more modestly reduced by P367S. Together, these data show variant-dependent impairment of DD-mediated receptor interactions and proximal signaling complex formation.

### DD variants of TNFRSF1A lead to defective activation of NF-κB signaling and defective cell death.

TNFR1 can produce distinct signaling outcomes depending on signal strength, receptor localization, and duration of TNF stimulation (32). To assess signaling capacity, we first measured NF-κB activation by luciferase. Each variant significantly reduced spontaneous NF-κB activation relative to WT TNFR1, with K203X and E398K causing greater defects than P367S (Figure 2C). Because the luciferase assay does not fully model TNF-induced signaling, we also used Jurkat NF-κB reporter cells (33). In the presence of TNF, K203X and E398K showed reduced NF-κB activation relative to TNF-stimulated WT TNFR1, whereas P367S showed activity comparable with the WT receptor (Figure 2D). Collectively, these data show that TNFR1 variants impair NF-κB signaling in a variant-dependent manner at a steady state and after TNF stimulation.

We next determined whether impaired TNFR1 signaling altered susceptibility to cell death. Cells transfected with E398K or K203X TNFR1 showed significant decreases in spontaneous cell death. P367S also showed decreased cell death, although this decrease was not significant and remained closer to WT transfectants (Figure 2E). This pattern was recapitulated in another TNFR1-expressing cell line, where both K203X and E398K showed significantly decreased cell death, while P367S showed a smaller effect (Figure 2F). Together these data indicate that TNFR1-mediated cell death is reduced by these variants, with the strongest effects observed for K203X and E398K.

To directly assess TNFR1 signaling in primary patient cells, PBMCs from the K203X patient and healthy controls were stimulated with TNF ex vivo and analyzed by intracellular flow cytometry. K203X monocytes showed reduced TNF-induced phospho-p65 compared with healthy controls, both by frequency of positive cells and phosphorylation signal (Figure 2G). TNF-induced IL-1β and IL-8 responses were also diminished in K203X monocytes, both in percentage of cytokine-positive cells and magnitude of cytokine signal (Figure 2H and Supplemental Figure 2). Together, these findings demonstrate that canonical TNF-induced NF-κB signaling is attenuated in primary cells from the K203X patient.

### TNFR1 DD variants exhibit Golgi retention, expansion, and fragmentation in vitro and ex vivo.

Having established that the TNFR1 DD variants impair receptor interactions and proximal signaling, we examined receptor localization. Cells expressing K203X showed markedly reduced surface staining despite robust expression, whereas the other variants localized to the cell surface (Figure 3A). Super-resolution confocal imaging showed increased Golgi localization for all 3 variants relative to WT TNFR1, although the magnitude varied substantially. WT, P367S, and E398K TNFR1 formed puncta on the cell surface and within the cell, whereas K203X showed minimal membrane localization and preferential localization to the Golgi. E398K and P367S showed intermediate and modest Golgi accumulation, respectively (Figure 3B). This contrasted with the TRAPS-causing variant T50M, which localized predominantly to the ER (Supplemental Figure 3). Cells expressing each DD variant also displayed significantly higher GM130^+^ Golgi area than cells expressing WT TNFR1, consistent with altered Golgi morphology (Figure 3C).

We next assessed surface expression and localization of TNFR1 in PBMCs from the K203X patient. Surface TNFR1 remained detectable but was reduced compared with age-matched healthy controls in both percentage of positive cells and surface receptor expression (Figure 4A). Consistent with overexpression studies, super-resolution confocal imaging of primary K203X PBMCs showed enhanced TNFR1 localization in the Golgi (Figure 4, B and C), and K203X PBMCs displayed increased Golgi area compared with age-matched controls (Figure 4D). Together, these data demonstrate reduced surface trafficking and preferential Golgi retention of K203X TNFR1 in vitro and ex vivo.

Given that K203X TNFR1 is retained intracellularly, we hypothesized that it would contribute less to the circulating soluble TNFR1 (sTNFR1) pool. Serum sTNFR1 was elevated overall in patients with sJIA compared with age-matched healthy controls; however, both K203X samples had the lowest sTNFR1 levels across the entire sJIA cohort (Figure 4E). Collectively, these data suggest that K203X TNFR1 is preferentially sequestered within the Golgi, limiting its contribution to circulating sTNFR1 and coinciding with altered Golgi morphology in K203X-expressing cells.

### Peripheral blood single-cell transcriptomics uncovers monocyte-dominant NF-κB–associated inflammatory reprogramming in cells harboring the K203X variant of TNFR1.

To investigate the immune consequences of K203X, we performed PBMC single-cell RNA-seq (scRNA-seq) from the K203X patient. The participants carrying P367S and E398K were unavailable for follow-up biospecimen collection. Because the K203X patient was sampled during inactive disease, we integrated her data with PBMC scRNA-seq data from 5 inactive patients with sJIA and 5 children who were healthy controls (34) (Supplemental Figure 4). The K203X sample was collected at the second visit, when the patient was not receiving therapy, and was analyzed in 2 technical replicates. The inactive sJIA comparators were receiving therapy at sample collection (Supplemental Table 2). This design minimized confounding related to active disease while preserving the treatment-status distinction between the untreated K203X patient and the inactive sJIA cohort.

Unsupervised clustering identified the major PBMC leukocyte subsets, including monocytes, conventional dendritic cells, NK cells, CD4^+^ and CD8^+^ T cells, B cells, plasmablasts/plasma cells, plasmacytoid dendritic cells, minor T cell subsets (double-negative T cells, γ-δ T cells, and mucosal-associated invariant T cells), innate lymphocytes, and hematopoietic stem and progenitor cells (Figure 5A). Main subset abundance analysis revealed a significant decrease in the monocyte compartment of the K203X patient compared with healthy controls and other patients with sJIA (Figure 5B), consistent with the complete blood count (Supplemental Figure 4A). TNFR1 expression varied across leukocyte subsets, with highest expression in monocytes and lowest expression in B cells (Figure 5, C and D). The K203X patient showed reduced *TNFRSF1A* transcript levels across leukocyte subsets, with significant reductions in monocytes, NK cells, CD4^+^ T cells, and B cells (Figure 5D), while *TNFRSF1B* expression remained largely unchanged (Supplemental Figure 4, C–E).

Monocytes from K203X showed the strongest differentially expressed gene (DEG) signature, dominated by repressed genes, whereas monocytes from other patients with inactive sJIA displayed much less differential expression relative to healthy controls (Supplemental Figure 4F). TNFR pathway analysis revealed a discordant pattern in K203X monocytes, with reduced expression of *TNFRSF1A* and several upstream/proximal pathway components, including adaptors, signal transducers, and MAPK/PI3K-associated components, but increased expression of downstream inflammatory effector genes, including *TNF*, *IL1B*, *IL6*, *CXCL2*, *CXCL3*, *CCL20*, *SOCS3*, and *PTGS2* (Supplemental Figure 4, G and H). Targeted NF-κB pathway analysis similarly showed repression of the IκB kinase (IKK) complex with upregulation of canonical NF-κB target genes, including antiapoptotic genes, adhesion and immune-regulatory genes, and inflammatory cytokines and chemokines (Figure 5E). This pattern suggested activation of TNFR/ NF-κB–associated effector genes, despite reduced proximal TNFR pathway expression, consistent with inflammatory signaling that was not explained by intact canonical TNFR1 signaling alone. Thus, the transcriptomic data raised the possibility that the inflammatory NF-κB–associated signature in patient cells reflected secondary or parallel stress-associated pathways rather than acute ligand-driven TNFR1 signaling.

Analysis of inflammatory cytokine expression across major PBMC subsets showed that monocytes were the predominant source of *IL1B*, *TNF*, *IL6*, *IL8*, *IL18*, *CCL3*, and *CCL4* in the K203X patient (Figure 5F). Substantial expression of *IL1B*, *IL8*, *CCL3*, and *CCL4* was also observed in cDCs, while NK cells showed elevated *CCL3* and *CCL4* expression relative to healthy controls and other patients with sJIA. Total PBMC analysis supported a broader proinflammatory state that appeared to originate predominantly in monocytes. In serum, however, only IL-1β was highly elevated in the K203X patient compared with healthy controls, while other cytokines remained near levels observed in other patients with sJIA, suggesting a prominent role for IL-1β in this patient’s inflammatory phenotype (Figure 5G). Thus, circulating protein measurements only partially mirrored the cellular transcriptional inflammatory signature. Together, these findings show that K203X is associated with monocyte-dominant NF-κB–associated inflammatory cytokine expression despite reduced *TNFRSF1A* expression and monocyte abundance.

### K203X variant induces a monocyte-intrinsic cascade of vesicular dysfunction and cellular stress response.

The NF-κB–associated inflammatory program in K203X PBMCs contrasted with the attenuated canonical TNFR1 signaling observed in vitro and in primary TNF-stimulation assays (Figure 2). Because this discrepancy was most evident in monocytes, and because monocytes are central to sJIA pathogenesis, we subclustered the monocyte compartment and identified 5 clusters (Figure 6A). C1 corresponded to classical monocytes and C2 to nonclassical monocytes, while C3–C5 were defined by distinct marker genes, including *ADAMTS2* and *IL1R1* (C3), *EGR1*, *IFI44L*, and *SIGLEC1* (C4), and *IL6*, *CXCL3*, and other chemokines (C5), reflecting classical monocyte activation and inflammatory conditions (Figure 6B). C3 and C4 were enriched in the sJIA1–5 cohort, whereas C5 was unique to the K203X patient (Figure 6C). Cluster abundance analysis showed preserved monocyte subset distributions in healthy controls and 3 patients with sJIA, while C3, C4, and C5 were specifically enriched in sJIA-4, sJIA-5, and sJIA-K203X, respectively (Figure 6D). The K203X-specific C5 cluster expressed the highest levels of inflammatory cytokines, including *TNF*, *IL1B*, *IL6*, *IL8*, and *IL18* (Figure 6E), had the largest DEG burden (Figure 7A), and showed minimal overlap with C3 and C4 (Figure 7B), highlighting the distinct transcriptional consequences of K203X in monocytes.

Pathway enrichment analysis of GO Biological Process categories showed that, in addition to innate immune activation, pattern recognition receptor signaling, and canonical NF-κB signaling, C5 monocytes were enriched for pathways involving endosomal transport, macroautophagy, Golgi vesicle transport, cellular catabolism, and oxidative stress (Figure 7C and Supplemental Figure 5A). GO Cellular Component enrichment further implicated vesicular trafficking and the Golgi apparatus (Supplemental Figure 5B). Network analysis of the top 96 C5-enriched Biological Process pathways grouped these signals into core functional categories involving vesicular trafficking, protein catabolism, autophagy and catabolic processes, starvation and stress responses, NF-κB signaling, innate immune activation, antiviral response, leukocyte differentiation, transcription/RNA processing, and mitochondrial gene expression (Figure 7D). Many of these categories reflected cellular events that could follow accumulation of mislocalized or damaged proteins, including vesicular trafficking defects, enhanced protein catabolism, autophagy-associated processes, starvation-like responses, and stress-response pathways. The parallel enrichment of NF-κB signaling, innate immune activation, response to virus, and leukocyte differentiation indicated that these stress-associated programs were accompanied by inflammatory and immune-activation pathways.

Module scoring further supported this pattern. C5 monocytes showed reduced scores for Golgi vesicle transport and vesicle-mediated transport to the plasma membrane, suggesting downregulated or dysfunctional Golgi protein sorting, impaired vesicle trafficking, and suppression of the secretory pathway (Figure 7E). Reduced macroautophagy gene module scores suggested a blocked or dysregulated autophagic process, while elevated scores for cellular catabolism, ER stress, and oxidative stress indicated activation of stress-adaptation and degradation programs (Figure 7, E and F, and Supplemental Figure 5C). Elevated scores for Toll-like receptor signaling, canonical NF-κB signaling, and innate immune activation further suggested that cellular stress may intersect with inflammatory sensing pathways. Enhanced catabolism, proteasomal degradation, or autophagy can promote accumulation of damage-associated molecular patterns (DAMPs), including ATP, HMGB1, mitochondrial DNA, and oxidized proteins, which may activate pattern recognition receptors and propagate NF-κB–dependent innate immune activation. These altered module scores were specific to C5 and were not observed in the other sJIA-associated monocyte clusters (Figure 7F and Supplemental Figure 5C).

Analysis of the top 96 C5-enriched pathways across major PBMC subsets showed similar but weaker enrichment of vesicle-trafficking, stress-response, and inflammatory pathways in other K203X PBMC subsets, especially CD4^+^ and CD8^+^ T cells, whereas corresponding subsets from other patients with sJIA showed little enrichment beyond limited immune activation in NK and CD8^+^ T cells (Supplemental Figure 5D). The K203X patient also showed expansion of the regulatory T cell compartment within CD4^+^ T cells compared with other patients with inactive sJIA (Supplemental Figure 6). This finding suggested a possible counter-regulatory response occurring alongside broader inflammatory activation, although the functional significance of this expansion was not directly tested in this study.

To identify central genes within these cellular programs, we defined hub genes as those present in at least 15 of the top 96 GO Biological Process pathways. This analysis showed coordinated downregulation of vesicular trafficking regulators, including *AP3B1*, *AP3D1*, *ATG14*, *EPG5*, *LAMP2*, *PIK3C3*, and *PIKFYVE* (Figure 7G). These genes participate in endosomal sorting, autophagosome formation, lysosomal function, and membrane trafficking, supporting broad disruption of vesicle-mediated processes that could impair protein trafficking to the plasma membrane. NF-κB pathway–related hub genes showed increased PRR-associated signaling molecules and adaptors, including *TICAM1*, *PELI1*, *RIPK2*, and *TBK1*, along with increased negative feedback regulators (*TNFAIP3*, *NFKBIA*, *ZC3H12A*) and survival genes (*BIRC2*, *BRD4*). Upregulation of PRR-associated signaling molecules and adaptors suggested an enhanced capacity to sense pathogen- and damage-associated molecular patterns and propagate inflammatory signaling. At the same time, increased negative feedback regulators pointed to an active effort to constrain inflammation. In contrast, *CHUK*, *IKBKB*, and *PYCARD* were downregulated, suggesting simultaneous inflammatory priming, feedback constraint, and dampening of selected NF-κB and inflammasome components. Increased survival genes and reduced mitochondrial stress regulators further supported a dysregulated or stress-adapted monocyte phenotype consistent with chronic immune stimulation, adaptation, or tolerance (Figure 7H). Together, these data support a model in which TNFR1 truncation disrupts membrane trafficking, leading to Golgi retention of the receptor, intracellular stress, enhanced catabolism, and NF-κB–associated cytokine expression independent of intact canonical TNFR1 signaling. This cascade likely contributes to the unique transcriptional program observed in K203X monocytes and to the monocyte-driven inflammatory phenotype in this patient.

### The K203X variant of TNFR1 leads to defects in both TNFR1 signaling and cell death, giving rise to a persistent proinflammatory M1-like monocyte population.

Given the dysregulation of cell death and enhanced survival gene expression, we examined survival characteristics in the monocyte scRNA-seq data. Death receptors *TNFRSF10B* and *FAS* were significantly upregulated in K203X monocytes compared with healthy controls and other patients with sJIA (Figure 8A). Selected proapoptotic components of the intrinsic mitochondrial apoptosis pathway, including *CYCS* and *PMAIP1*, were also upregulated, suggesting increased apoptotic pressure. However, multiple components of the mitochondrial apoptosis machinery (*APAF1*, *TP53*, *BAK1*, *BAX*, and *BBC3*), initiator caspases (*CASP8* and *CASP10*), and executioner caspases (*CASP3* and *CASP6*) were downregulated, while antiapoptotic BCL2-family (*BCL2* and *BCL2A1*) and IAP-family (*BIRC2* and *BIRC3*) genes were upregulated. *SOD2* is induced by oxidative stress with a protective role (35) and was also significantly upregulated. Together with high NF-κB pathway activity, this pattern suggested a mixed apoptotic and prosurvival state with apoptosis-related stress, reduced executioner pathways, and enhanced survival programs. As a result, these monocytes may resist apoptosis despite proapoptotic signaling pressure, promoting survival under stress and potentially contributing to inflammatory persistence. Many of these features were enriched in the patient’s C5 monocyte cluster (Figure 8B). C5 monocytes also showed downregulated S and G2/M phase genes and upregulation of cell-cycle arrest genes, consistent with reduced proliferative capacity and a stress-associated arrest state (Figure 8C).

Based on these findings, we assessed whether altered TNFR1 signaling was associated with impaired cell death in stimulated primary monocytes. In LPS-stimulated PBMCs from age-matched healthy controls (36), CD14^+^ monocytes showed increased cell death, with CD14^+^CD80^+^CD206^–^ M1 monocytes showing the greatest susceptibility (Figure 8, D and E). In contrast, K203X CD14^+^ monocytes were refractory to LPS-mediated cell death regardless of M1 polarization status (Figure 8, D and E). Fas ligand-induced cell death was also defective in K203X monocytes (Figure 8F), consistent with the broader defects in cell-death pathways observed by gene expression (Figure 8, A and B).

Because the inflammatory and prosurvival status of K203X monocytes favored M1 skewing, we assessed M1- and M2-associated monocyte signatures. The K203X-associated C5 cluster showed a strong M1-skewed transcriptional profile (Figure 8G). Upon LPS stimulation, healthy control CD14^+^ monocytes shifted from a substantial M2 population toward a predominant M1 phenotype. K203X PBMCs showed a marked M1 bias at steady state with a concomitant decrease in M2-type cells (Figure 8H). LPS did not further expand the already predominant K203X M1 population but reduced the residual CD80^–^CD206^+^ M2 population, indicating additional capacity for inflammatory skewing upon exposure to activating stimuli.

To determine whether this M1 bias was associated with inflammatory output, we assessed cytokine production in healthy control and K203X PBMCs at steady state and after LPS stimulation. Proinflammatory cytokines were significantly elevated in K203X PBMCs after 24 hours at steady state, including IL-1β, IL-18, IL-6, IL-8, and IL-23 (Supplemental Figure 7), and increased further after stimulation. Cytokines associated with pyroptotic release, such as IL-1β and IL-18, equalized between healthy control and K203X samples after LPS stimulation, possibly related to reduced death in K203X monocyte populations (Figure 8, C and D). Together, these data indicate that PBMCs harboring K203X TNFR1 have impaired cell death and maintain an M1-like proinflammatory CD14^+^CD80^+^CD206^–^ monocyte population capable of persisting and producing inflammatory cytokines.

### TNFR1 variants induce autophagy-associated stress responses in vitro and in primary patient cells.

Given the autophagy-associated transcriptional signatures in K203X C5 monocytes, we assessed the effects of TNFR1 DD variants on autophagy-associated markers in vitro (Figure 7, E and F, Supplemental Figure 5C). Using super-resolution confocal microscopy, we examined localization of the autophagy-associated markers p62 and LC3B (37). In HT1080 cells overexpressing TNFR1 variants, K203X colocalized with both p62 and LC3B (Figure 9, A and B) while remaining sequestered within the Golgi apparatus (Figure 9, A and C). In contrast, the TRAPS variant T50M did not show this pattern (Supplemental Figure 8A), indicating that association with autophagy markers was specific to the DD variants. We next assessed autophagic flux by treating TNFR1-overexpressing cells with bafilomycin A1, which blocks lysosomal acidification and autophagosome degradation. Autophagic flux was calculated as the difference in background-adjusted LC3-II signal between bafilomycin-treated and untreated cells, normalized to β-actin. K203X-expressing cells showed the greatest bafilomycin-sensitive LC3-II accumulation, followed by E398K, while P367S- and ΔDD-transfected cells also showed significant increases compared with WT TNFR1 (Supplemental Figure 8, B and C). These results indicated that autophagy-associated pathways were engaged by the TNFR1 DD variants, with the strongest response observed for the truncating K203X variant and a graded response across the missense variants.

We next assessed cellular stress responses in Jurkat 3T8 cells expressing WT or variant TNFR1. K203X and E398K induced significantly elevated reactive oxygen species compared with WT TNFR1, while P367S showed a more modest increase, indicating variant-dependent oxidative stress associated with TNFR1 DD variants (Supplemental Figure 8G). TNFR1 variants also increased ATF-4 expression and p38 phosphorylation relative to WT TNFR1, indicating activation of integrated stress response/UPR-associated and stress-responsive MAPK pathways (Supplemental Figure 8D, E and F). Because acute TNF-induced canonical NF-κB signaling was impaired by these variants, we assessed whether longer-term variant expression was associated with basal, stress-related NF-κB transcriptional activity independent of acute TNF stimulation. K203X- and E398K-expressing cells showed increased basal NF-κB reporter activity compared with WT TNFR1 (Supplemental Figure 8H).

Finally, we examined whether the autophagy-associated phenotype observed in vitro was present in primary K203X patient cells. In K203X PBMCs, TNFR1 showed increased colocalization with both p62 and LC3B compared with age-matched healthy controls (Figure 10, A and B). Both p62 and LC3B also showed increased colocalization with the Golgi marker GM130, although LC3B formed additional puncta outside the Golgi compartment (Figure 10, A and C). These findings indicate that K203X TNFR1 is associated with autophagy markers in primary patient cells and support a Golgi-associated autophagy phenotype ex vivo. Taken together, the in vitro and primary-cell data indicate that TNFR1 DD variants induce variant-dependent autophagy-associated responses and cellular stress, with evidence of a similar phenotype for K203X in primary cells. The magnitude and character of these responses differed across variants, supporting variant-dependent autophagic dysregulation rather than uniform impairment of autophagy.

## Discussion

In this study, we describe a child with sJIA carrying a de novo truncating variant of *TNFRSF1A* that encodes a form of TNFR1 (K203X) lacking the transmembrane and DDs, together with 2 additional patients carrying ultra-rare missense variants affecting the TNFR1 DD. Functional studies showed variant-dependent impairment of canonical TNFR1 activity, with reduced NF-κB activation and TNF-induced cell death. K203X and E398K exerted greater effects on TNFR1 signaling than P367S, consistent with the precedent that DD mutations at different protein-binding interfaces can differ in signaling consequences (38, 39). However, impaired DD-mediated signaling alone does not explain the full phenotype. Molecular and cellular profiling of K203X patient cells revealed defective TNFR1 trafficking, Golgi retention, impaired proteostasis, disrupted recycling pathways, and stress-associated inflammatory programs. Thus, altered TNFR1 function may drive inflammation not only through impaired canonical TNFR1 signaling, but also through intracellular mislocalization, organelle stress, and monocyte-driven inflammatory activation.

Although these variants affect TNFR1, the clinical and mechanistic phenotype diverges from TNFR1-associated periodic fever syndrome (TRAPS). TRAPS is typically autosomal dominant, periodic, and relapsing-remitting, with pathogenic variants concentrated in extracellular cysteine-rich domains and often associated with ER retention and enhanced inflammatory signaling independent of TNF stimulation (40, 41). In contrast, P367S and E398K affect the intracellular DD, whereas K203X eliminates both the transmembrane and DDs. The child with K203X had a monophasic sJIA course, lacked periodic febrile episodes, and remains in sustained treatment-free remission; the P367S and E398K carriers also lacked typical TRAPS features. Mechanistically, K203X predominantly accumulates in the Golgi rather than the ER and impairs NF-κB signal transduction and TNF-induced apoptosis, despite upregulation of some NF-κB–induced inflammatory programs. This is particularly notable because *TNFRSF1A* is highly intolerant of loss-of-function variation, with a probability of LOF intolerance (pLI) of 1.0 in gnomAD (42). The phenotype also appears distinct from simple haploinsufficiency: *Tnfrsf1a*-deficient mice display increased hematopoietic stem cells, myeloid precursors, and mature myeloid cells (43, 44), whereas the K203X patient had relative monocytopenia, implicating additional mechanisms related to aberrant receptor trafficking and cellular stress.

A central feature of K203X biology is Golgi accumulation. The Golgi apparatus is a hub for protein sorting, posttranslational modification, and intracellular signaling (45), and disruption of Golgi function can impair trafficking, induce cellular stress, and activate innate immune pathways. K203X likely enters the secretory pathway but, lacking a transmembrane domain, fails to traffic appropriately and accumulates within the Golgi. Transcriptomic data supported downregulation of Golgi- and vesicular trafficking–associated processes without strong activation of a classical ER-misfolding response, suggesting a trafficking-centered mechanism. A related principle has been described in NOCARH syndrome, where Golgi-retained CDC42 variants disrupt Golgi homeostasis and trigger innate immune activation/type I interferonopathy (46). In contrast with intracellular CDC42, however, TNFR1 is normally surface trafficked and is most highly expressed among PBMC subsets in monocytes, a major source of sJIA-associated cytokines including IL-1β. This context may explain why Golgi stress caused by K203X produces a monocyte-dominant inflammatory phenotype, although broader TNFR1 expression raises the possibility that related stress responses occur in other immune and nonimmune cells. The presence of similar, albeit weaker, Golgi abnormalities in cells expressing P367S and E398K supports a shared but variant-dependent trafficking phenotype rather than a mechanism restricted to K203X alone.

The autophagy findings extend this model. Autophagy maintains cellular homeostasis by removing damaged organelles and macromolecules and helps resolve inflammation by clearing activated signaling complexes (47). Disrupted autophagic processing can promote accumulation of danger-associated molecular patterns (DAMPs), impair inflammasome clearance, and increase IL-1β production (48). In the K203X patient, transcriptomic analysis suggested altered cellular recycling, and functional studies demonstrated autophagy-associated responses with persistent Golgi retention, cellular stress, and unfolded protein response signaling. These findings suggest that autophagic pathways are engaged but insufficient to resolve the burden of mislocalized TNFR1, producing ongoing proteostatic stress rather than simple absence of autophagy. Impaired autophagy has been implicated in sJIA and adult-onset Still’s disease (AOSD) (49–52), including impaired autophagic flux in AOSD immune cells that correlates with disease activity (49), *LACC1* loss-of-function variants that impair macrophage autophagy in monogenic sJIA (50), and constitutive mTORC1 activation from *Tsc2* deficiency that produces Still’s disease–like inflammation (51). Our data expand this framework by suggesting that autophagic dysregulation can arise from defective trafficking of a surface receptor. In this setting, unresolved cargo, organelle stress, and DAMP/PRR-associated signaling may propagate NF-κB–driven IL-1β and TNF expression, particularly when programmed cell death is impaired.

One paradox is that the K203X patient remained clinically inactive despite a hyperinflammatory monocyte transcriptional profile. Peripheral monocytopenia may partly explain this compensated state by limiting the size of the inflammatory compartment. At the same time, remaining monocytes strongly resisted LPS- and Fas-induced cell death ex vivo, and their transcriptional profile showed oxidative-stress and prosurvival adaptation with downregulation of cell-death pathways. Impaired Fas-mediated apoptosis has been described in sJIA monocytes and neutrophils, supporting resistance to cell death as a disease-contributing feature (49). K203X monocytes also displayed cell-cycle arrest and reduced proliferative signatures, suggesting impaired production or maintenance of the monocyte compartment. These findings imply that remission in this patient reflects compensated inflammation rather than full restoration of immune homeostasis, consistent with the idea that monophasic sJIA may resolve into a new immunologic balance rather than a predisease state (7). This interpretation may also help reconcile the patient’s sustained remission with persistent cellular abnormalities, because inflammatory potential may remain detectable even when the circulating monocyte compartment is quantitatively constrained.

This phenotype also resembles a senescence-like stress program. Cellular senescence is characterized by proliferative arrest, apoptosis resistance, and acquisition of a proinflammatory secretory phenotype (53, 54), and can occur in immune cells under conditions of impaired proteostasis and chronic stress (55). The K203X-associated C5 monocyte cluster showed inflammatory cytokine production, apoptosis resistance, cell-cycle arrest, and impaired organelle recycling. Senescence-associated changes in myeloid progenitors can impair monocyte production and generate functionally abnormal myeloid cells (56, 57), raising the possibility that K203X-induced proteostatic stress contributes to both monocytopenia and abnormal monocyte function. We do not propose that the C5 population is equivalent to canonical senescence, but rather that it shares several features of a stress-adapted, apoptosis-resistant, inflammatory state.

Although the C5 monocyte population was unique to the K203X patient in this dataset, aspects of this phenotype may be relevant to broader inflammatory or stress-adapted monocyte states. Golgi abnormalities were also observed after overexpression of E398K and P367S, albeit to a lesser extent, and several patients with inactive sJIA displayed atypical monocyte clusters distinct from C5. Trafficking defects, autophagic dysregulation, and stress-induced inflammation are also features of protein misfolding disorders such as cystic fibrosis and neurodegenerative diseases (58–60). Our findings therefore expand this paradigm to autoinflammation and suggest that intracellular trafficking and proteostasis defects can manifest as systemic immune dysregulation. They also raise the possibility that, in selected Still’s disease endotypes, organelle health and proteostasis may be therapeutically relevant alongside downstream cytokine blockade. This broader implication should be interpreted cautiously, because the strongest ex vivo evidence comes from a single K203X carrier and the missense variants were studied primarily in overexpression systems.

Several limitations should be considered. We were unable to identify a larger TNFR1-DD variant cohort despite screening the INCHARGE Consortium and NIH Still’s disease/autoinflammatory disease cohorts, likely reflecting intolerance of TNFR1 to LOF variation. Only the K203X patient was available for detailed investigation; P367S and E398K carriers were unavailable for follow-up, and K203X sample acquisition was limited by age. The scRNA-seq analyses compared an untreated K203X patient in inactive disease with inactive sJIA datasets from treated patients, so treatment differences may have influenced transcriptional profiles. In vitro assays helped address limited primary material, and P367S/E398K showed behavior broadly consistent with K203X across multiple systems, but the nonmyeloid cell lines used here do not fully model monocyte cytokine production or inflammasome-dependent IL-1β release. Variant-expressing myeloid models will be needed to define how cellular stress responses produce the K203X monocyte cytokine phenotype. In addition, the E398K patient did not meet full ILAR criteria for sJIA and had uveitis, an atypical feature, although febrile debut and persistent polyarthritis support classification as Still’s like. Limited historical clinical information and absence of active-disease IL-18 measurements reduce diagnostic certainty for this case. Thus, these variants should be viewed as contributors to altered inflammatory biology and potential disease pathophysiology rather than as deterministic monogenic causes of classic sJIA.

In summary, K203X TNFR1 reveals a mechanism of Still’s disease–associated inflammation driven by defective protein trafficking, Golgi retention, autophagy-associated stress, and abnormal monocyte homeostasis. Unlike TRAPS, K203X impairs canonical receptor function while paradoxically promoting inflammatory programs through intracellular accumulation and unresolved proteostatic stress. These findings challenge a purely cytokine-centered view of sJIA by highlighting organelle health, proteostasis, and trafficking as potential determinants of disease biology. More broadly, they illustrate how rare genetic variants can uncover clinically relevant inflammatory mechanisms that may persist even during apparent remission and shape disease heterogeneity across the Still’s disease spectrum.

## Methods

### Sex as a biological variable.

Our study examined both male and female pediatric participants. As patients were prepubescent at time of sample collection, sex as a variable was not considered and not examined.

### Study approval.

Human-subjects research was approved by the National Institutes of Health Institutional Review Board (Bethesda, Maryland, USA; NIH protocol AR-18-0081). Written informed consent was obtained from adult participants or from the parents or legal guardians of pediatric participants, and assent was obtained from minors when applicable. DNA samples from the INCHARGE Consortium were included under a nonhuman subjects research exemption by the National Institutes of Health Institutional Review Board.

### Statistics.

Statistical analyses are described in the figure legends and Supplemental Methods. Exact *n* values and definitions of independent experiments are provided in the corresponding figure legends. Multi-group comparisons in in vitro, imaging, immunoblot densitometry, and flow-cytometry experiments were performed by 1-way ANOVA unless otherwise indicated. Serum sTNFR1 concentrations were compared using the Mann-Whitney test. For single-patient primary-cell comparisons, z-scores were calculated relative to the healthy-control distribution. Cytokine bead array data were normalized to absolute monocyte number; technical replicates were averaged by donor or sampling occasion, and the K203X case-level mean was compared with healthy controls using the Crawford-Howell modified single-case *t* test. For scRNA-seq, differentially expressed genes were identified using Seurat FindMarkers with Benjamini-Hochberg adjustment, average log_2_ fold change > 0.5, minimum percentage of expressing cells > 10%, and adjusted *P* < 0.1. Individual cells were not considered independent biological replicates for module-score or cluster-level comparisons; analyses lacking adequate subject-level replication were therefore presented descriptively without inferential statistical testing. *P* < 0.05 was considered statistically significant unless otherwise specified.

### Data Availability.

Single cell RNA-seq data from the K203X patient have been deposited in the database of Genotypes and Phenotypes (dbGaP) under accession phs004986.v1.p1. Previously published exome sequencing data from the K203X proband and family were reported in Marques MC et al. (52) and are available through dbGaP (accession phs004962.v1.p1). Values underlying graphed data are provided in the Supporting Data Values file. Previously published scRNA-seq datasets used for comparison are described in the Supplemental Methods.

Detailed descriptions of all methodologies used are available in the Supplemental Material.

## Manuscript information

### Author contributions.

ACC, KC, MJO designed and conceived of all experiments. ACC, KC, PMP, MJO wrote and edited the manuscript. ACC performed in vitro and ex vivo experiments. PMP, HN performed in vitro experiments and assisted in study design. CF performed in vitro experiments. ACC, MK performed Western blot experiments and analyses. SRC performed ELISA experiments. DF, RSMY, PFW provided patient samples and assisted in study design. FN, SD performed RNA library preparation and scRNA sequencing. MCM, LLB, MM, CL generated clinical data and provided patient care. KN, KY assisted in study design and data analysis. ELV, GS provided in silico scRNA sequencing data. ACC, DR performed super resolution imaging. TF, HW performed molecular modeling of TNFR1 receptors. KC, BU, EDR, PS, VC, JL, ZD performed all bioinformatics analyses and assisted in experimental design. MJO involved in all elements of this study. All co-authors edited and commented on the final manuscript.

### AI disclosure.

The manuscript was conceived and initially drafted by the authors without artificial intelligence (AI) assistance. ChatGPT (OpenAI; GPT-4o, GPT-4.1, and GPT-4.5, April-June 2025; GPT-5.2, GPT-5.3, and GPT-5.5, May–August 2026) was subsequently used to support manuscript revision through language refinement and word count reduction while preserving the authors’ intended scientific meaning. ChatGPT (GPT-4o, April–June 2025) was also used to assist with drafting and troubleshooting command-line and R script syntax for implementing established single-cell RNA sequencing analysis workflows. ChatGPT (GPT-5.6 Sol Pro, October 2026) was used to conceptualize and generate the graphical abstract. AI tools were not used to independently execute data analyses. The authors reviewed and edited all AI-assisted text, validated the analytical code and results, and critically evaluated all scientific interpretations. The authors assume full responsibility for the accuracy and integrity of the final manuscript, including the graphical abstract.

### Conflict of interest.

RY has been a one-time symposium speaker at Sobi, has attended a conference at Amgen, and has worked on industry-sponsored clinical trials for Amgen and Astra Zeneca. PW has received royalty and license fees from Up-to-date (< $10K to author), has been the site investigator for Pfizer and Abbvie Clinical Trials (with payment to institution), has received consulting fees from Pfizer and Bristol Meyers Squibb (all < $5K to author), and has received speaking payment or honoraria for educational materials from Spondyloarthritis Research and Treatment Network, Psoriasis Foundation, and Pfizer (all < $5k to author). GS has received consulting fees from Novartis and SOBI (all < $5k) and research support from IpiNovyx.

### Funding.

This study was funded by:

- The Intramural Research Program of the National Institute of Arthritis and Musculoskeletal and Skin Diseases (Z01-AR041198).

## Supplementary Material

Supplemental data

Unedited blot and gel images

Supporting data values

## Figures and Tables

**Figure 1:**
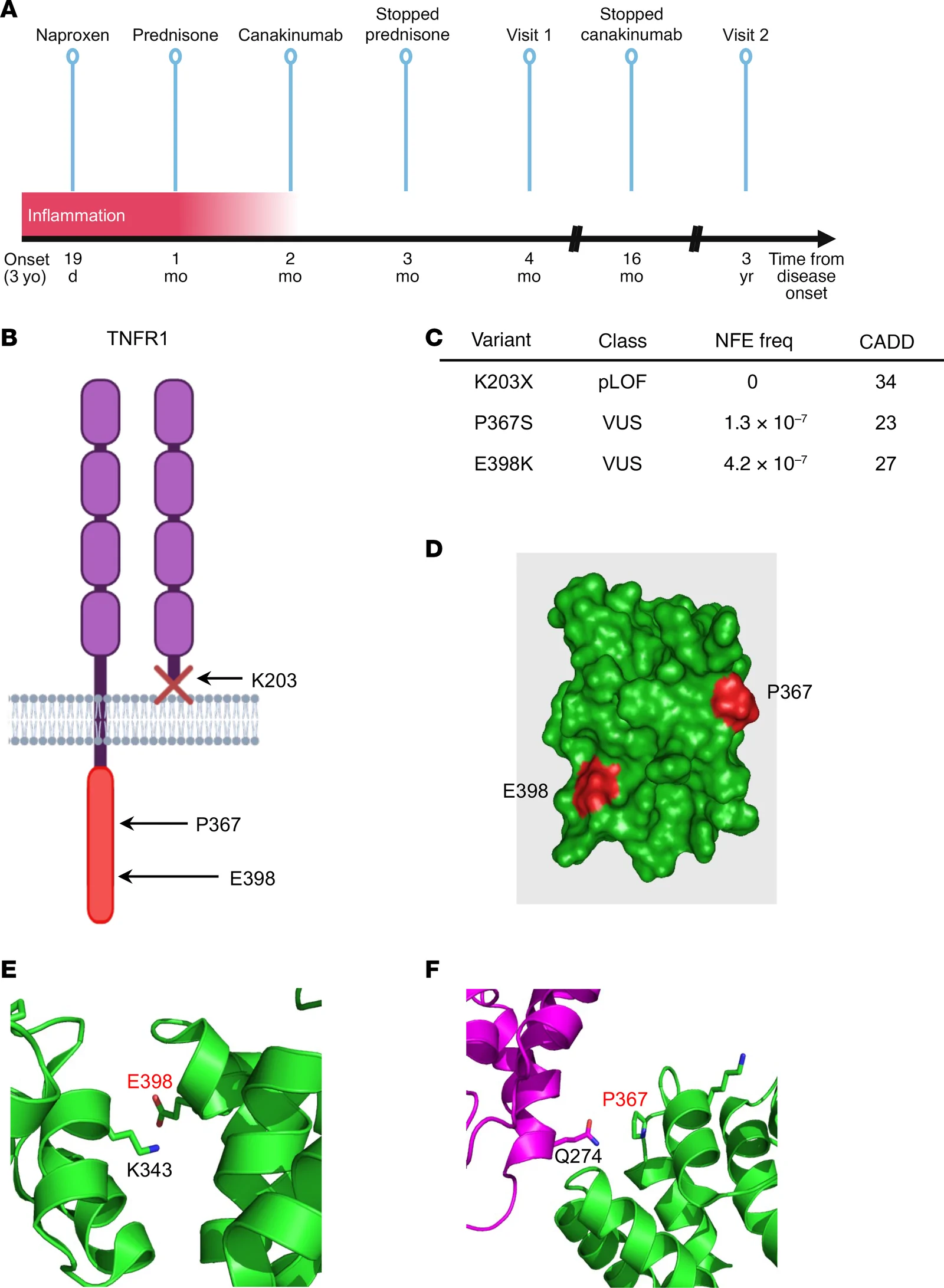
Modeling of rare TNFR1 variants identified in patients with Still’s disease. (**A**) Schematic showing key clinical events in the disease course of a child with monophasic sJIA. (**B**) Schematic representation of TNFR1 showing location of K203 truncation variant and the P367S and E398K DD variants. (**C**) In silico characteristics of TNFR1 variants identified in patients with Still’s disease. (**D**) 3D globular structure of TNFR1 DD (green) and location of P367S and E398K (red). (**E** and **F**) Predictive structural views of the modeled TNFR1 DD interface, showing E398 and K343 in adjoining TNFR1 DDs (**E**) and P367 near TRADD residue Q274 at the TNFR1 DD-TRADD interface (**F**).

**Figure 2:**
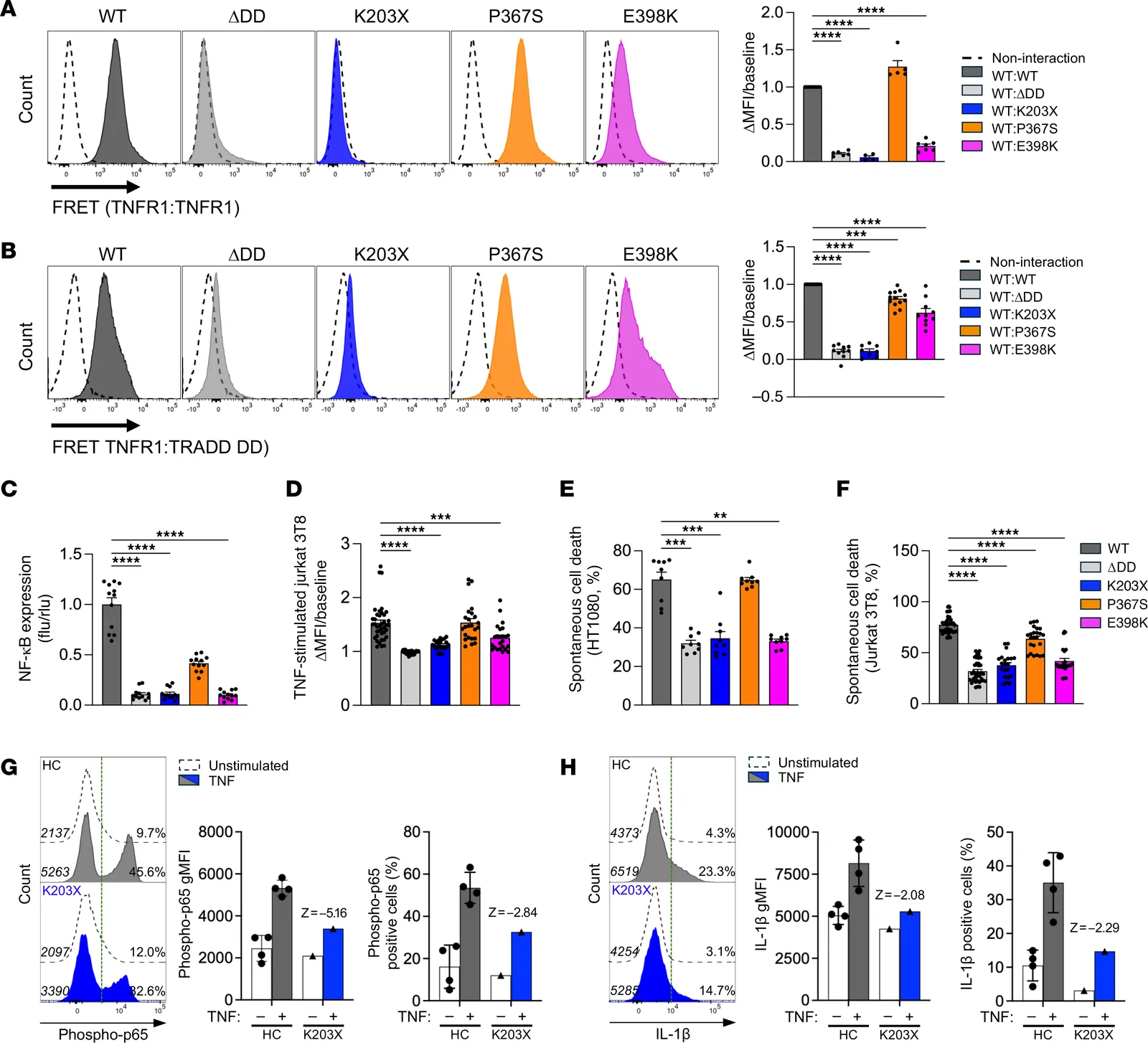
TNFR1 variants disrupt receptor interactions and downstream signaling. (**A**) HEK293T cells were transfected with Cerulean3- or Venus-tagged WT or variant TNFR1 and analyzed for interactions by FRET flow cytometry. (**B**) Cells were transfected with a truncated TRADD containing only the DD together with WT or variant TNFR1, and analyzed by FRET as in **A**. Representative histograms are shown for **A** and **B**. *N* ≥ 5 independent experiments. ***P < 0.001; ****P < 0.0001 by ANOVA. (**C**) HEK293T cells were transfected with WT or TNFR1 variants and assayed for NF-κB activity by luciferase reporter, normalized to *Renilla* luciferase. *N* > 5 independent experiments, ****P < 0.0001 by ANOVA. (**D**) Jurkat 3T8 NF-κB reporter cells expressing Venus-tagged WT or variant TNFR1 were incubated with or without TNF (1 ng/mL), and NF-κΒ signaling was measured by flow cytometry in viable, Venus^+^ cells. *N* > 5 independent experiments, ***P < 0.001; ****P < 0.0001 by ANOVA. (**E**) HT1080 cells expressing Venus-tagged WT or variant TNFR1 were stained with DiIC (5) and Live/Dead Blue and analyzed by flow cytometry. ****P < 0.0001 by ANOVA. (**F**) Jurkat 3T8 cells expressing Venus-tagged WT or mutant TNFR1 were stained with Annexin V and Live/Dead Blue and analyzed by flow cytometry. ****P < 0.0001 by ANOVA. (**G**) PBMCs from healthy controls (HC, *N* = 4) and a patient carrying the K203X (*N* = 1) were left unstimulated or stimulated with TNF and analyzed for phospho-p65 by intracellular flow cytometry. Histograms show unstimulated (dashed) and TNF-stimulated cells (filled; grey, HC; blue, K203X). Green dashed line indicates the positivity threshold. gMFI and percent positive cells are indicated. Summary plots show individual HC values with mean ± SD and the K203X patient value. Z-scores indicate deviation of K203X from TNF-stimulated HCs. (**H**) PBMCs were stimulated with TNF and analyzed for intracellular IL-1β. Histograms and quantification of IL-1β gMFI and IL-1β^+^ cells are shown as in **G**.

**Figure 3:**
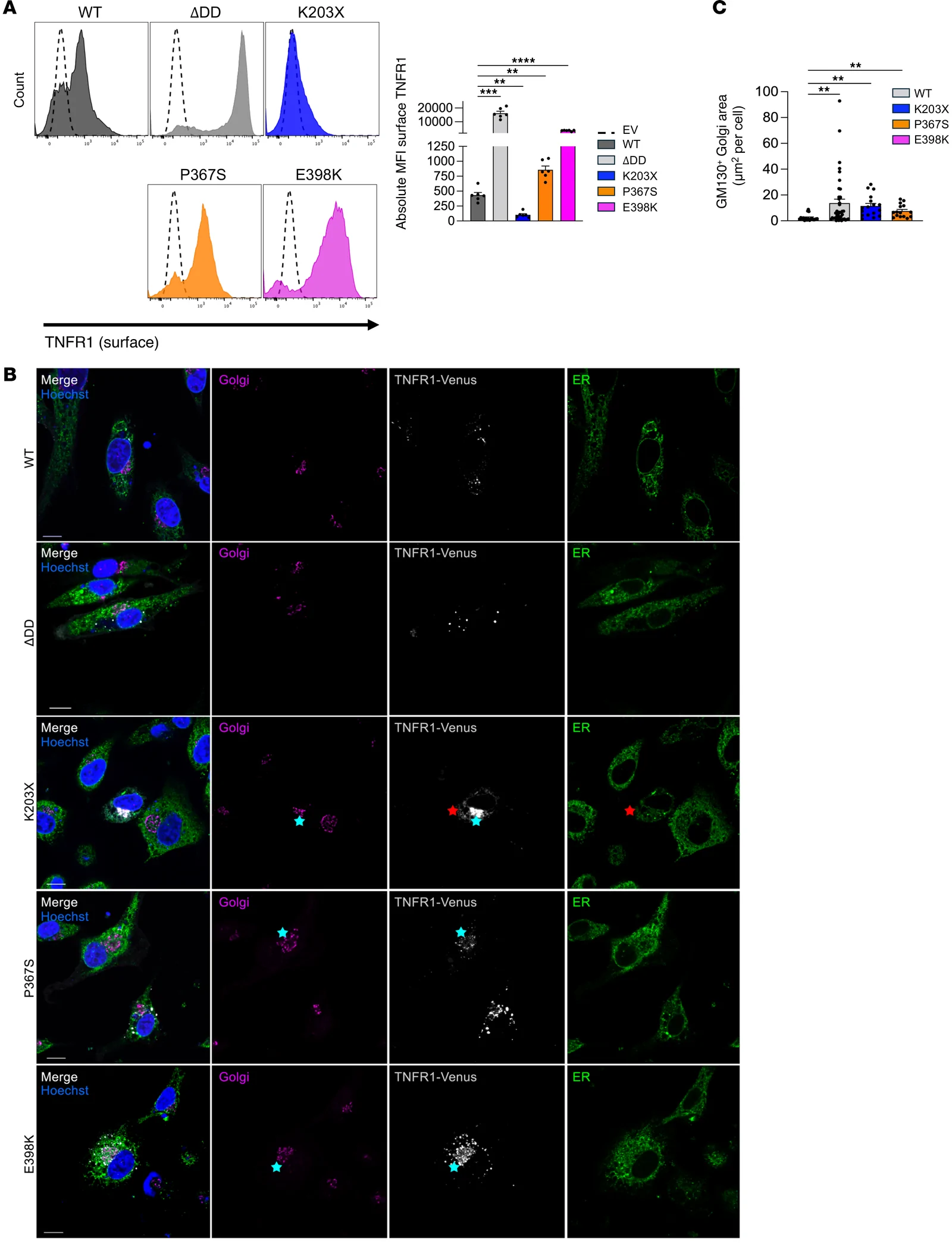
Variant TNFR1 K203X localizes preferentially to the Golgi complex. (**A**) Analysis of TNFR1 on the cell surface. HT1080 cells were transfected with Venus-tagged WT or variant TNFR1. 18 hours after transfection, cells were stained with anti-TNFR1 and surface expression was measured by flow cytometry, with absolute change in MFI quantified. Representative histograms are shown, and data are cumulative of *N* = 6 experiments. **P < 0.01; ***P < 0.001; ****P < 0.0001 via ANOVA. (**B**) HT1080 cells were transfected with Venus-tagged WT or variant TNFR1 (as indicated; gray). 18 hours after transfection, cells were fixed and stained with antibodies to GM130 (to detect Golgi; magenta) and KDEL (endoplasmic reticulum, ER; green). Cells were counterstained with Hoechst 33342 (blue) for nuclear detection and imaged via super-resolution confocal microscopy. Red star represents localization of TNFR1 with the ER. Cyan star shows TNFR1 localization with the Golgi. Images are representative of *N* ≥ 6 fields captured, and *N* = 3 independent experiments. Scale bar: 10μm. (**C**) Golgi area, as indicated by staining of GM130, was determined in minimum of 15 representative cells from each individual examined in **B**. **P < 0.01 by ANOVA.

**Figure 4:**
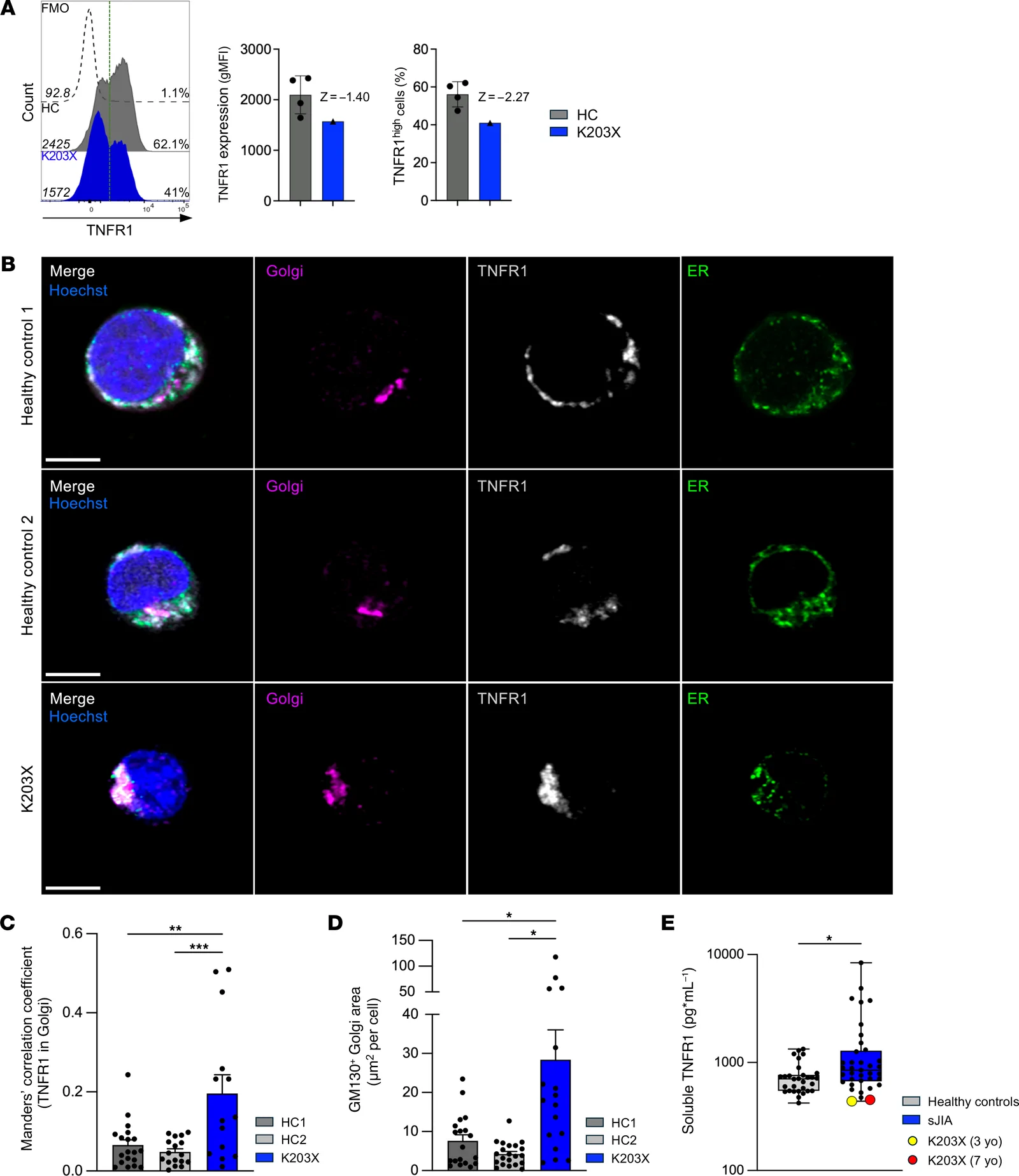
TNFR1 is retained predominantly in the Golgi of cells in a patient harboring the K203X variant. (**A**) Surface expression of TNFR1 in PBMC monocytes from healthy controls (HC, *N* = 4) or a patient carrying the K203X variant (K203X, *N* = 1). Representative histograms show TNFR1 surface expression in monocytes (grey, HC; blue, K203X; dash line, TNFR1 fluorescence minus one control staining (FMO)). The vertical green dashed line indicates the positivity threshold, defined based on the FMO. Geometric mean fluorescence intensity (gMFI; italicized) and the frequency of positive cells (%) are indicated on the histograms. Summary plots show TNFR1 gMFI (left) and the percentage of TNFR1^+^ cells (right). HC data are shown as mean ± SD with individual values overlaid; K203X is shown as a single value. For single-patient comparison, Z-scores were calculated to quantify the deviation of the patient from the HC distribution under TNF-stimulated conditions and are indicated on the summary plots (Z). (**B**) PBMCs from a patient carrying the K203X variant and 2 age-matched healthy controls were fixed and stained with antibodies to GM130 (magenta), KDEL (green), and anti-TNFR1. Cells were counterstained with Hoechst 33342 to visualize nuclei (blue). Representative images are shown of *N* ≥ 5 fields captured. Scale bar: 10μm. (**C**) Manders’ correlation coefficient of TNFR1 within the Golgi, with *N* ≥ 14 representative cells from each individual used for calculation. **P < 0.01; ***P < 0.001 via ANOVA. (**D**) Golgi area was determined in 20 representative cells from each individual examined in **B**. *P < 0.05 via ANOVA. (**E**) Serum samples collected from patients with sJIA (*N* = 62) and healthy children (*N* = 30) from the NIH cohort were assayed for soluble TNFR1 (sTNFR1) by ELISA. Measurements from the patient with the K203X variant are displayed as large yellow (first visit) and red (second visit) circles. *P < 0.05 by Mann-Whitney.

**Figure 5:**
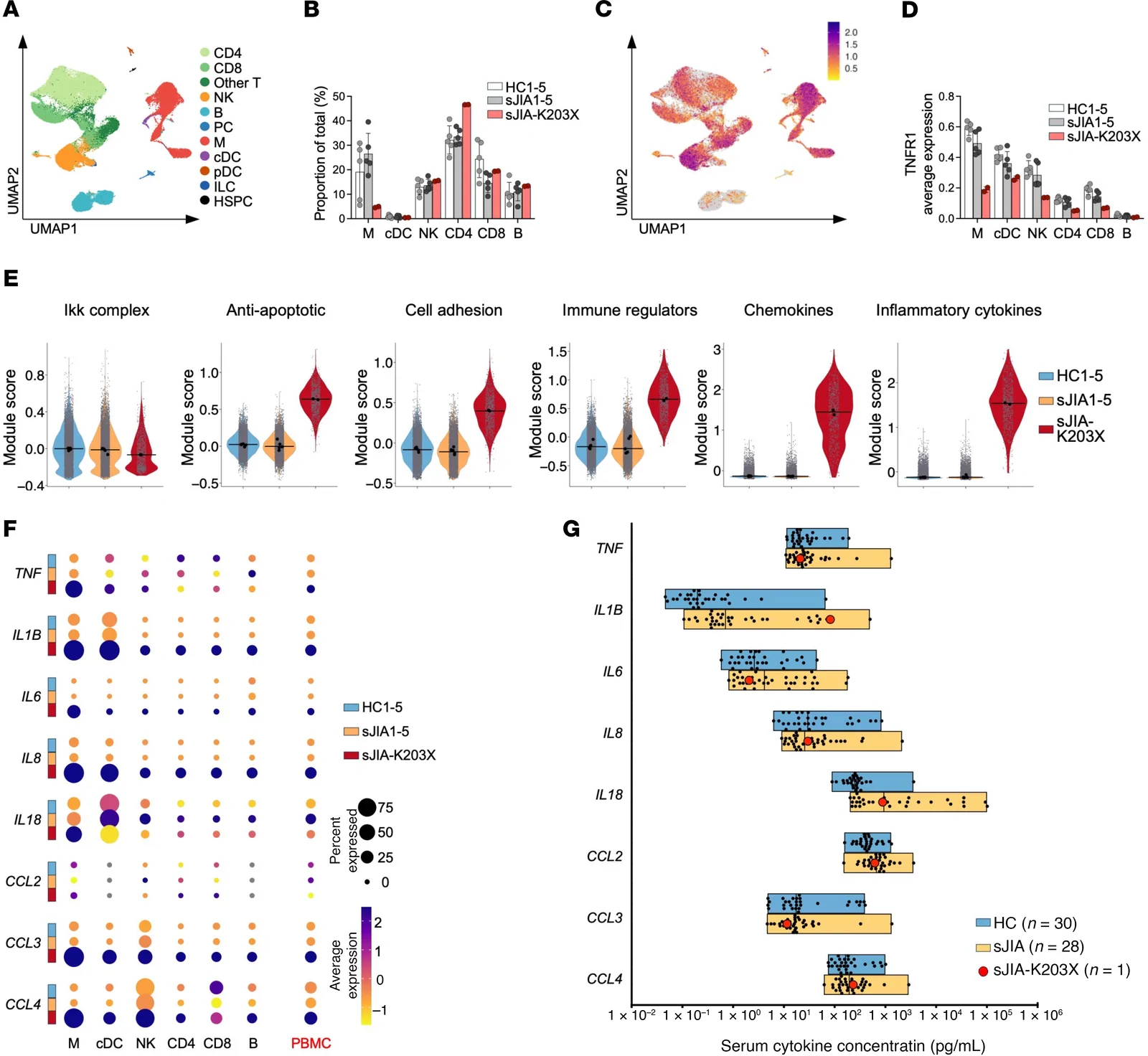
Single-cell RNA-seq of PBMCs from a patient carrying the TNFR1 K203X variant identifies a unique proinflammatory gene expression signature in monocytes. (**A**) UMAP of 91,950 PBMCs from 5 healthy controls and 6 patients with sJIA. The sJIA-K203X patient is shown separately using 2 technical replicates from the second visit (age 7 years). (CD4, CD4^+^ T cells; CD8, CD8^+^ T cells; Other T, double negative T cells, gamma delta T cells and mucosal-associated invariant T cells; B, B cells; PC, plasmablasts and plasma cells; M, monocytes; cDC, conventional dendritic cells; pDC, plasmacytoid dendritic cells; ILC, innate lymphocytes; HSPC, hematopoietic stem and progenitor cells.). (**B**) Proportions of major PBMC subsets by groups. (**C**) Feature plot of normalized *TNFRSF1A* expression overlaid on the UMAP from panel **A**. (**D**) Normalized *TNFRSF1A* expression across major PBMC subsets by groups. (**E**) Violin plots showing module scores for the IKK complex and five NF-κΒ–related gene sets in healthy controls (HC1-5), patients with sJIA (sJIA1-5), and the sJIA-K203X patient. Cells are shown in grey, individual medians as large black dots, and group medians horizontal lines. (**F**) Bubble heatmap showing inflammatory cytokine and chemokine expressions in the monocytes (M), conventional dendritic cells (cDCs), NK cells (NK), CD4^+^ T cells (CD4), CD8^+^ T cells (CD8), B cells (B) and total PBMC cells (PBMC) in healthy controls (HC1-5), patients with sJIA (sJIA1–5) and the sJIA-K203X patient. (**G**) Horizontal floating bar plot showing the serum concentrations of inflammatory cytokines and chemokines in healthy controls (*N* = 30), patients with sJIA (*N* = 28), and the sJIA-K203X patient at the second visit (age 7 years). Bars represent the range (min to max), lines indicate the median, and individual values are shown as black dots for healthy controls and patients with sJIA. The sJIA-K203X values are highlighted by large dark red dots.

**Figure 6:**
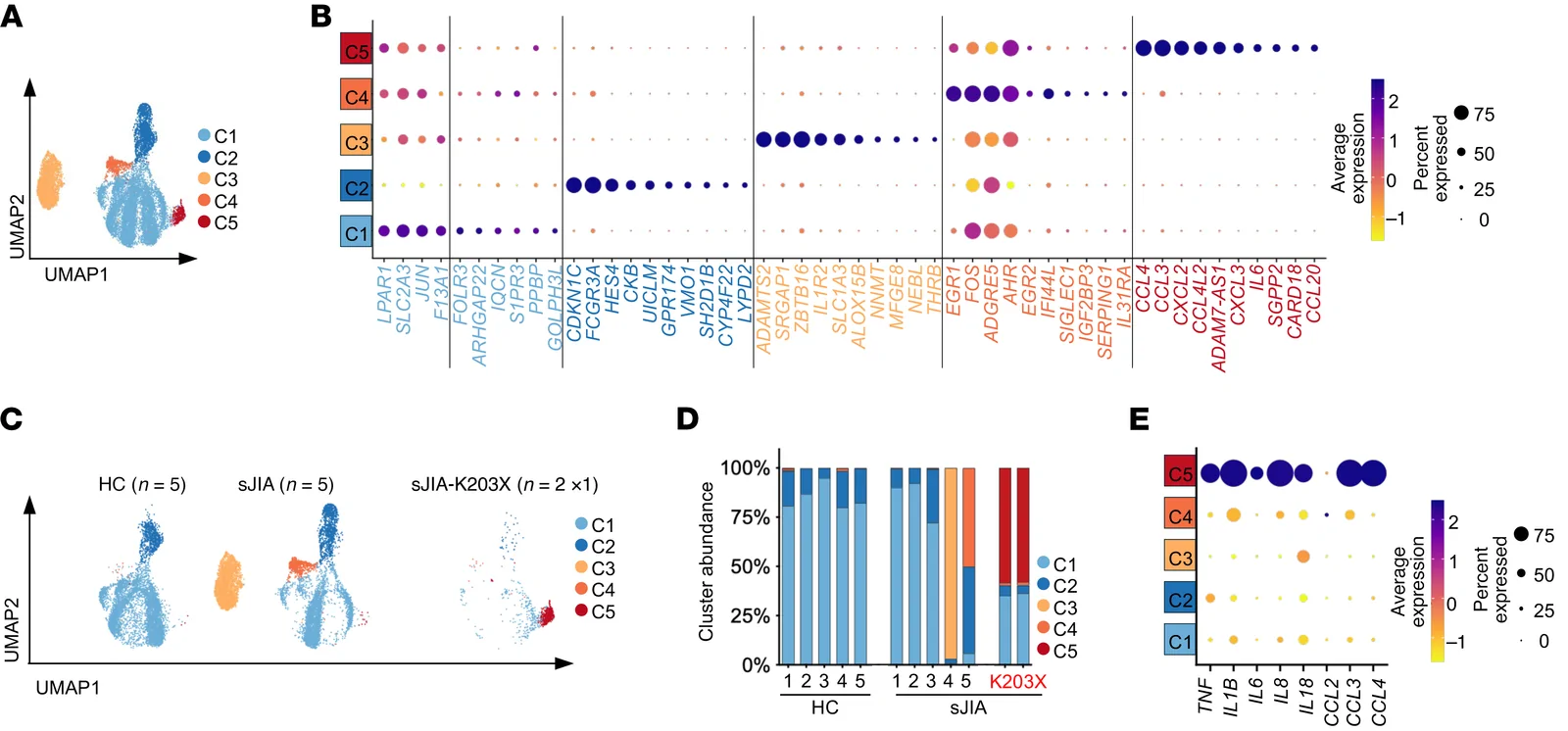
K203X TNFR1 monocytes display a unique gene signature that integrates dysfunctional endosomal transport and autophagy with enhanced stress responses and innate immune activation. (**A**) UMAP plot showing clustering of 22,575 monocytes subsetted from panel Figure 5**A**. (**B**) Bubble heatmap showing the expression of the top 10 cell-type–specific markers across monocyte clusters (C1–5). (**C**) UMAP plot showing monocyte distribution across clusters by group (healthy controls, sJIA patients, and the sJIA-K203X patient). (**D**) Distribution of monocytes across clusters by sample. (**E**) Expression of inflammatory cytokines and chemokines across monocyte clusters.

**Figure 7:**
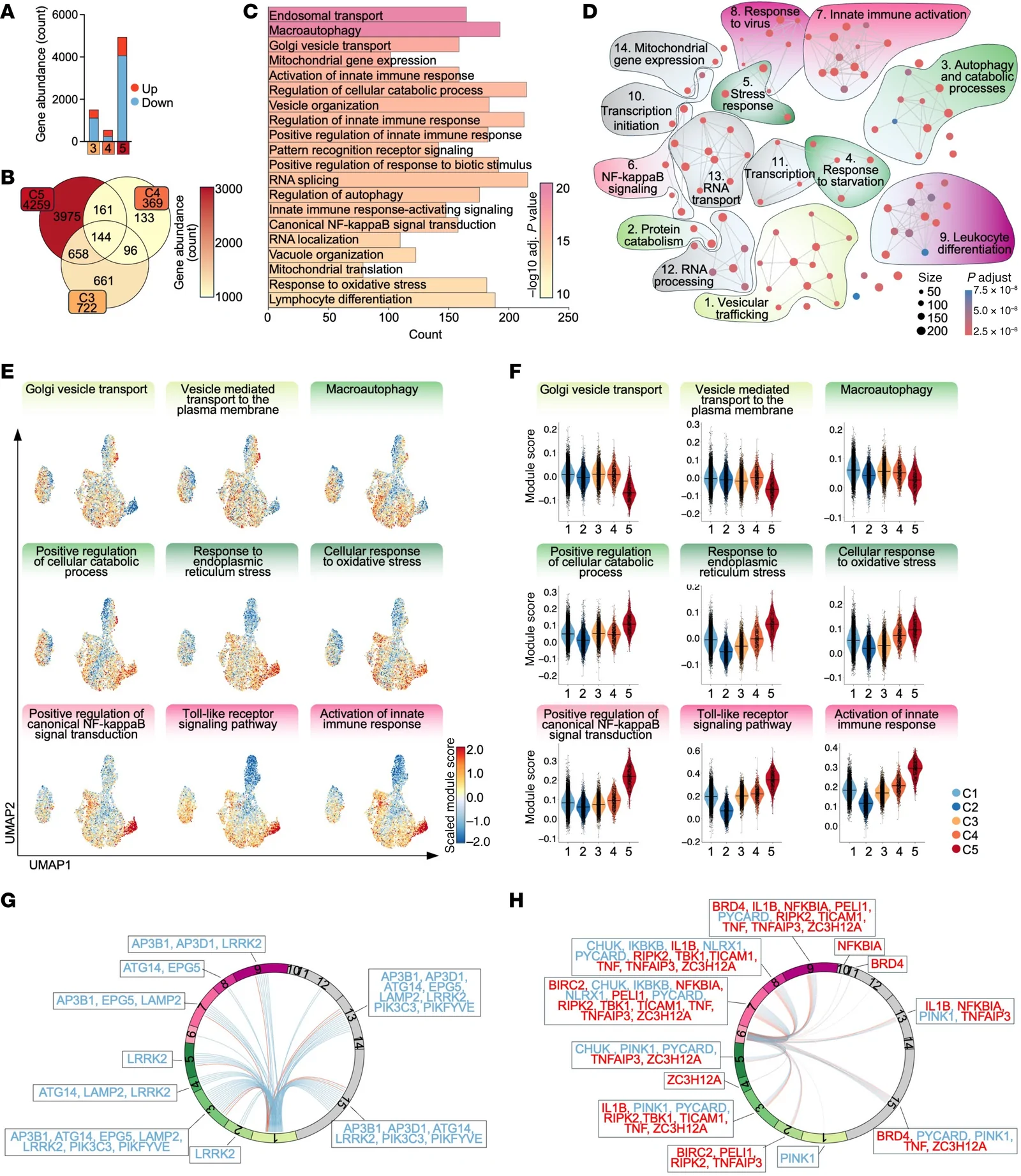
Pathway analysis identifies vesicular trafficking, stress-response, and inflammatory programs in the K203X-enriched monocyte cluster. (**A**) Number of upregulated and downregulated genes in sJIA-specific monocyte clusters (C3–5) compared with classical monocytes (C1). (**B**) Venn diagram shared and unique DEGs among sJIA-specific monocyte clusters. Cluster labels (C3–C5) indicate the total number of DEGs per cluster; color intensity reflects gene number. (**C**) Top 20 significantly enriched Gene Ontology (GO) Biological Process (BP) terms among DEGs between the sJIA-K203X–specific monocyte cluster (C5) and classical monocytes (C1). Bar length indicates gene count, color gradient reflects the adjusted *P* value (p.adjust). (**D**) Enrichment map (emapplot) displaying clusters of functionally related GO biological process (BP) pathways in monocytes. The top 96 significantly enriched pathways (adjusted *P* value < 1×10^–8^) are shown. Each node represents a pathway, with node size corresponding to the number of DEGs involved. Node color reflects statistical significance (adjusted *P* value), with red indicating higher significance. Edges represent related pathways that are grouped by color and labeled according to core pathway themes. (**E**) Feature plots showing module scores for 9 relevant pathways in monocytes overlaid on the UMAP shown in panel **A**. (**F**) Violin plots showing module scores of 9 relevant pathways across monocyte clusters. Individual cells are shown as black dots, and the median of all cells within each cluster is indicated by a horizontal line. (**G** and **H**) Circos plots showing the crosstalk and shared relevant hub genes between core pathway 1 (Vesicular trafficking) and core pathways 2–15; and core pathway 6 (NF-κB signaling) and core pathways 1–5 and 7–15, respectively. Chords connect relevant genes shared across multiple categories. The color of chords and gene names indicates expression direction in C5 vs. C1 (blue: downregulated; red: upregulated).

**Figure 8:**
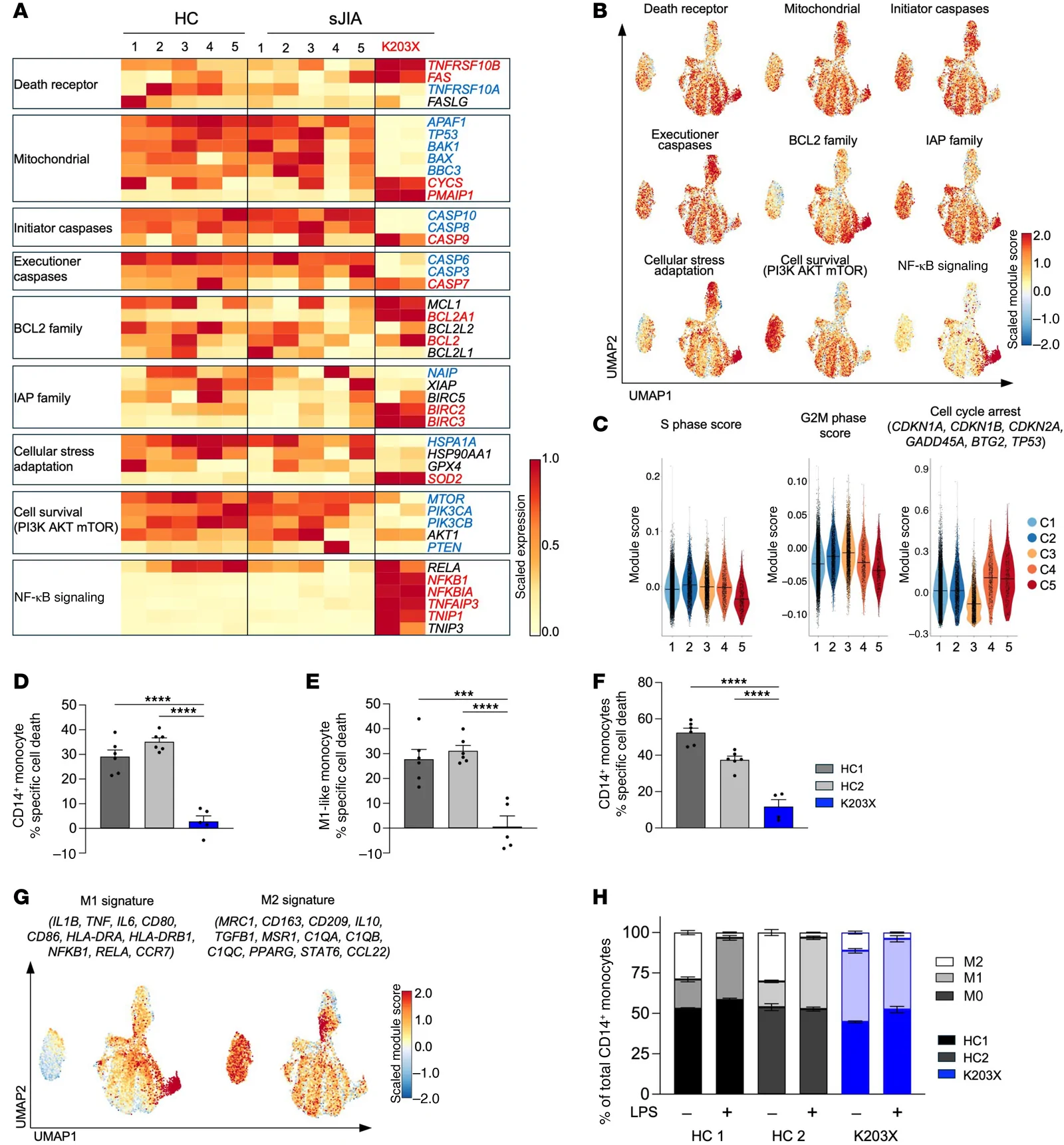
TNFR1 K203X is associated with impaired monocyte cell death and maintenance of a proinflammatory phenotype. (**A**) Scaled expression of cell death- and survival-related genes in monocytes, grouped by functional modules. DEGs between the sJIA-K203X patient and healthy controls (HCs) are shown in red (upregulated) or blue (downregulated). (**B**) Feature plots showing module scores for 9 cell death and survival modules defined in **A**, overlayed on the monocyte UMAP from Figure 5**A**. (**C**) Violin plots showing S phase, G2/M phase, and cell cycle arrest module scores across monocyte clusters. Black dots represent individual cells and horizontal lines indicate cluster medians. (**D**) Frozen PBMCs from the K203X patient and healthy controls were thawed, rested for 60 minutes, and incubated with LPS (10 ng/ml). Cells were stained for cell surface markers, Annexin V, and Live/Dead Blue. Monocytes were assessed for specific cell death by flow cytometry. ***P < 0.001; ****P < 0.0001 comparing healthy controls versus K203X via ANOVA. (**E**) Specific cell death in M1-like monocytes (CD3^–^CD19^–^CD14^+^CD80^+^CD206^–^), assessed as in **D**. (**F**) Specific cell death following Fas ligand stimulation (FasL; 10 ng/ml), assessed as in **D**. **D**–**F** show cumulative data from *N* = 3 experiments. ***P < 0.001; ****P < 0.0001 comparing healthy controls versus K203X by ANOVA. (**G**) Feature plots displaying module scores for M1 and M2 monocyte gene sets in monocytes overlaid on the UMAP described in Figure 5**A**. (**H**) CD14^+^ monocytes were evaluated for M1 (CD80^+^CD206^–^) and M2 (CD80^–^CD206^+^) surface markers in healthy controls and K203X PBMCs in the presence or absence of LPS stimulation (10 ng/ml). Percentage of each population was calculated from the total CD14^+^ monocyte population. Data are cumulative of *N* = 3 experiments.

**Figure 9:**
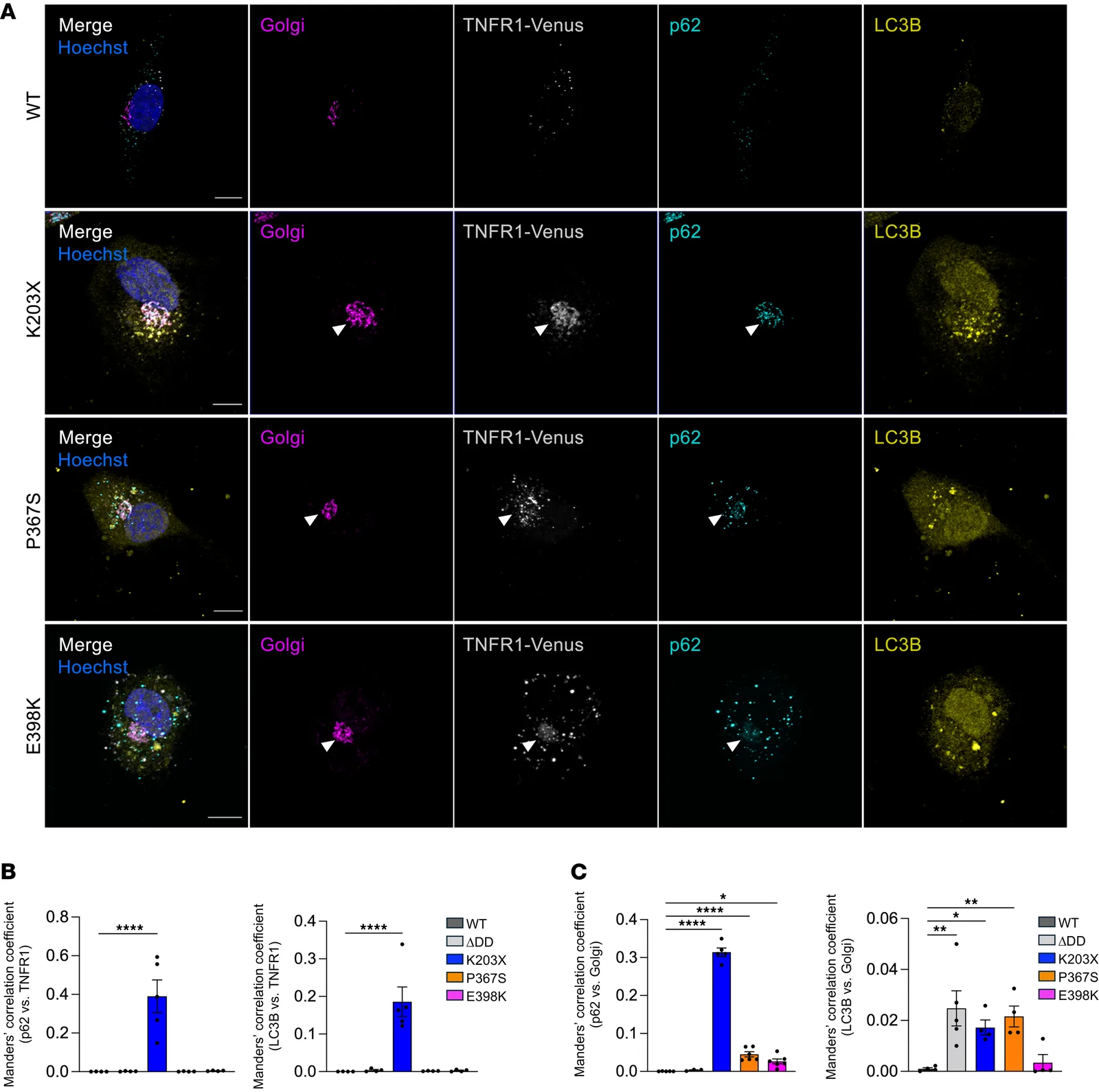
Variants of TNFR1 are associated with autophagy marker accumulation, Golgi localization, and cellular stress response. (**A**) Spatial localization of TNFR1 with markers of autophagy. HT1080 cells were transfected with Venus-tagged WT or variant TNFR1 as indicated. Cells were fixed and stained with antibodies to GM130, p62/SQSTM1, and LC3B. Cells were counterstained with Hoechst 33342 for nuclear detection and imaged via super-resolution confocal microscopy. Arrows show localization of TNFR1 with both the Golgi and p62. Images are representative of *N* ≥ 6 fields captured, and *N* = 3 independent experiments. Scale bar: 10μm. (**B**) Manders’ correlation coefficient of p62 or LC3B with TNFR1, with *N* ≥ 4 representative cells from each variant used for calculation. ****P < 0.0001 via ANOVA. (**C**) Manders’ correlation coefficient of p62 or LC3B with Golgi. *N* ≥ 4 representative cells from each variant were used for calculation. *P < 0.05; **P < 0.01; ****P < 0.0001 by ANOVA.

**Figure 10:**
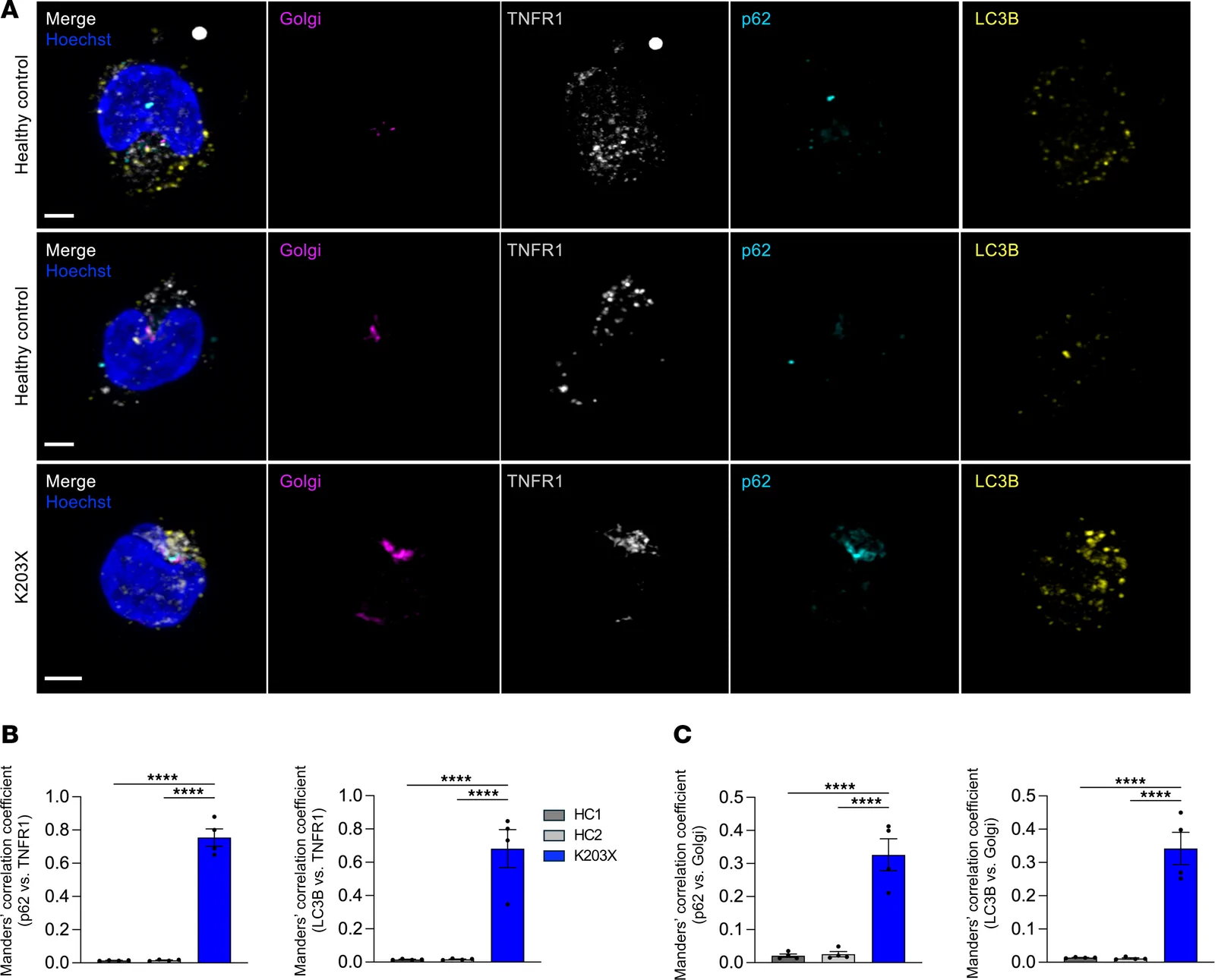
TNFR1 localization with autophagy markers in primary PBMCs from a patient harboring the K203X variant. (**A**) Representative super-resolution confocal images of PBMCs from the patient carrying the K203X variant and 2 age-matched healthy controls. Cells were fixed and stained with antibodies against GM130, p62/SQSTM1, LC3B, and TNFR1. Hoechst 33342 was used to visualize nuclei. Images are representative of at least 5 fields captured. Scale bar: 10μm. (**B**) Manders’ correlation coefficients showing proportion of p62 or LC3B signal overlapping with TNFR1. At least 4 representative cells from each individual were used for quantification. ****P < 0.0001 via 1-way ANOVA. (**C**) Manders’ correlation coefficients showing proportion of p62 or LC3B signal overlapping with the Golgi marker GM130. At least 4 representative cells from each individual were used for quantification. ****P < 0.0001 via 1-way ANOVA.

## Version 1 11/16/2026

Electronic publication
