# Supplementary material for: Golgi-retained TNFR1 variants induce stress-driven inflammation in a subset of systemic juvenile idiopathic arthritis
Source: J Clin Invest. 2026 Nov 16;136(22):e196975. doi: 10.1172/JCI196975 (PMC13767611; doi:10.1172/JCI196975)
Supplement: Supplementary file 1 [file jci-136-196975-s197.pdf]

**Supplementary Material**

**Supplementary Methods**

*Patient Samples*

All healthy control donors and Still's disease patients were enrolled in clinical trials at the NIH Clinical Center. Studies were approved by the NIH institutional review board. Written informed consent was obtained from all participants. DNA samples from the INCHARGE Consortium were included under a non-human subjects research exemption.

*Fluorescent resonance energy transfer*

FRET analysis was performed as previously described(61-63). Human TNFR1 or TNFR1 receptor lacking the death domain (ΔDD) were cloned into pEYFP-N1 (Clontech) engineered to express either mCerulean3 or mVenus fusion proteins. Patient-associated variants were introduced to WT TNFR1 constructs via site-directed mutagenesis. Paired Cerulean3 and Venus-expressing constructs were cotransfected into HEK293T cells (ATCC) using FuGENE HD (Promega). Evaluation of cells was performed 40hr post-transfection via flow cytometric detection of FRET on a LSR II SORP Fortessa flow cytometer (Becton Dickinson) with 447/488nm dual laser excitation. FRET data are shown for viable cells gated to equivalent fluorescent expression of Cerulean3 and Venus fusion proteins. Data were analyzed using FlowJo software v10.10.1 (Becton Dickinson).

*TNFR1 cloning and mutagenesis*

TNFR1 receptor cloning and mutagenesis was performed as previously described(64). Briefly, full-length human TNFR1 (a.a. 1-426) or TNFR1 lacking the death domain (a.a. 1-211; ΔDD) was cloned into pEYFP-N1 (Clontech), modified to express an N-terminal hemagglutinin (HA) tag and utilize the TNFR2 leader sequence to support efficient cell-surface expression. Site-directed mutagenesis was used to introduce patient-associated TNFR1 variants into the death domain (Pro367->Ser (P367S)); Glu398->Lys (E398K)) or a frameshift causing premature termination of the protein at Lys203 (K203term or K203X). All constructs were confirmed by Sanger sequencing.

*Immunoblotting and immunoprecipitation*

HT1080 cells (ATCC) were transfected with constructs harboring either WT or variant TNFR1 receptor via FuGENE HD (Promega). 16hr post-transfection, cells were incubated with 1 μg/ml TNF (Peprotech) for 90min, 37C, prior to harvest. Cells were lysed in IP lysis buffer (50mM Tris HCl, pH 7.4, 150mM NaCl, 1mM EDTA, 10% v/v glycerol, 5mM iodoacetic acid, 1% Triton X-100) supplemented with protease inhibitor cocktail (Roche). Lysates were cleared and protein concentration was determined using BCA Protein Assay Kit (Pierce) according to the manufacturer's protocol. Lysates were immunoprecipitated for 18hr, 4C, using magnetic beads coupled with anti-HA antibodies (Pierce). Protein was released from beads via boiling in 2x Laemmli loading buffer (Bio-Rad) supplemented with dithioltreitol (DTT; SigmaAldrich).

For immunoblot analysis, samples were loaded on to a 4-12% BisTris PAGE gel (Invitrogen) and blotted to PVDF. Blots were incubated with antibodies to TNFR1 (clone H5, 1:1000; Santa Cruz Biotechnologies (SCBT)), TRAF2 (clone F2, 1:500; SCBT), RIP (clone D94C12, 1:500; Cell Signaling), or TRADD (clone 37/TRADD, 1:1000; BD Transduction Labs). Blots were subsequently incubated with Veriblot secondary antibody detection reagent or Rat anti-mouse IgG conjugated to HRP (both 1:10000; Abcam) and developed using SuperSignal West Dura substrate (Pierce). Densitometry was performed on all blots using ImageLab software (Bio-Rad Labs), with quantitation derived using the WT without stimulation as baseline, normalized to the relative TNFR1 levels captured via immunoprecipitation of each receptor.

For autophagy blots, HT1080 cells were transfected as above followed by treatment with or without 10nM bafilomycin (Baf, Invivogen) for 24h prior to lysis in radioimmunoprecipitation assay buffer (RIPA; Abcam), clearing by centrifugation and quantification by BCA as above. 20µg of protein was loaded in each well and run as described before transfer to PVDF and incubation with primary antibodies against LC3A/B (4108; 1:1000; Cell Signaling) or β-actin (clone C4; 1:1000; SCBT) and subsequent secondary antibodies against mouse or rabbit IgG as appropriate (7076, 7074; 1:1000; Cell Signaling). Autophagy blots were performed in 3 independent experiments with transfections and treatments performed on different days. Autophagic flux was calculated as the difference in background-adjusted LC3A/B signal between bafilomycin-treated and untreated cells, normalized to β-actin.

For UPR immunoblots, Jurkat 3T8 cells were transfected via electroporation (BTX ECM 830, Harvard Apparatus) followed by treatment with or without 10ng/ml TNF (Peprotech) for 24hr. Lysates were prepared as above, and probed for ATF-4 (Clone D4B8; 1:1000; Cell Signaling), p-P38 (Clone D3F9; 1:1000, Cell Signaling), total P38 (#9212; 1:1000; Cell Signaling), or β-actin (Clone C4; 1:1000; SCBT). Densitometry was performed on N=3 replicates via ImageLab (Bio-Rad) with all transfections and experiments performed on different days.

*Cytokine and Soluble TNFR1 measurement and analysis*

Frozen peripheral blood mononuclear cells (PBMCs) containing the TNFR1 K203X truncation and age-matched healthy controls were treated in the presence of LPS O111.B4 (10ng/ml) for 24hr, 37C. Conditioned media was harvested and assayed for cytokine levels using the LEGENDplex 13-plex Human Inflammation Panel 1 (BioLegend). Samples were analyzed on a FACSymphony A5 SORP (BD) according to the manufacturer's instructions and processed using the LEGENDplex Data Analysis Software Suite (BioLegend). Data across samples were normalized to the absolute number of monocytes in each individual and displayed as $pg*ml^{-1}$ per $1x10^4$ cells. For control subjects, technical replicates were averaged to determine individual-level mean levels. For the K203X case, technical replicates were averaged separately for the two sampling occasions and then the case-level mean was determined by averaging the two sampling-occasion means. The case-level mean was compared with the control distribution using the Crawford–Howell modified single-case t-test.

For measurement of soluble TNFR1, serum from sJIA patients (N=62) or healthy controls (N=30) were collected during routine clinical assessments. Serum was assayed for soluble TNFR1 using the DuoLink sTNFR1 ELISA kit (R&D Systems) according to the manufacturer's protocol and shown as absolute values of $pg*ml^{-1}$.

*Quantification of cell death*

Jurkat 3T8 cells were transfected with constructs harboring either Venus-tagged WT or variant TNFR1 receptor via electroporation (BTX ECM 830, Harvard Apparatus). 16hr post-transfection, Venus-positive cells were analyzed for cell death using Annexin V and Live/Dead (Molecular Probes/ThermoFisher) and

analyzed on a LSR II SORP Fortessa as previously described(65). HT1080 cells were transfected with Venus-tagged TNFR1 constructs via FuGENE HD. 18hr post-transfection, cells were stained with DiIC(5) (ThermoFisher) and Live/Dead and analyzed as above.

PBMCs were isolated from whole blood of a patient harboring the TNFR1 K203X variant and age-matched healthy controls and frozen. Frozen PBMCs were thawed and allowed to rest for 60min, 37C in RPMI media supplemented with 10% fetal bovine serum prior to incubating with LPS (O111.B4 (Sigma Aldrich); 10ng/ml) or Fas ligand (Mega-FasL (AdipoGen), 10ng/ml) for 24hr at 37C. Cells were stained with surface antibodies (CD3, CD4, CD8, CD14, CD16, CD19, CD80, CD206 (see Methods Table 1 for antibody information)), Live/Dead, and Annexin V and analyzed on a LSR II SORP Fortessa. Specific cell death was calculated as [1-(% treated)/(% untreated)]*100. Data analysis was performed using FlowJo software v10.10.1.

*Luciferase*

HEK293T cells were plated in 96-well tissue culture plates and transfected with WT or variant TNFR1 using Lipofectamine3000 (Invitrogen) in conjunction with NFkB-luciferase reporter constructs expressing firefly (pGL4.32) or *Renilla* (pRL-CMV) luciferase (Promega). 24hr post-transfection, cells were lysed and luciferase was evaluated using the Dual-Glo Luciferase Assay System (Promega). Luminescence was measured using a Synergy H1 microplate reader (Biotek Agilent). Data was normalized to control *Renilla* activity and expressed as the ratio of firefly to *Renilla* luminescence.

*Flow cytometric analysis of NF-κB activity*

Jurkat 3T8 cells (a kind gift from Adrian Ting, Emory University, Atlanta, GA) were generated to contain a rat Thy1.1 and human CD14 gene under the control of NFkB enhancers(33). Cells were transfected with Venus-tagged WT or variant TNFR1 constructs by electroporation. 18 hours post-transfection, cells were left unstimulated or stimulated with TNF for 6 hours for assessment of NF-κB reporter activity. Cells were stained with LIVE/DEAD Blue and Annexin V for viability assessment, together with anti-rat Thy1.1 antibody for detection of NF-κB reporter activity by surface Thy1 expression (anti-rat CD90.1, clone OX-7; BioLegend) and analyzed by flow cytometry. Baseline NF-κB activity was quantified in unstimulated viable

Venus-positive cells by measuring rat Thy1.1 surface expression and was expressed relative to cells transfected with WT TNFR1. Data were analyzed using FlowJo software v10.10.1.

*TNF stimulation of PBMCs*

Cryopreserved PBMCs from a patient harboring the TNFR1 K203X variant or age-matched healthy controls were rapidly thawed at 37C and gradually diluted in warm X-VIVO medium (Lonza) containing DNase I (10U/ml; Millipore Sigma), followed by washing and resuspension in X-VIVO supplemented with DNase I (25U/ml). Cells were plated in V-bottom plate and rested for 3.5hr at 37C in 5% $CO_2$. After resting, cells were stained for surface markers in X-VIVO for 30min, 37C using antibodies against CD56, CD4, CD8, CD19, CD3, and CD11c (see Methods Table 1 for antibody information). Cells were incubated in the presence or absence of TNF (50ng/ml final concentration; BioLegend) for 10min, 37C. Stimulation was arrested by rapid centrifugation at 4C followed by fixation in Cytofix buffer (BD Biosciences) for 20min at room temperature (RT). After washing with Perm/Wash buffer (BD Biosciences), cells were permeabilized with ice-cold Phosflow Perm Buffer III (BD Biosciences) for 30min at −20C, washed again with Perm/Wash buffer, and stained intracellularly for phosphorylated NF-κB p65 (Ser529) for 60min, RT. Cells were washed in Perm/Wash buffer, resuspended in staining buffer, and acquired on a 5-laser Cytek Aurora spectral flow cytometer (Cytek Biosciences), recording the maximum possible number of events per sample. Flow cytometry data were analyzed using FlowJo software v10.10.1.

To assess inflammatory cytokine production via flow cytometry, PBMCs from a patient harboring the TNFR1 K203X variant or age-matched healthy controls were thawed and rested as above. Rested cells were incubated in the presence or absence of TNF (50ng/ml) and monensin (BioLegend) for 4hr, 37C. After stimulation, cells were washed and stained with a fixable viability dye (BD Horizon Fixable Viability Stain 780) for 15min, 4C, washed with staining buffer, followed by surface staining for 30min at 4C using antibodies against TNFR1, CD56, CD11c, CD4, CD8, CD19, and CD3 (see Methods Table 1 for all antibody information). Cells were washed with staining buffer, fixed and permeabilized using Cytofix/Cytoperm for 20min, RT, washed with Cytofix/Cytoperm buffer, and stained overnight at 4C with antibodies against IL-8 and IL-1β. Cells were washed in Cytofix/Cytoperm buffer, resuspended in staining buffer and acquired on a 5-laser Cytek Aurora spectral flow cytometer, as above.

*Super-resolution confocal microscopy*

Frozen PBMCs harboring TNFR1 K203X and age-matched healthy controls were thawed and rested for 60min. Cells were plated on m-Slide 8-well$^{high}$ Poly-L-Lysine coated chambered slides (Ibidi) and allowed to adhere prior to fixation in microscopy grade 1% PFA (Electron Microscopy Sciences). Cells were subsequently re-hydrated in PBS, blocked and permeabilized in blocking solution (0.04% saponin, 5% BSA, 1% normal goat serum (all from Sigma) in PBS) and stained with antibodies to mark the endoplasmic reticulum (anti-KDEL) the Golgi complex (anti-GM130), and a biotinylated antibody against TNFR1 (see Methods Table 1 for antibody information) all diluted in fresh blocking solution, overnight at 4C. Cells were subsequently washed in PBS supplemented with 0.04% saponin and treated for 2hrs with Streptavidin-AlexaFluor568. For analysis of markers of autophagy, cells were treated as above with antibodies against p62/SQSTM1 and LC3B in addition to a biotinylated antibody against TNFR1. In this context, Streptavidin conjugated to AlexaFluor568 or AlexaFluor488 was used as described. (see Methods Table 1 for antibody information).

HT1080 cells were plated on m-Slide 8-well$^{high}$ Poly-L-Lysine coated chambered slides (Ibidi) prior to transfection with Venus-tagged WT or variant TNFR1 using FuGENE HD. Cells were washed, fixed and subsequently blocked/permeabilized as described above before staining with anti-KDEL and anti-GM130 overnight at 4C. Cells were washed in PBS supplemented with 0.04% saponin and counterstained with Hoechst 33342 (Sigma).

All micrographs were captured on a Zeiss LSM980 Airyscan 2 confocal system using the LSMplus deconvolution modality to achieve sub-diffraction resolution and operated by the ZEN Blue 3.6 software. Acquisitions were performed using a Plan-Apochromat 40X/1.4NA (UV) Vis-IR oil immersion lens (Zeiss) with pinhole set to 1 Airy unit (AU) using solid-state 405nm, 488nm, 561nm, 594nm or 639nm laser lines in frame mode, and detection via Gallium Arsenide (GaAs) detectors.

*scRNA-sequencing and analysis*

Single-cell RNA-seq libraries were prepared using the Chromium Single Cell 3' Library & Gel Bead Kit v3.1 (10x Genomics, P/N 1000121). GEMs were generated by combining barcoded Single Cell 3' v3.1 Gel

Beads with 16000 freshly isolated PBMCs and partitioning oil onto Chromium Next GEM Chip G. GEM-RT was performed in a C1000 Touch Thermal cycler with 96-Deep Well Reaction Module (Bio-Rad; P/N 1851197): 53°C for 45 min, 85°C for 5 min; held at 4°C. Following retrotranscription, GEMs were broken, and the single-strand cDNA was purified with DynaBeads MyOne Silane Beads. cDNA was amplified using the C1000 Touch Thermal cycler with 96-Deep Well Reaction Module: 98°C for 3 min; cycled 12 times: 98°C for 15 s, 63°C for 20 s, and 72°C for 1 min; 72°C for 1 min; held at 4°C. Amplified cDNA product was purified with the SPRIselect Reagent Kit (0.6× SPRI). Indexed sequencing libraries were constructed using the reagents in the Chromium Single Cell 3' Library & Gel Bead Kit v2 (10x Genomics, PN-120237). The final libraries were diluted to 3 nM and sequenced on an Illumina platform using the following read length: 28bp for Read1, 8 bp for I7 Index, and 91 bp for Read2.

Gene expression GEX libraries were mapped to the 10x Genomics GRCh38 2024 human reference genome using the count command in 10X Genomics Cell Ranger 9.0.0. The resulting count matrices were processed using Seurat version 5.0.3 in the R statistical computing environment (v4.4.0). Previously published scRNA-seq data from five inactive sJIA patients and five healthy pediatric controls were obtained from Verweyen et al. (J Clin Invest., 2023) and integrated with our in-house dataset. In-house and published samples were merged to create a unified dataset for downstream analysis.

Subsequent analysis was performed using functions from the Seurat package (version 5.3.0) in R (version 4.2.3). Cells with <2,000 and >100,000 UMIs, <750 and >7,500 detected genes and >10% mitochondrial gene expression were removed from the dataset. DoubletFinder was used to identify and remove any likely doublets. These were typically less than 3% of each batch.

To refine the monocyte subset, lymphocytes or lymphocyte-monocyte doublets were manually excluded based on canonical marker expression (T cell: TRAC, CD3D, CD3E, CD3G, TRBC1, TRBC2; B cell: PAX5, MS4A1, CD79A; NK cell: NCAM1, KLRD1, KLRK1). Putative ambient RNA contamination ("soup" transcripts) was also manually removed (IGHA1, IGHA2, IGHG1, IGHG2, IGHG3, IGHG4, IGHD, IGHE, IGHM, IGHGP, JCHAIN, IGLC1, IGLC2, IGLC3, IGLC4, IGLC5, IGLC6, IGLC7, IGKC). Genes lacking annotation or not classified as protein-coding were excluded from downstream analyses. Additionally, Y chromosome–linked genes (DDX3Y, EIF1AY, RPS4Y1, KDM5D, UTY, ZFY, TMSB4Y, PRKY, USP9Y, TBL1Y, NLGN4Y) were removed to minimize potential sex-related confounding effects.

Principal component analysis (PCA) was performed on the 2,000 most variable genes to reduce dimensionality. Clustering was conducted using the Leiden algorithm as implemented in Seurat. The optimal clustering resolution was determined using the Clustree and based on cluster stability and biological interpretability. Clusters were annotated based on the expression of canonical marker genes representing major immune cell types.

Differentially expressed genes (DEGs) were identified using Seurat's FindMarkers function, with thresholds of average $\log_2$ fold change > 0.5, minimum percentage of expressing cells > 10% (min.pct = 0.1), and Benjamini-Hochberg–adjusted P < 0.1.

Gene Ontology (GO) enrichment analyses were performed using the clusterProfiler package, focusing on both Biological Process (BP) and Cellular Component (CC) categories. Visualization of enriched pathways was carried out using enrichplot and pathview (for KEGG pathway mapping). Module scores were calculated using the Seurat AddModuleScore function. For Figure 5E, predefined gene sets representing the IKK complex (CHUK, IKBKB, IKBKG), anti-apoptotic signaling (BCL2, BCL2L1, BCL2A1, MCL1, XIAP, BIRC2, BIRC3, BIRC5, TNFAIP3, CFLAR), cell adhesion (ICAM1, VCAM1, SELE, SELL, CD58), immune regulation (NFKBIA, NFKBIB, IRF1, STAT1, STAT5A, PTGS2), chemokines (CCL2, CCL3, CCL4, CCL5), and inflammatory cytokines (TNF, IL1B, IL6, CXCL8, IL12B) were used. For Figures 7F, 8C, and 8G, module scores were calculated using either predefined gene sets, as indicated, or custom gene sets listed in the corresponding figure where applicable. Additional data visualization and figure generation were performed using ComplexHeatmap, ggVennDiagram, ggplot2, and ggpubr.

Methods Table 1: Flow Cytometry and Immunofluorescence Antibodies

| Antibody target | Conjugate | Clone | Company | DIlution |
|---|---|---|---|---|
| CD3 | AlexaFluor700 | UCHT1 | ThermoFisher | 1:75 |
| CD3 | Vio Bright R720 | REA613 | Miltenyi Biotec | 1:167 (cytokine); 1:500 (phospho) |
| CD4 | PerCP-eFluor710 | RPA-T4 | ThermoFisher | 1:75 |

| | | | | |
|---|---|---|---|---|
| CD4 | Brilliant Violet 650 | RPA-T4 | BioLegend | 1:167 (cytokine); 1:250 (phospho) |
| CD8 | PE | RPA-T8 | ThermoFisher | 1:100 |
| CD8 | Brilliant Violet 750 | SK1 | BioLegend | 1:167 (cytokine); 1:500 (phospho) |
| CD11c | PE-Fire700 | Bu15 | BioLegend | 1:100 (cytokine); 1:250 (phospho) |
| CD14 | BV510 | M5E2 | BioLegend | 1:125 |
| CD16 | FITC | eBioCB16 (CB16) | ThermoFisher | 1:200 |
| CD19 | BV421 | HIB19 | BD | 1:400 |
| CD19 | Horizon RB613 | SJ25C1 | BD Biosciences | 1:167 (cytokine); 1:500 (phospho) |
| CD56 (NCAM) | APC | MEM-188 | BioLegend | 1:125 |
| CD56 (NCAM) | PE-Fire640 | QA17A16 | BioLegend | 1:50 |
| CD80 | PE-Dazzle594 | 2D10 | BioLegend | 1:50 |
| CD120a/TNFR1 | PE | W15099A | BioLegend | 1:25 |
| CD206 | PE-Cy7 | 15-2 | BioLegend | 1:50 |
| Live/Dead Blue | n/a | n/a | ThermoFisher | 1:1000 |
| BD Horizon Fixable Viability Stain 780 | n/a | n/a | BD Biosciences | 1:500 |
| Annexin V | APC | n/a | ThermoFisher | 1:250 |
| NF-κB p65 Phospho (Ser529) | PE | A21012B | BioLegend | 1:200 |

| | | | | |
|---|---|---|---|---|
| IL-8 | Brilliant Violet 510 | G265-8 | BD Biosciences | 1:20 |
| IL-1β | AlexaFluor647 | JK1B-1 | BioLegend | 1:100 |
| KDEL | AlexaFluor488 | EPR12668 | Abcam | 1:100 |
| KDEL | AlexaFluor594 | EPR12668 | Abcam | 1:100 |
| GM130 | AlexaFluor647 | EP89229Y | Abcam | 1:500 |
| GM130 | AlexaFluor594 | EP892Y | Abcam | 1:500 |
| TNFR1 | Biotin | H398 | ThermoFisher | 1:250 |
| p62/SQSTM1 | AlexaFluor555 | EPR4844 | Abcam | 1:100 |
| LC3B | AlexaFluor647 | ESQ2K | Cell Signaling | 1:200 |
| Streptavidin | AlexaFluor488 | n/a | ThermoFisher | 1:500 |
| Streptavidin | AlexaFluor568 | n/a | ThermoFisher | 1:500 |

Supplemental Figure 1

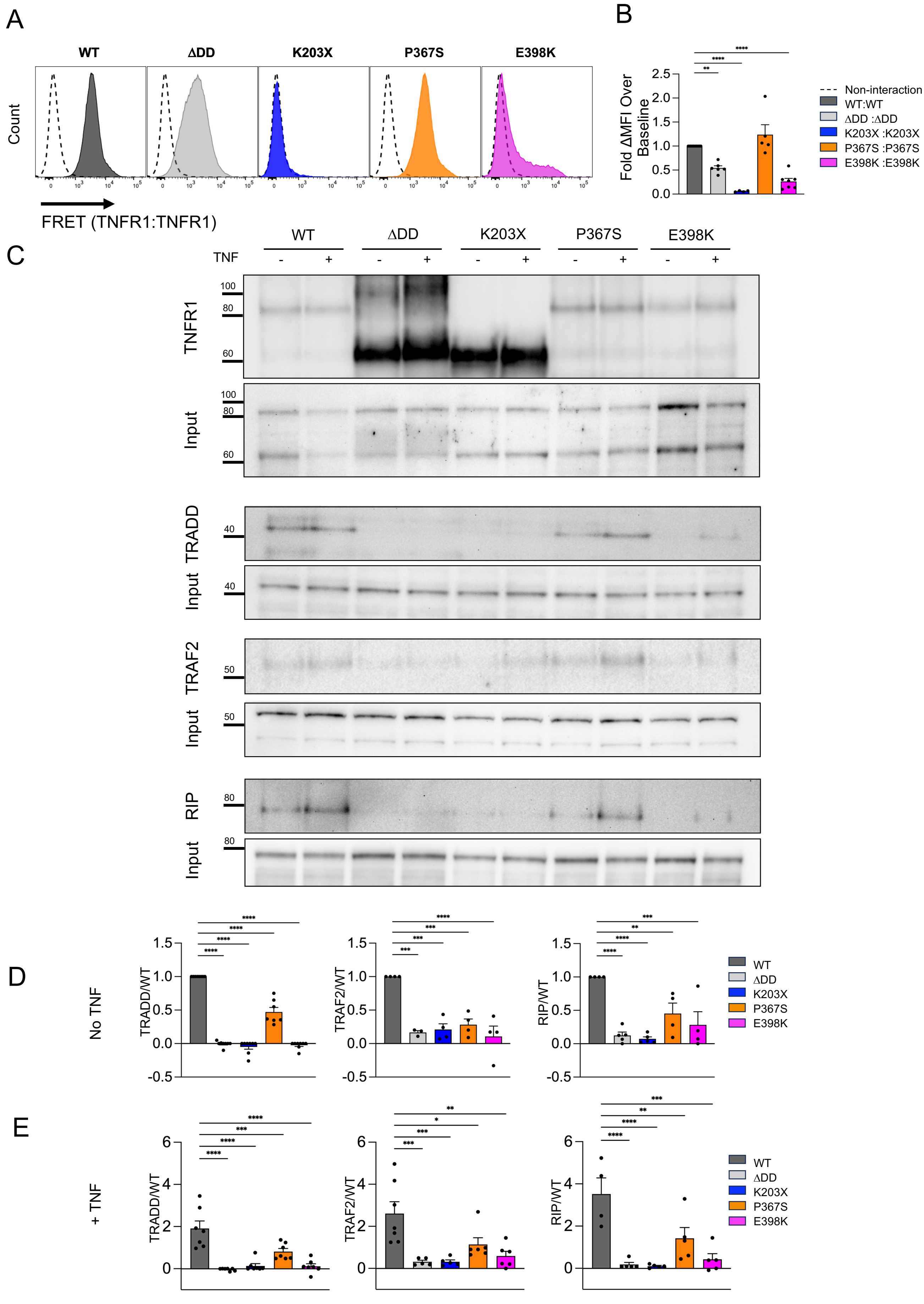

**Supplemental Figure 1: Mutant TNFR1 decreases recruitment of proximal signaling components upon receptor stimulation with TNF.**

**A.** Representative histograms depicting FRET analysis of homotypic interactions of WT and variant TNFR1 receptors in HEK293T cells. Cells were transfected with Cerulean3- or Venus-tagged TNFR1 constructs containing either WT or variant receptor and assayed 40hr post-transfection. For histograms, Black dashed: non-interaction control (TNFR1:Fas); Black: wild type (WT); Gray: ∆DD; Blue: K203X; Orange: P367S; Magenta: E398K.

**B.** Cells were analyzed for interactions by FRET-based flow cytometry, with fold change of MFI calculated as in Figure 2. N≥5 independent experiments for all conditions. ** $P < 0.01$; **** $P < 0.0001$ WT versus variants. ANOVA was used for statistical analysis. Black: wild type (WT); Gray: ∆DD; Blue: K203X; Orange: P367S; Magenta: E398K.

**C.** Representative Western blots of immunoprecipitated proteins analyzed in Figure **2C-D**. Cells were incubated 90min in the presence or absence of 1mg/ml TNF prior to immunoprecipitation using magnetic anti-HA beads. Immunoprecipitates were assessed by immunoblot for TNFR1, as well as components of the initial signaling complex (TRADD, TRAF2, RIP). 5% of total input for each condition is included. Blots are representative of minimum N=3 independent experiments.

**D, E.** HT1080 cells were transfected with WT or TNFR1 variants and proximal signaling complex formation was determined by immunoprecipitation and Western blot represented in panel **C**. Cells were incubated 90min in the absence (**D**) or presence (**E**) of 1mg/ml TNF prior to immunoprecipitation. Immunoprecipitates were assessed by immunoblot for TNFR1, as well as components of the initial signaling complex (TRADD, TRAF2, RIP). Densitometry was performed on blots at baseline (**D**) and upon TNF stimulation (**E**), using unstimulated WT TNFR1 as baseline to determine fold change in density for signaling components. Data are cumulative of minimum N=3 independent experiments. * $P < 0.05$; ** $P < 0.01$; *** $P < 0.001$; **** $P < 0.0001$ comparing unstimulated or stimulated conditions pairwise via ANOVA.

Supplemental Figure 2

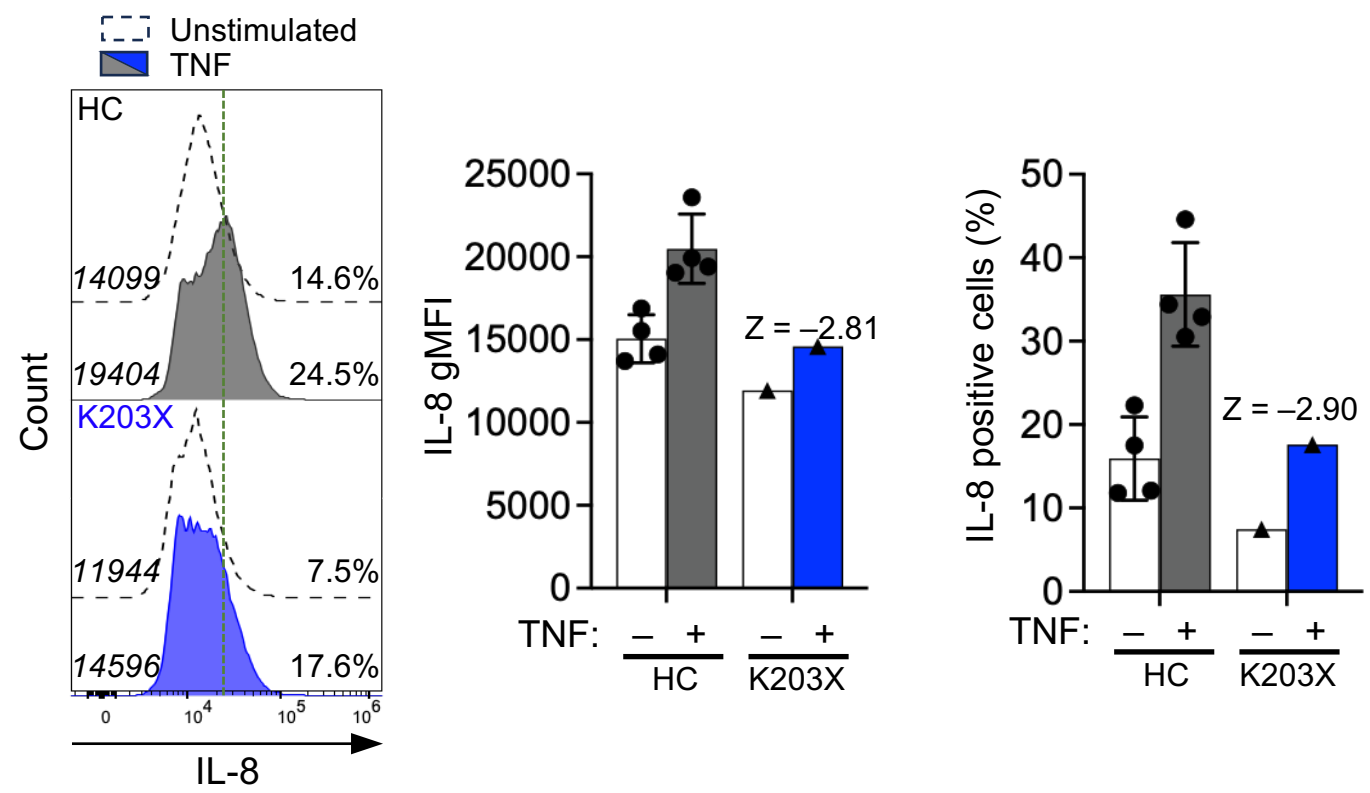


**Supplemental Figure 2. TNF-induced IL-8 production in PBMCs harboring the K203X TNFR1 variant**

PBMCs from healthy controls (HC, N=4) and a patient carrying the K203X mutation (N=1) were left unstimulated or stimulated with TNF and analyzed for intracellular IL-8 by flow cytometry. Histograms show unstimulated (dashed) and TNF-stimulated cells (filled; grey, HC; blue, K203X). Green dashed line indicates the positivity threshold. gMFI and percent positive cells are indicated. Summary plots show individual HC values with mean ± SD and the K203X patient value. Z-scores indicate deviation of K203X from TNF-stimulated HCs.

Supplemental Figure 3

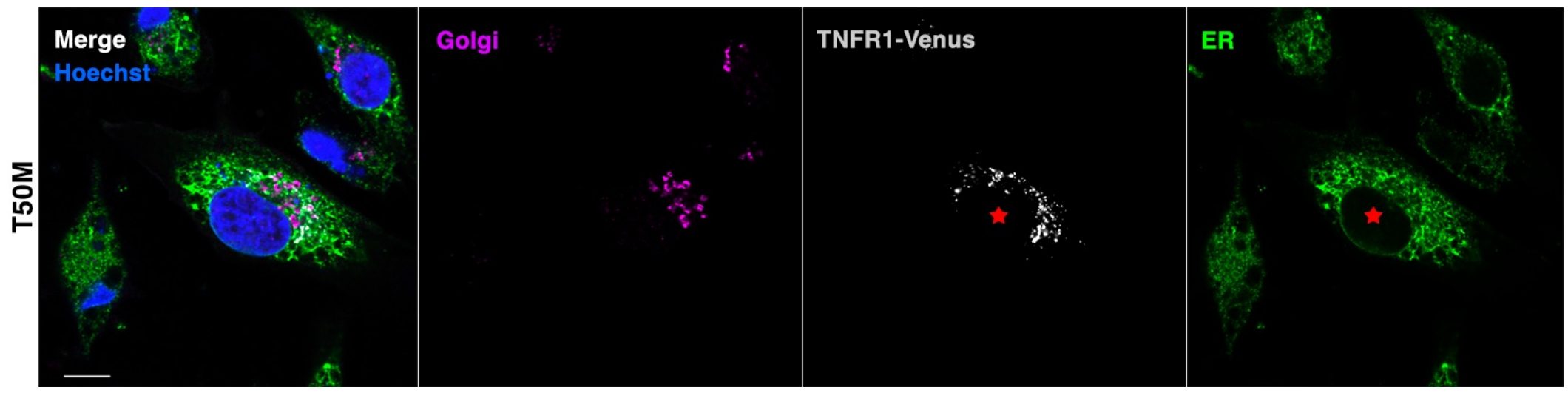


**Supplemental Figure 3. TNFR1-T50M is preferentially localized to the endoplasmic reticulum.** HT1080 cells were transfected with Venus-tagged WT or T50M TNFR1 and treated similar as in Figure 3 (TNFR1: gray; GM130: magenta; KDEL: green; nucleus: blue). Red star represents localization of TNFR1 with the ER. Images are representative of N≥6 fields captured, and N=3 independent experiments. Scale bar: 10μm.

Supplemental Figure 4

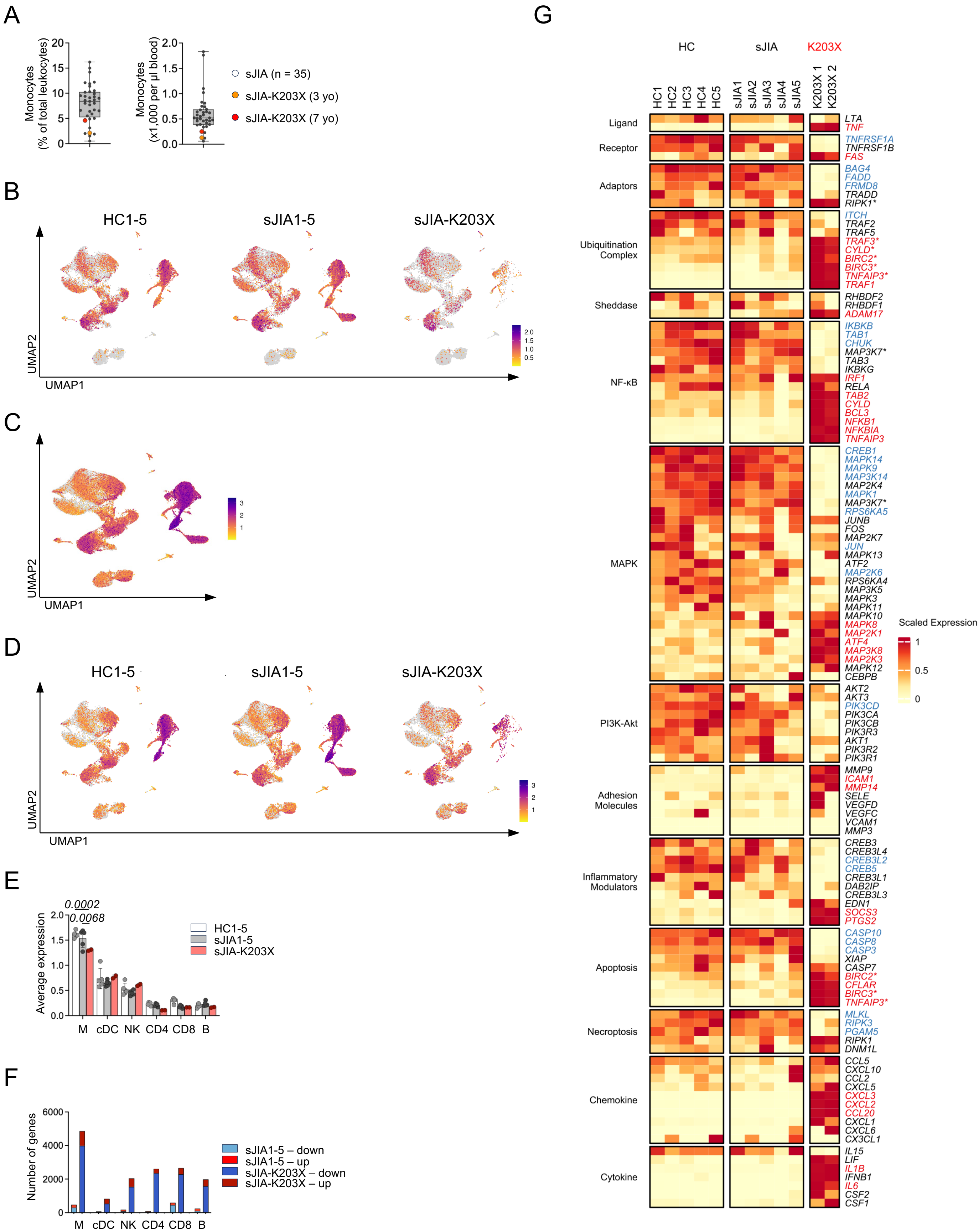

Supplemental Figure 4

H

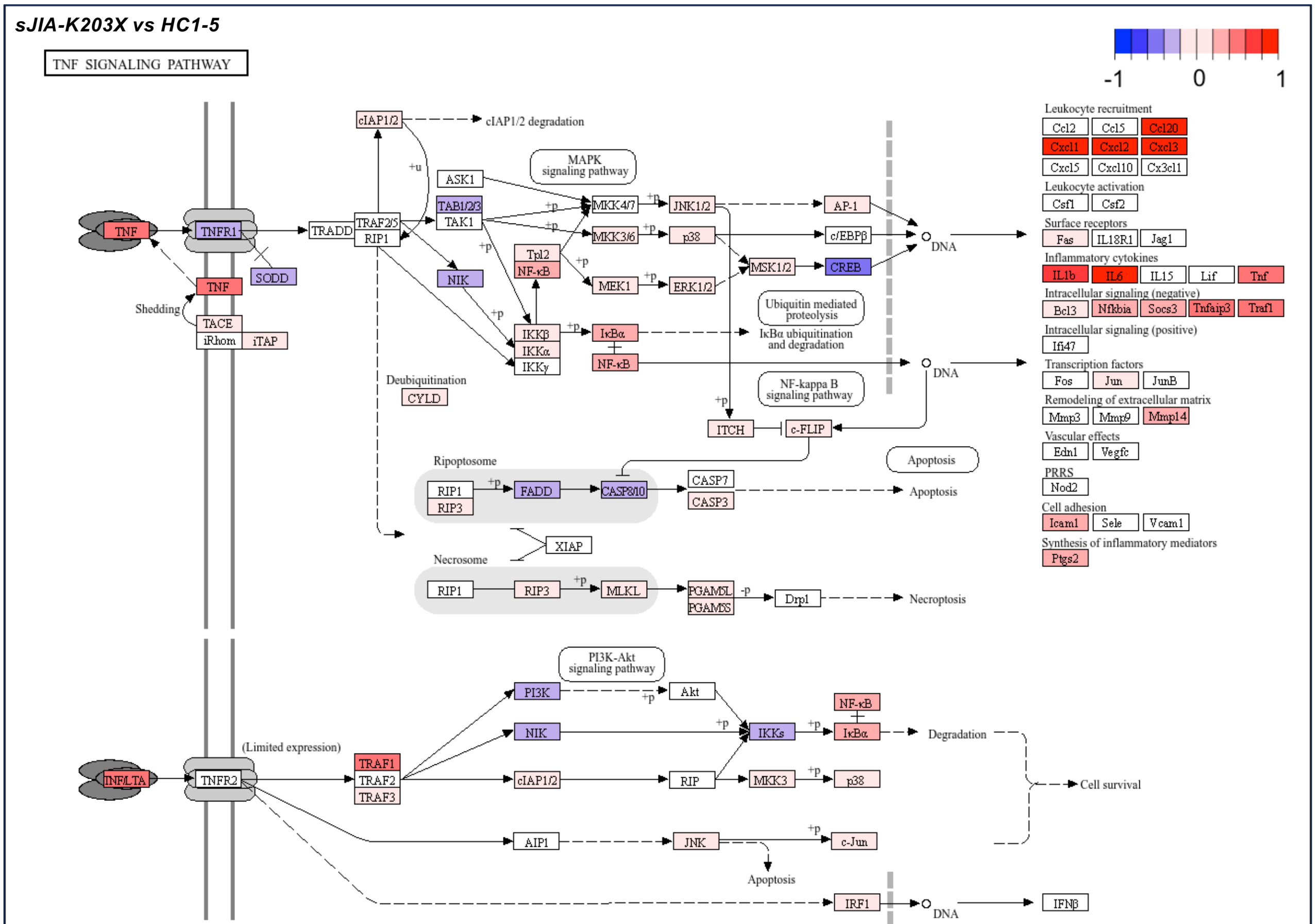


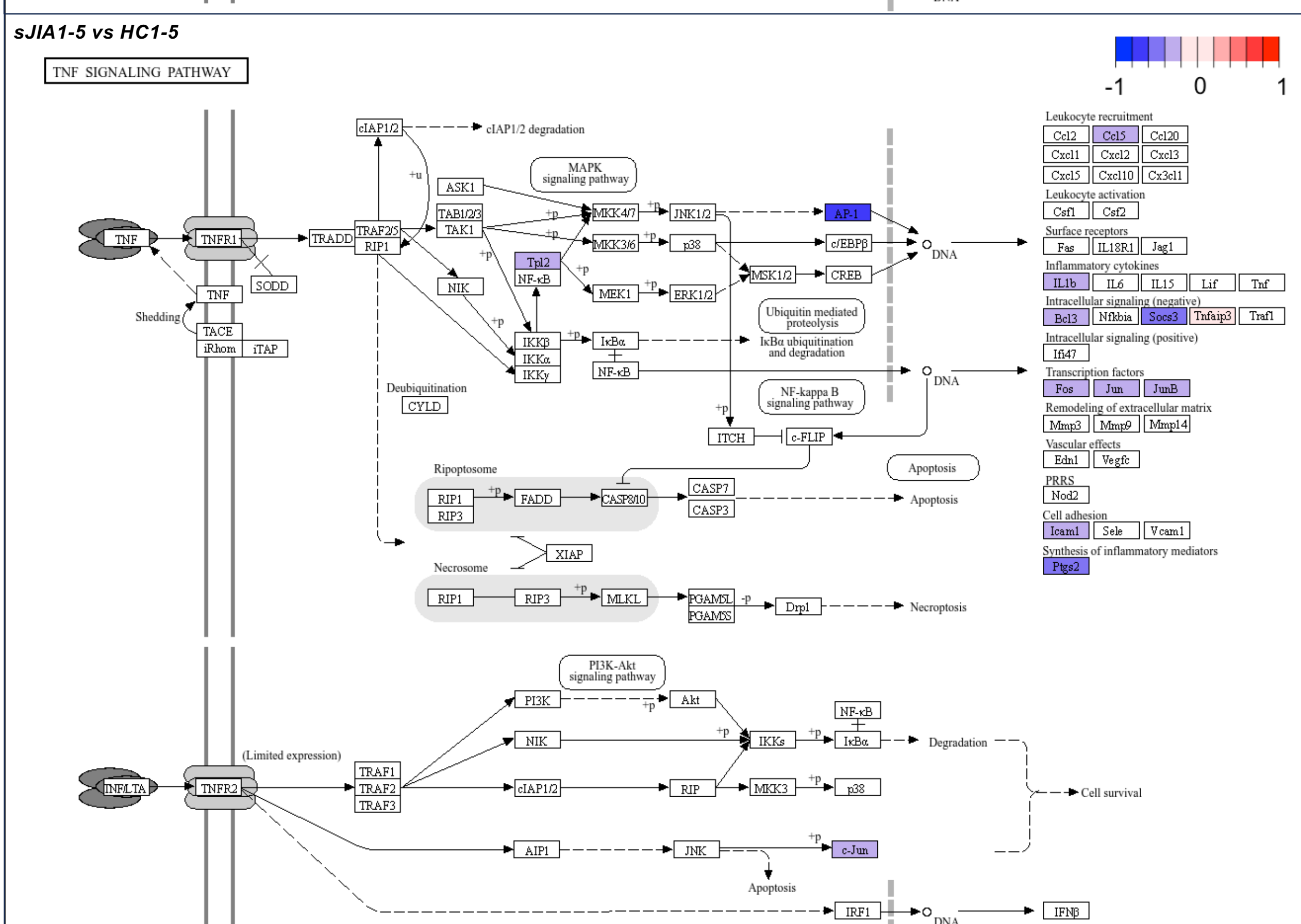

**Supplemental Figure 4. Monocyte abundance, TNF receptor expression across PBMC subsets, monocyte TNF signaling pathway gene expression and TNF signaling alterations in the sJIA-K203X patient.**

**A.** Proportion of monocytes in $CD45^{+}$ leukocytes (left panel) and absolute count of monocytes (right panel) in peripheral blood of sJIA patients. The sJIA-K203X patient is highlighted in yellow and orange at ages 3 and 7, respectively.

**B.** Feature plot showing the normalized expression of *TNFRSF1A* by groups (HC1-5, sJIA1-5 and sJIA-K203X) overlaid on the UMAP from Figure **5A**.

**C.** Feature plot showing the normalized expression of *TNFRSF1B*, overlaid on the UMAP from Figure **5A**.

**D.** Feature plot showing the normalized expression of *TNFRSF1B* by groups (HC1-5, sJIA1-5 and sJIA-K203X), overlaid on the UMAP from Figure **5A**.

**E.** Normalized expression of *TNFRSF1B* across major PBMC subsets by groups. Statistical comparisons were performed using two-way ANOVA with multiple comparison and the p-values are shown where statistically significant.

**F.** Number of upregulated and downregulated genes in main PBMC subsets in sJIA1-5 and sJIA-K203X compared to HC1-5.

**G.** Scaled expression of TNF signaling pathway (KEGG hsa04668) genes in monocytes, grouped by their position in the pathway. Differentially expressed genes (DEGs) between the sJIA6-K203X patient and healthy controls (HC) are shown in red (upregulated) or blue (downregulated). A star indicates genes that are present in multiple parts of the pathway. The sJIA-K203X patient is included in duplicate.

**H.** Visualization of the expression of TNF signaling pathway (KEGG hsa04668) genes in monocytes. DEGs between the sJIA-K203X patient and healthy controls (HC) (left panel) or the sJIA1-5 patients and healthy controls (HC) (right panel) are shown in red (upregulated) or blue (downregulated).

Supplemental Figure 5

A

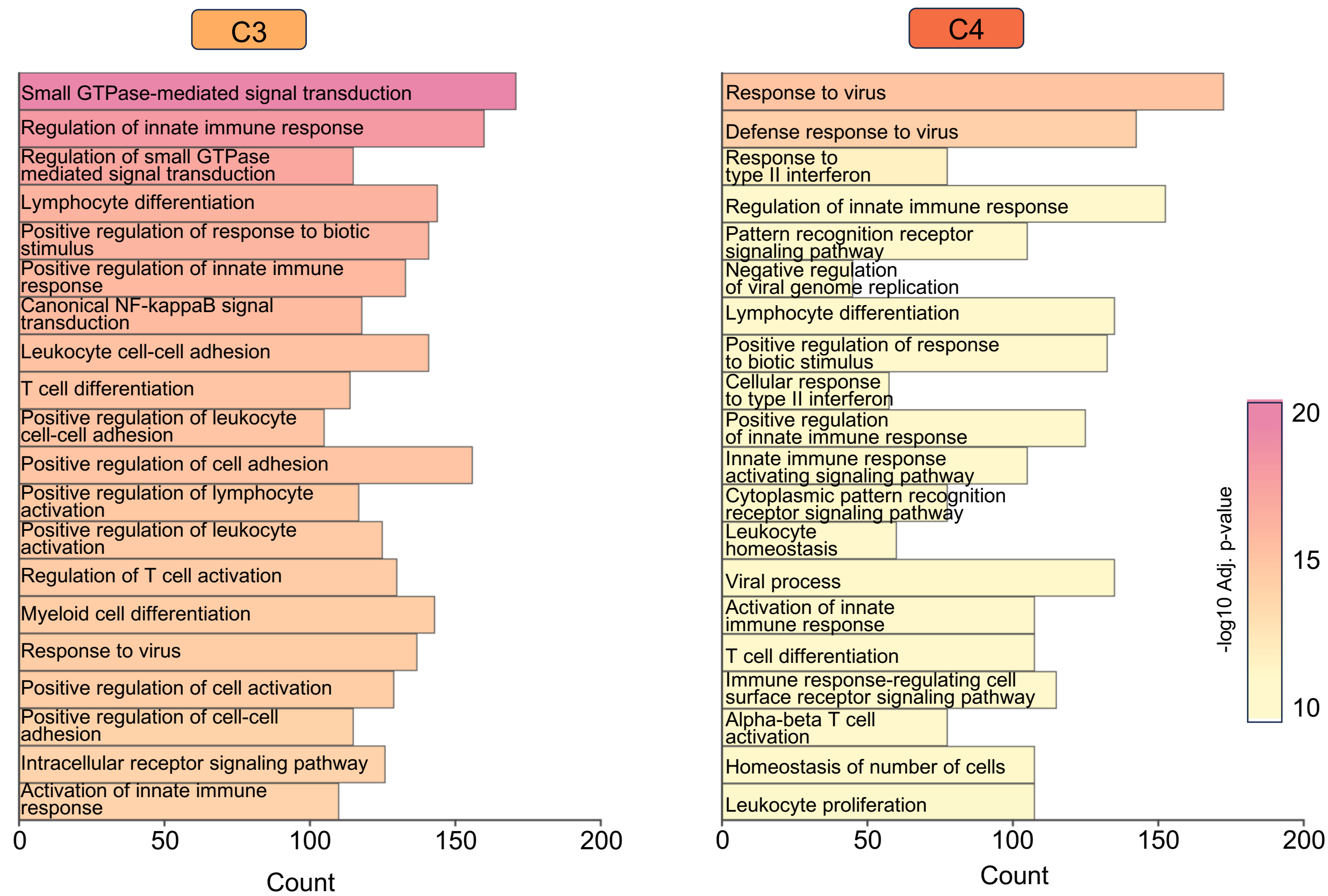


B

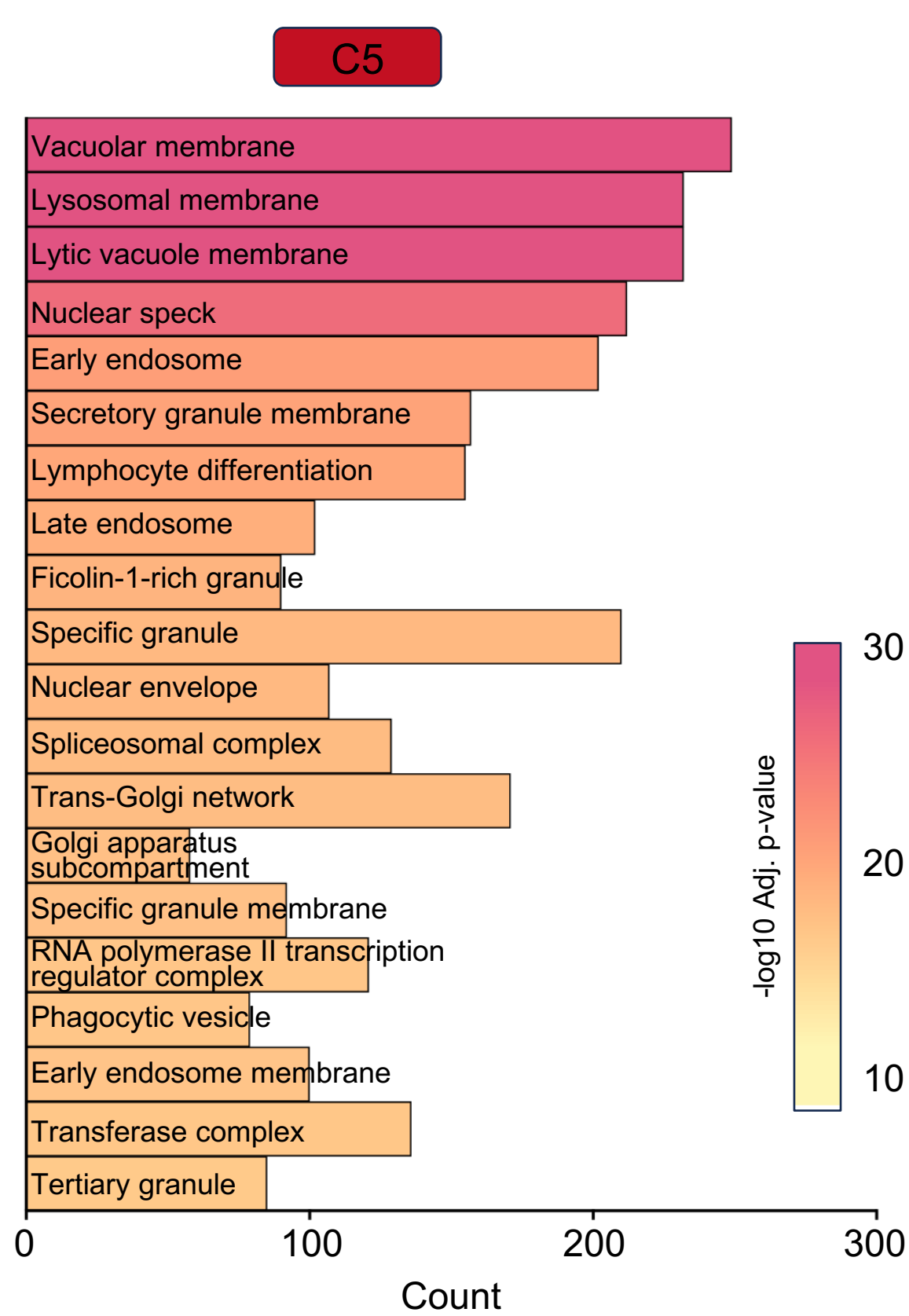

Supplemental Figure 5

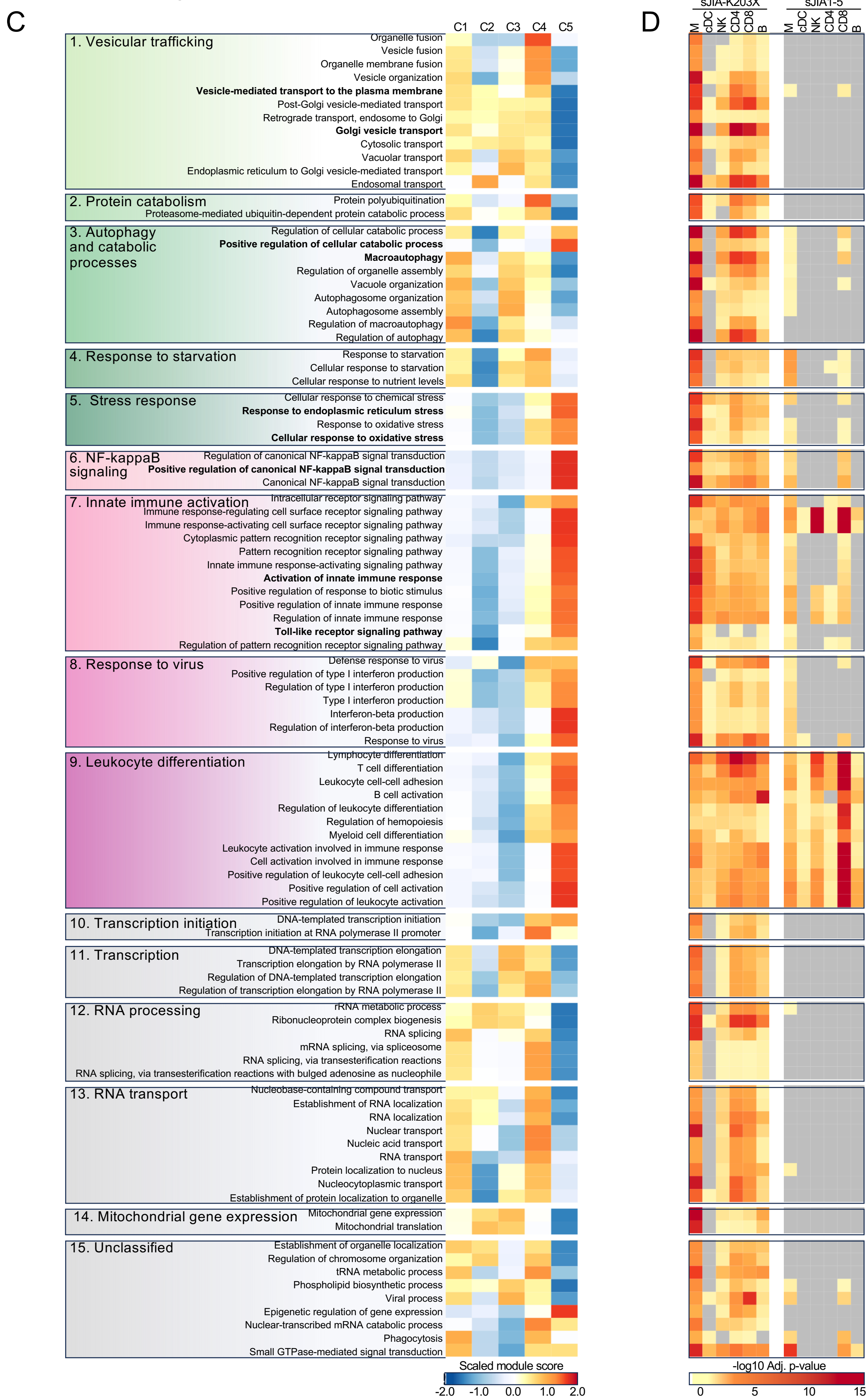

**Supplemental Figure 5. Transcriptional programs enriched in TNFR1-K203X monocytes extend across additional PBMC subsets.**

**A.** Bar plot showing the top twenty most significantly enriched GO BP terms from DEGs identified between the sJIA4-specific monocyte cluster (C3) and classical monocytes (C1) (left panel) and the sJIA5-specific monocyte cluster (C4) and classical monocytes (C1) (right panel). The x-axis shows the number of genes associated with each term (Count), while the color gradient reflects the adjusted p-value (p.adjust).

**B.** Bar plot showing the top twenty most significantly enriched GO cellular component (CC) terms from DEGs identified between the sJIA-K203X-specific monocyte cluster (C5) and classical monocytes (C1). The x-axis shows the number of genes associated with each term (Count), while the color gradient reflects the adjusted p-value (p.adjust).

**C.** Pathway module scores in monocyte clusters (C1–C5). Module scores for each indicated pathway were calculated based on DEGs associated with the given pathway, scaled between -2 and +2, and visualized as a heatmap.

**D.** Visualization of DEG enrichment across 96 pathways identified in C5 monocyte cluster, plotted as $-\log_{10}$-transformed adjusted p-values for each major PBMC subset in the sJIA-K203X patient and the unrelated sJIA patient cohort compared to healthy controls. The pathway list corresponds to panel **C**. Grey indicates pathways in which fewer than three DEGs were detected.

Supplemental Figure 6

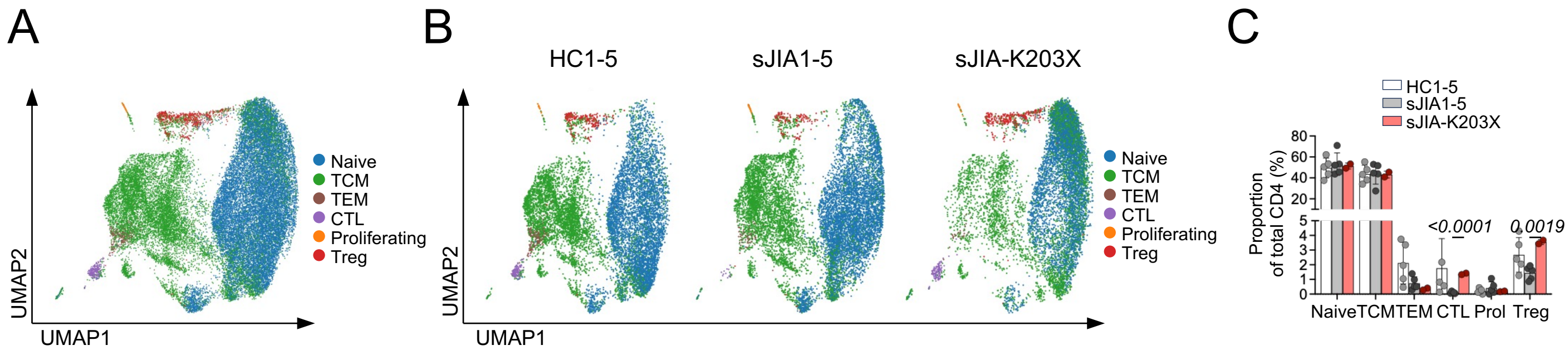


**Supplementary Figure 6. Subset composition of the CD4 T cell compartment.**

**A.** Clustering of CD4 T cells from 5 healthy controls and 6 sJIA patients visualized using Uniform Manifold Approximation and Projection (UMAP). The sJIA-K203X patient is shown separately for comparison, with data derived from two technical replicates of the sample collected at the second visit (age 7 years). (TCM: central memory $CD4^{+}$ T lymphocytes, TEM: effector memory $CD4^{+}$ T lymphocytes, CTL: cytotoxic $CD4^{+}$ T lymphocytes).

**B.** UMAP plot showing $CD4^{+}$ T lymphocyte distribution across clusters by subject group (healthy controls, sJIA patients, and the sJIA-K203X patient).

**C.** Distribution of $CD4^{+}$ T lymphocyte across clusters by sample.

# Supplemental Figure 7

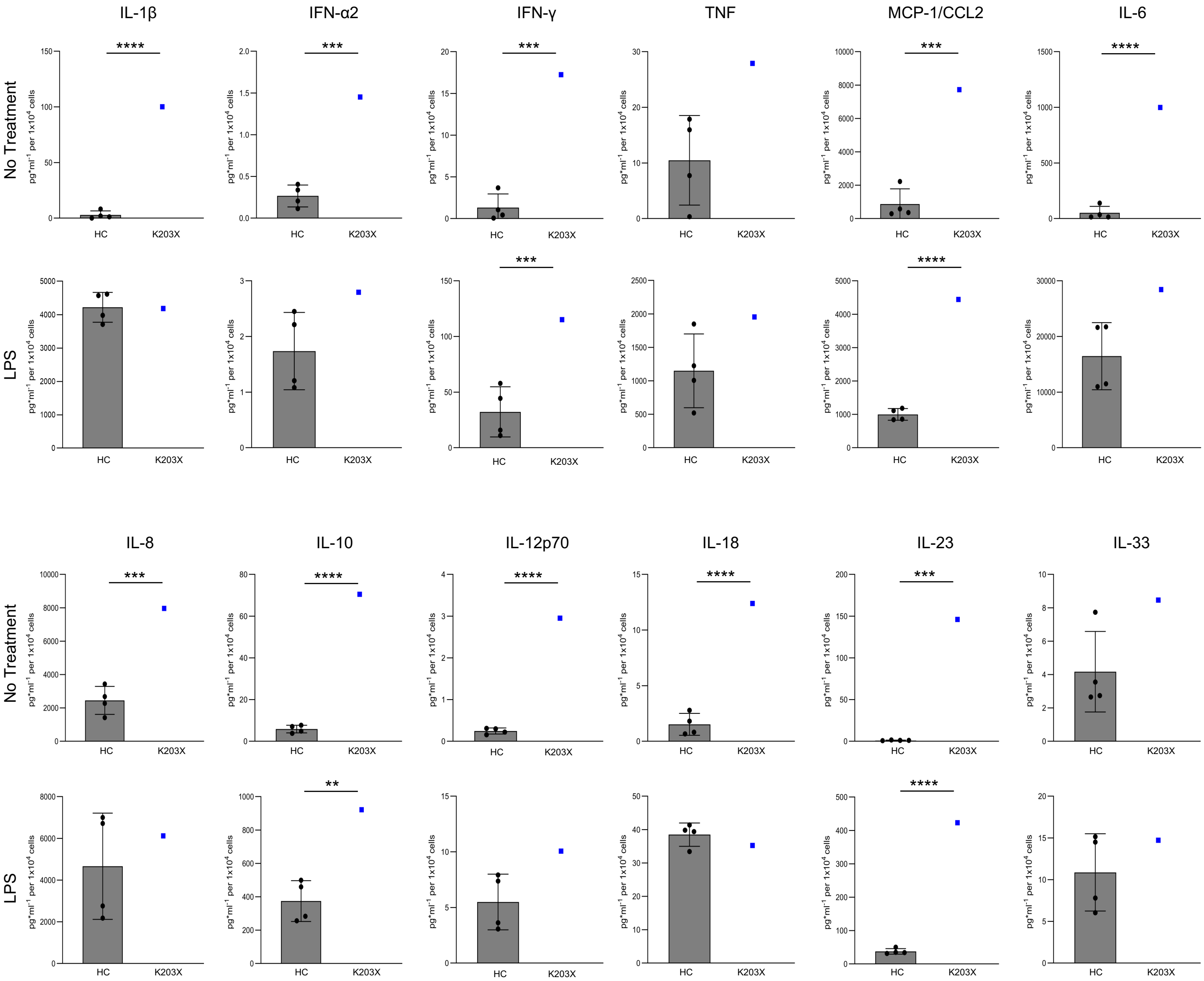


**Supplemental Figure 7. The K203X variant induces basal cytokine production.**
Frozen PBMCs from a patient harboring the K203X variant or healthy controls (HC) were thawed, rested and treated with LPS as in Figure **8.** Cell supernatants were collected and analyzed by a 13-plex cytokine bead array (CBA). Cytokine levels were normalized by absolute number of 1x$10^4$ monocytes. Each black circle represents the mean cytokine concentration for an independent control after averaging technical replicates (N=4), and the control group mean is shown as a grey bar. The blue square represents the case-level mean for the K203X patient, calculated by first averaging technical replicates (N=4) within the two sampling occasions and then averaging the two sampling-occasion means. The case-level mean was compared with the control group mean using the Crawford–Howell modified single-case t-test. * $P < 0.05$; ** $P < 0.01$; *** $P < 0.001$; **** $P < 0.0001$.

Supplemental Figure 8

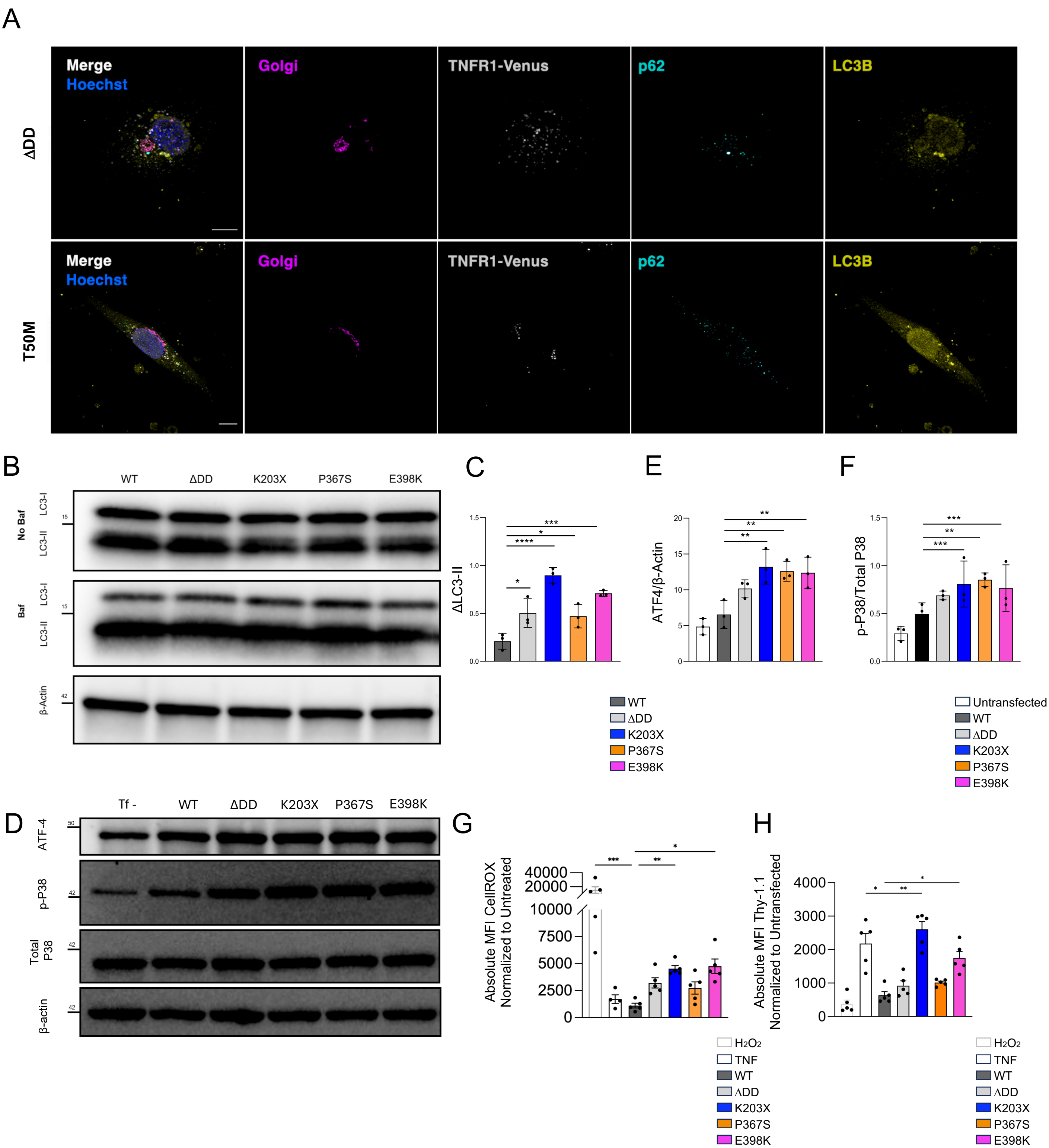

**Supplemental Figure 8: Overexpression of variants of TNFR1 induce increased autophagic processes.**

**A.** HT1080 cells were transfected with Venus-tagged TNFR1 lacking the Death Domain (∆DD) or T50M TNFR1 and treated similar as in Figure **9A** (TNFR1: gray; GM130: magenta; p62: cyan; LC3B: yellow; nucleus: blue). Images are representative of N≥6 fields captured, and N=3 independent experiments. Scale bar: 10μm.

**B.** HT1080 cells were transfected with WT TNFR1 or the indicated variants. 24hr post-transfection cells were treated with or without bafilomycin for 24hr, then lysed and analyzed by Western blot. Blots are representative of N=3 experiments transfected and analyzed on different days.

**C.** Densitometric quantification of LC3-II from immunoblots in HT1080 cells transfected with WT or variant TNFR1 constructs and treated with or without 10nM bafilomycin for 24 hours, performed in **B**. Autophagic flux was calculated from the difference between bafilomycin-treated and untreated conditions, normalized to β-actin. Three independent replicates were performed, with transfections and treatments completed on separate days. Statistical analysis was performed by one-way ANOVA, *P < 0.05; ** P < 0.01; **** P < 0.0001.

**D.** Western blots of basal unfolded protein response and NF-κB signaling. Jurkat 3T8 cells were transfected with WT TNFR1 or the indicated receptor variants. 24hr post-transfection cells were lysed and analyzed by Western blot. Immunoblots are representative of N=3 experiments transfected and analyzed on different days.

**E, F.** Densitometric quantification of UPR-associated proteins in Jurkat 3T8 cells transfected with WT or variant TNFR1 constructs from panel **D**. Twenty-four hours after transfection, cells were harvested and immunoblotted for ATF4 (**E**) or phospho-p38 MAPK and total p38 MAPK (**F**). Phospho-p38 signal was normalized to total p38. Statistical analysis was performed by 1-way ANOVA. *P < 0.05; **P < 0.01; ****P < 0.0001.

**G.** Flow cytometric quantification of oxidative stress in Jurkat 3T8 cells transfected with WT or variant TNFR1 constructs. Twenty-four hours after transfection, cells were incubated with CellROX reagent to assess cellular oxidative stress and analyzed by flow cytometry. Hydrogen peroxide was used as a positive control. Data are shown as absolute median fluorescence intensity normalized to untransfected cells. Data are from 5 independent experiments for all conditions. Statistical analysis was performed by 1-way ANOVA. *P < 0.05; **P < 0.01; ***P < 0.001, WT versus variants. Color coding: white, control; black, WT; gray, ΔDD; blue, K203X; orange, P367S; magenta, E398K.

**H.** Basal NF-κB activation in Jurkat 3T8 cells transfected with WT or variant TNFR1. Cells were transfected 24hr before staining for cell surface rat Thy-1.1, a surrogate for NF-κB activation (see Figure **2D**). Cells treated with 10ng/ml TNF were included as positive control. Data are presented as absolute MFI normalized to untransfected cells. N=5 independent experiments for all conditions. * P < 0.05; ** P < 0.01 WT versus variants. Statistical analysis was performed by ANOVA. White: control; Black: wild type (WT); Gray: ΔDD; Blue: K203X; Orange: P367S; Magenta: E398K.

Supplemental Table 1

| Feature | K203X | P367S | E398K |
| --- | --- | --- | --- |
| Disease pattern | Monocyclic | Polycyclic | Persistent |
| Age of onset | 3 years old | Unknown | Unknown |
| Sex | Female | Male | Female |
| HLA-DRB1*15 | No | No | No |
| History of MAS | No | Unknown | Unknown |
| History of drug reaction | No | Unknown | Unknown |
| History of sJIA-LD | No | Unknown | Unknown |
| CID off steroids | Yes | Unknown | Unknown |
| CID off treatment | Yes | Unknown | Unknown |
| Treatments | NSAIDs<br>Prednisone<br>Canakinumab | Unknown | Unknown |
| ILAR criteria | Yes | Yes | No |
| Fever | Yes | Yes | Yes |
| Evanescent, salmon rash | Yes | Yes | No |
| Arthritis | Yes | Yes | Yes |
| Hepatosplenomegaly | Yes | Yes | No |
| Lymphadenopathy | Yes | Yes | No |
| Serositis | No | No | No |
| Other | | | Uveitis |

**Supplemental Table 1: Clinical characteristics patients harboring variants in TNFR1.**
MAS, macrophage activation syndrome; sJIA-LD, systemic juvenile idiopathic arthritis-associated lung disease; CID, clinically inactive disease; NSAIDs, nonsteroidal anti-inflammatory drugs; ILAR, International League of Associations of Rheumatology.

Supplemental Table 2

| Sample | Disease status | Age | Sex | Time in CID | Treatment |
|---|---|---|---|---|---|
| Control_1 | Control | 14 | M | N/A | N/A |
| Control_2 | Control | 16 | F | N/A | N/A |
| Control_3 | Control | 13 | M | N/A | N/A |
| Control_4 | Control | 9 | F | N/A | N/A |
| Control_5 | Control | 9 | F | N/A | N/A |
| Inactive_1 | Inactive SJIA | 12 | F | 30 months | Tocilizumab |
| Inactive_2 | Inactive SJIA | 8 | M | 7 months | Canakinumab |
| Inactive_3 | Inactive SJIA | 6 | F | 2 months | Anakinra, Steroids |
| Inactive_4 | Inactive SJIA | 12 | F | 6 months | Anakinra |
| Inactive_5 | Inactive SJIA | 3 | M | 9 months | Tocilizumab |
| K203X | Inactive SJIA | 7 | F | 25 months | None |

**Supplemental Table 2: Clinical characteristics of subjects included in scRNAseq.**

## APPENDIX A: INCHARGE CONSORTIUM MEMBERS

Jean-Paul Achkar (Cleveland, OH), Marta E. Alarcón-Riquelme (Granada, Spain and Solna, Sweden), Jordi Anton (Barcelona, Spain), Victoria L. Arthur (Bethesda, MD), Elizabeth Baskin (Bethesda, MD), John F. Bohnsack (Salt Lake City, UT), Joanna Cobb (Manchester, UK), Erkan Demirkaya (Ankara, Turkey), Elisa Docampo (Liège, Belgium and Barcelona, Spain), Richard H. Duerr (Pittsburgh, PA), Xavier Estivill (Barcelona, Spain and Doha, Qatar), Dirk Foell (Münster, Germany), Marco Gattorno (Genoa, Italy), Alexei Grom (Cincinnati, OH), Ahmet Gül (Istanbul, Turkey), Maria Odete E. Hilario (São Paulo, Brazil), Norman T. Ilowite (Bronx, NY), Johannes-Peter Haas (Garmisch-Partenkirchen, Germany), Anne Hinks (Manchester, UK), M. Ilyas Kamboh (Pittsburgh, PA), Daniel L. Kastner (Bethesda, MD), Kenneth M. Kaufman (Cincinnati, OH), Leah C. Kottyan (Cincinnati, OH), Carl D. Langefeld (Winston-Salem, NC), Claudio Len (São Paulo, Brazil), Alberto Martini (Genoa, Italy), Elizabeth D. Mellins (Stanford, CA), Kirsten Minden (Berlin, Germany), Sheila Oliveira (Rio de Janeiro, Brazil), Michael J. Ombrello (Bethesda, MD), Seza Özen (Ankara, Turkey), Dalila Pinto (New York, NY), Sampath Prahalad (Atlanta, GA), Elaine F. Remmers (Bethesda, MD), Angela Rosen-Wolff (Dresden, Germany), Alan Rosenberg (Saskatoon, Canada), Ricardo Russo (Buenos Aires, Argentina), Stephen W. Scherer (Toronto, Canada), Sara Signa (Genoa, Italy), Emily Shuldiner (Bethesda, MD), Ann Marie Szymanski (Bethesda, MD), Ioanna Tachmazidou (Hinxton, UK), Klaus Tenbrock (Aachen, Germany), Susan Thompson (Cincinnati, OH), Wendy Thomson (Manchester, UK), Lucy R. Wedderburn (London, UK), Patricia Woo (London, UK), Rae S. M. Yeung (Toronto, Canada), Andrew S. Zeft (Cleveland, OH), Eleftheria Zeggini (Hinxton, UK), Biologically Based Outcome Predictors in JIA (BBOP) Group, British Society of Pediatric and Adolescent Rheumatology (BSPAR) Study Group, Childhood Arthritis Prospective Study (CAPS) Group, Inception Cohort of Newly Diagnosed Patients with Juvenile Idiopathic Arthritis (ICON-JIA), Randomized Placebo Phase Study of Rilonacept in sJIA (RAPPORT) Investigators, and the Sparks-Childhood Arthritis Response to Medication Study (CHARMS) Group.

## Supplementary References

61. Siegel RM, et al. Measurement of molecular interactions in living cells by fluorescence resonance energy transfer between variants of the green fluorescent protein. *Sci STKE.* 2000;2000(38):pl1.

62. Chan FK, et al. Fluorescence resonance energy transfer analysis of cell surface receptor interactions and signaling using spectral variants of the green fluorescent protein. *Cytometry.* 2001;44(4):361-8.

63. Cruz AC, et al. Fas/CD95 prevents autoimmunity independently of lipid raft localization and efficient apoptosis induction. *Nat Commun.* 2016;7:13895.

64. Lobito AA, et al. Abnormal disulfide-linked oligomerization results in ER retention and altered signaling by TNFR1 mutants in TNFR1-associated periodic fever syndrome (TRAPS). *Blood.* 2006;108(4):1320-7.

65. Ramaswamy M, et al. Specific elimination of effector memory CD4+ T cells due to enhanced Fas signaling complex formation and association with lipid raft microdomains. *Cell Death & Differentiation*. 2011;18(4):712-20.
