# Supplementary material for: Golgi-retained TNFR1 variants induce stress-driven inflammation in a subset of systemic juvenile idiopathic arthritis
Source: J Clin Invest. 2026 Nov 16;136(22):e196975. doi: 10.1172/JCI196975 (PMC13767611; doi:10.1172/JCI196975)

Full unedited Western Blots for Supplemental Figure 1
TNFR1 Immunoprecipitation
anti-TNFR1 Immunoblot

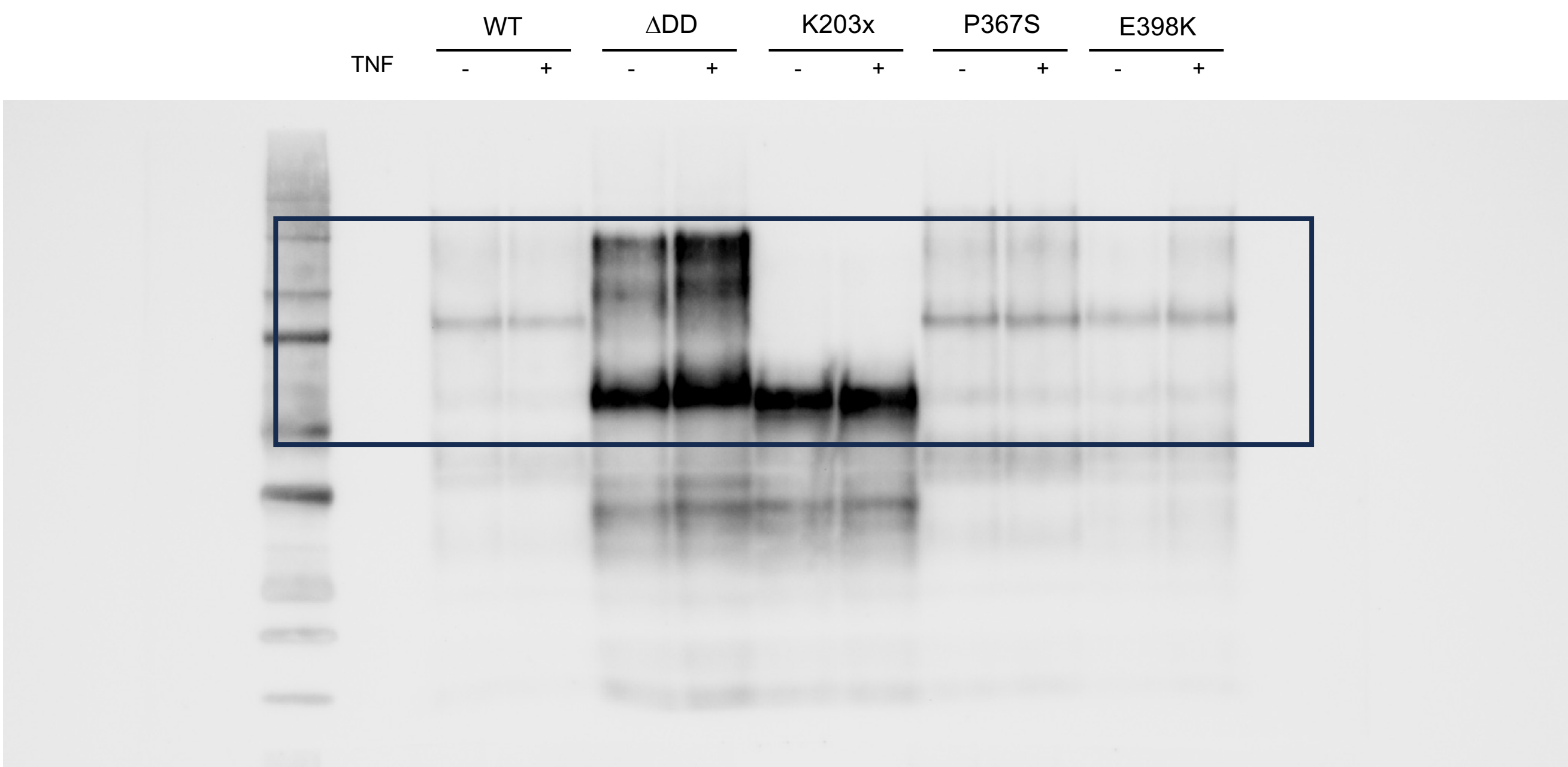

Full unedited Western Blots for Supplemental Figure 1
Whole cell lysate
anti-TNFR1 Immunoblot

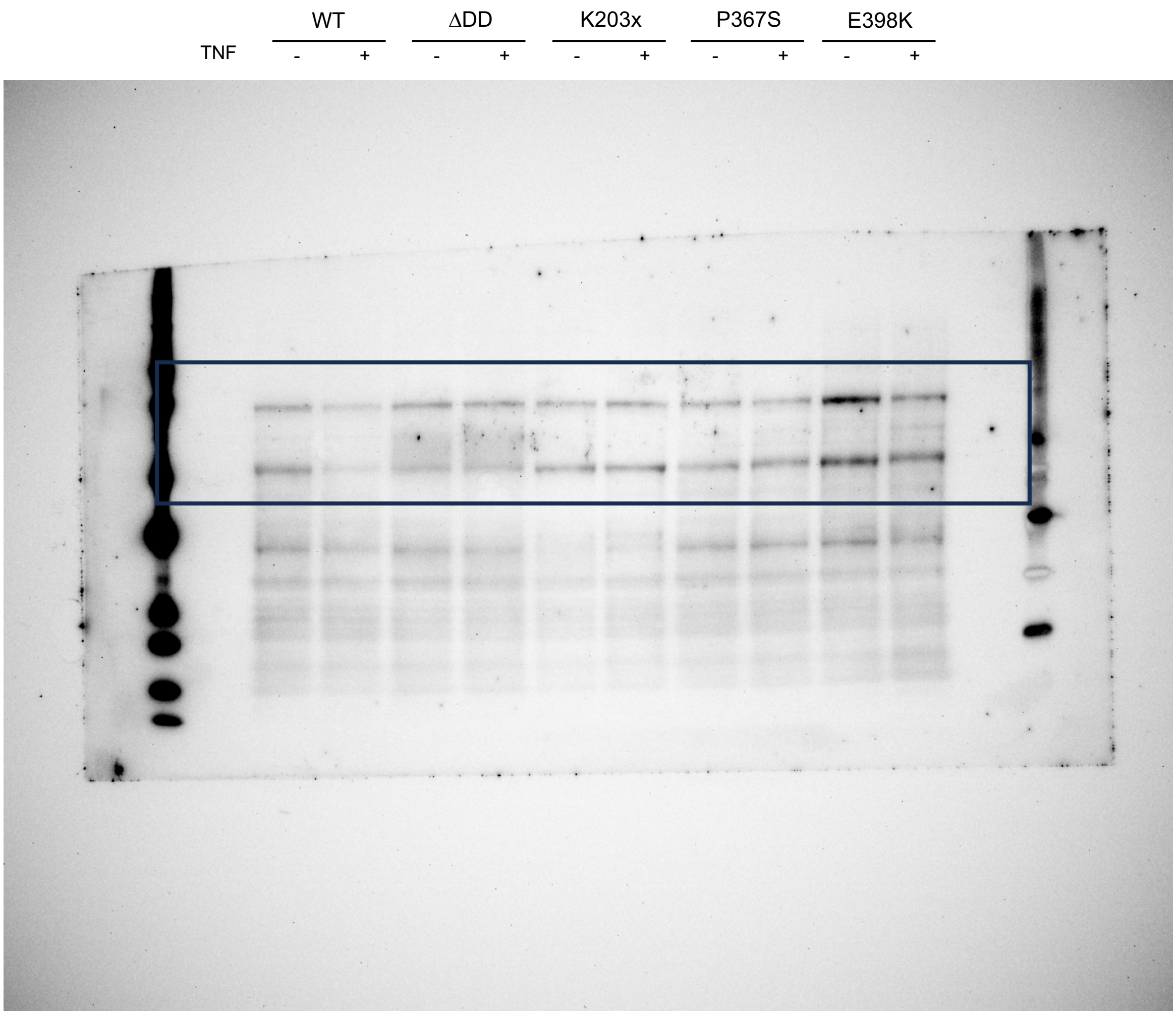

Full unedited Western Blots for Supplemental Figure 1
TNFR1 Immunoprecipitation
anti-TRADD Immunoblot

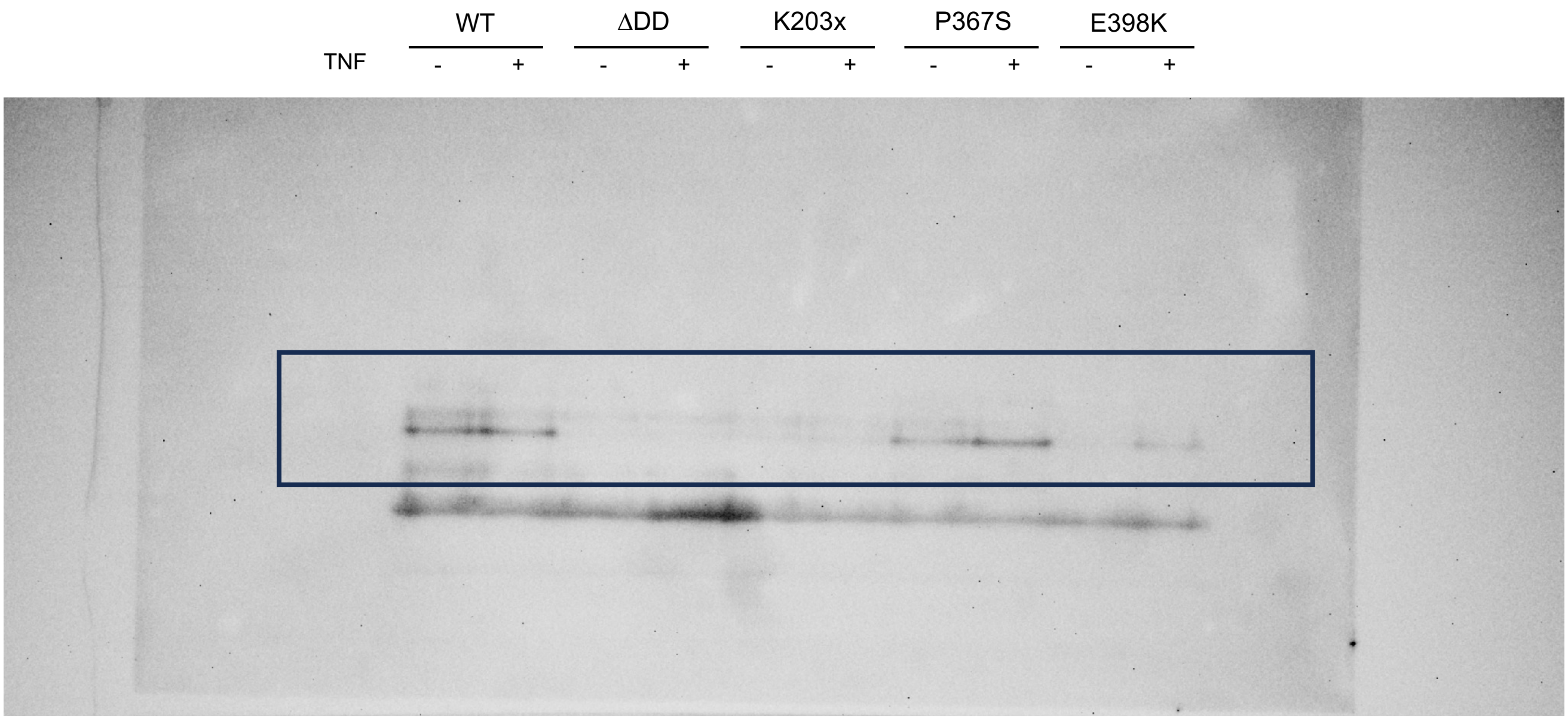

Full unedited Western Blots for Supplemental Figure 1
Whole Cell Lysate
anti-TRADD Immunoblot

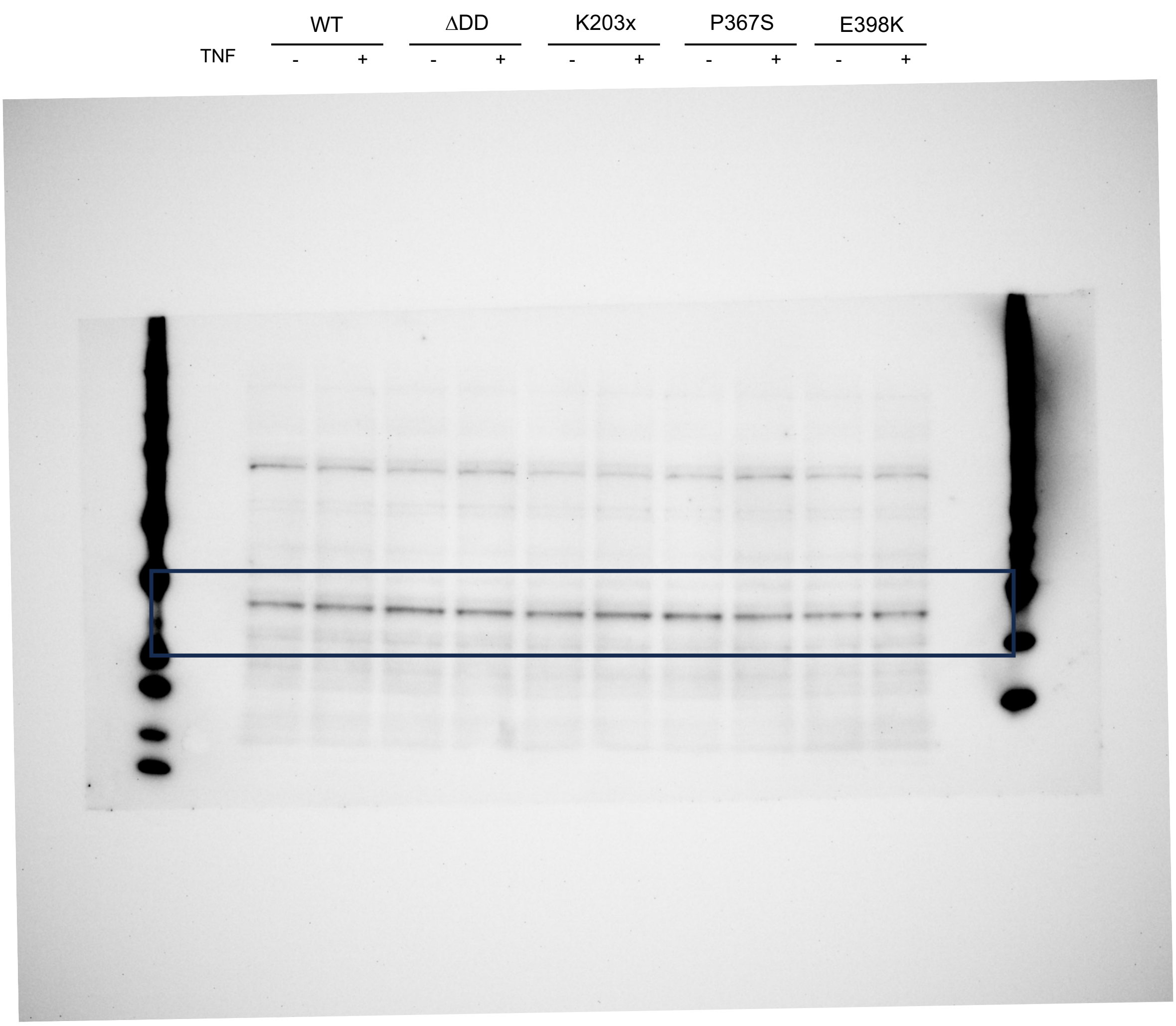

Full unedited Western Blots for Supplemental Figure 1
TNFR1 Immunoprecipitation
anti-TRAF2 Immunoblot

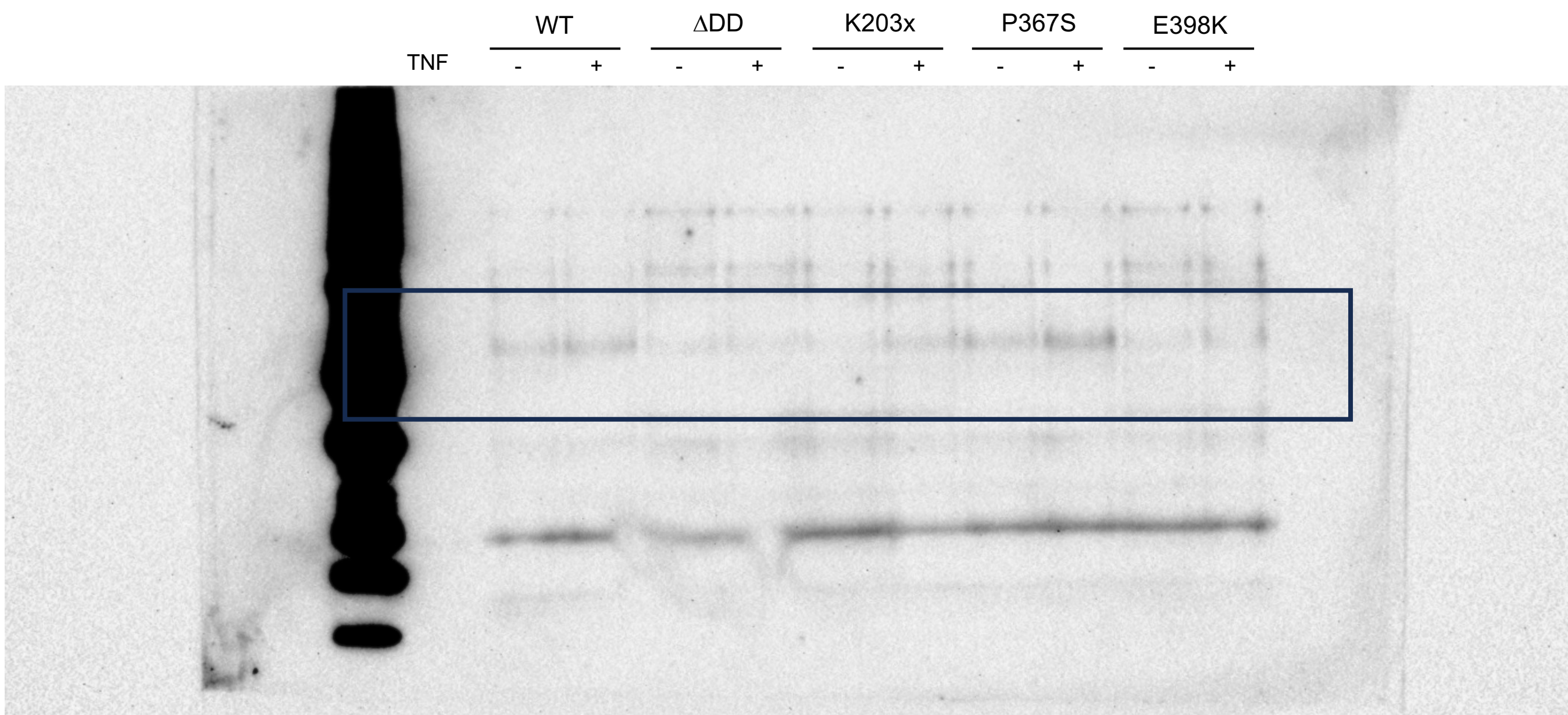

Full unedited Western Blots for Supplemental Figure 1
Whole Cell Lysate
anti-TRAF2 Immunoblot

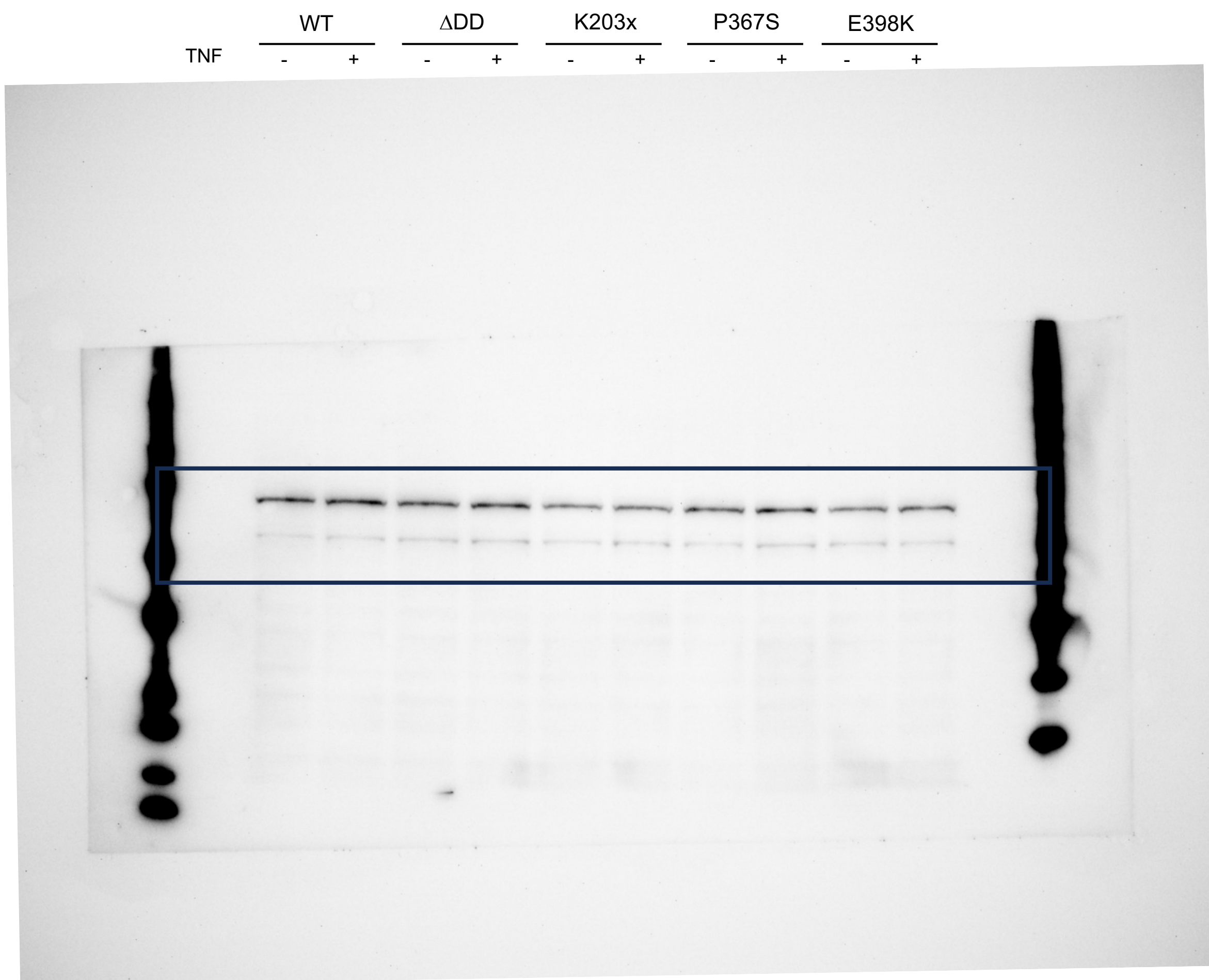

Full unedited Western Blots for Supplemental Figure 1
TNFR1 Immunoprecipitation
anti-RIP Immunoblot

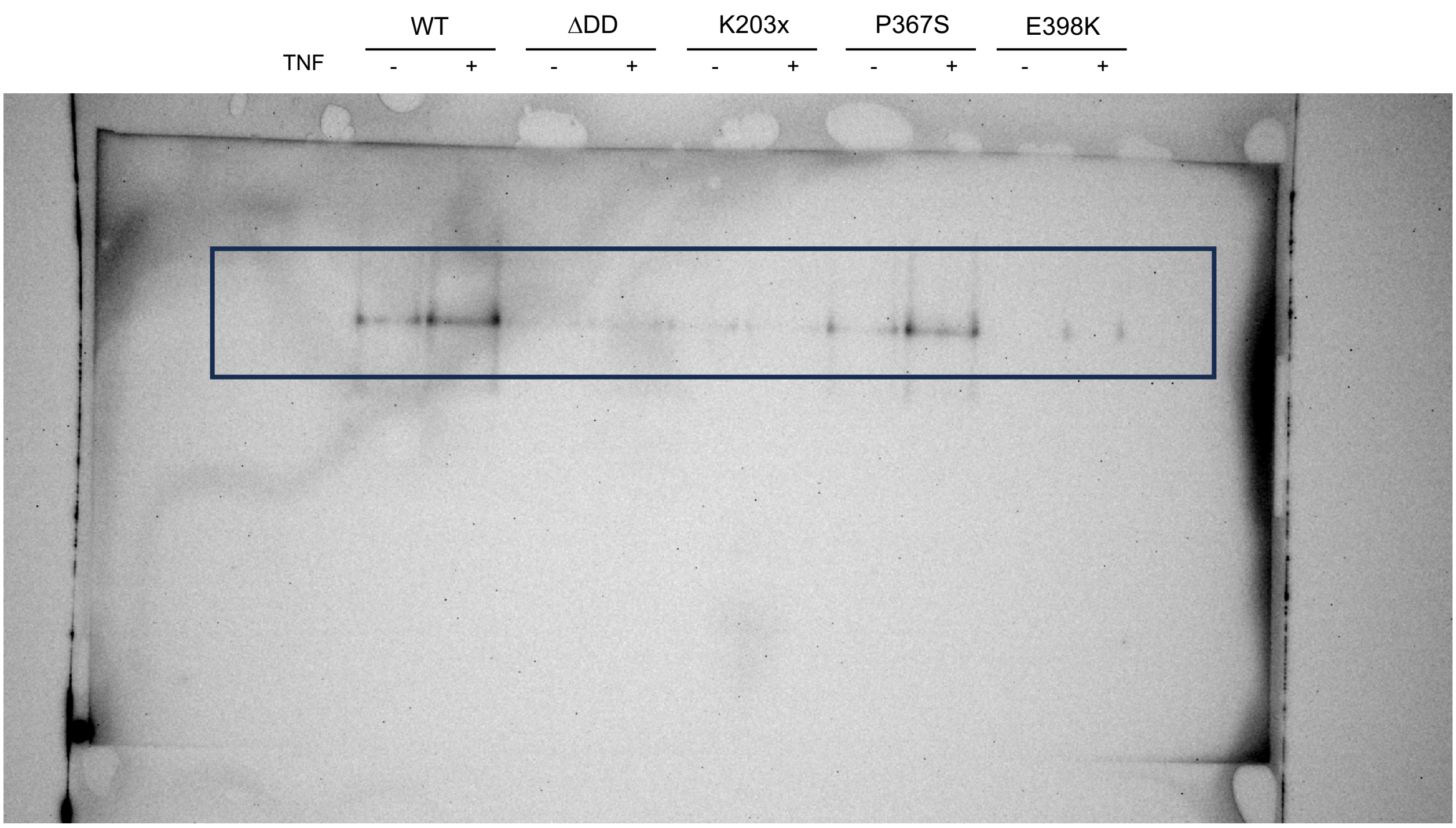

Full unedited Western Blots for Supplemental Figure 1
Whole Cell Lysate
anti-RIP Immunoblot

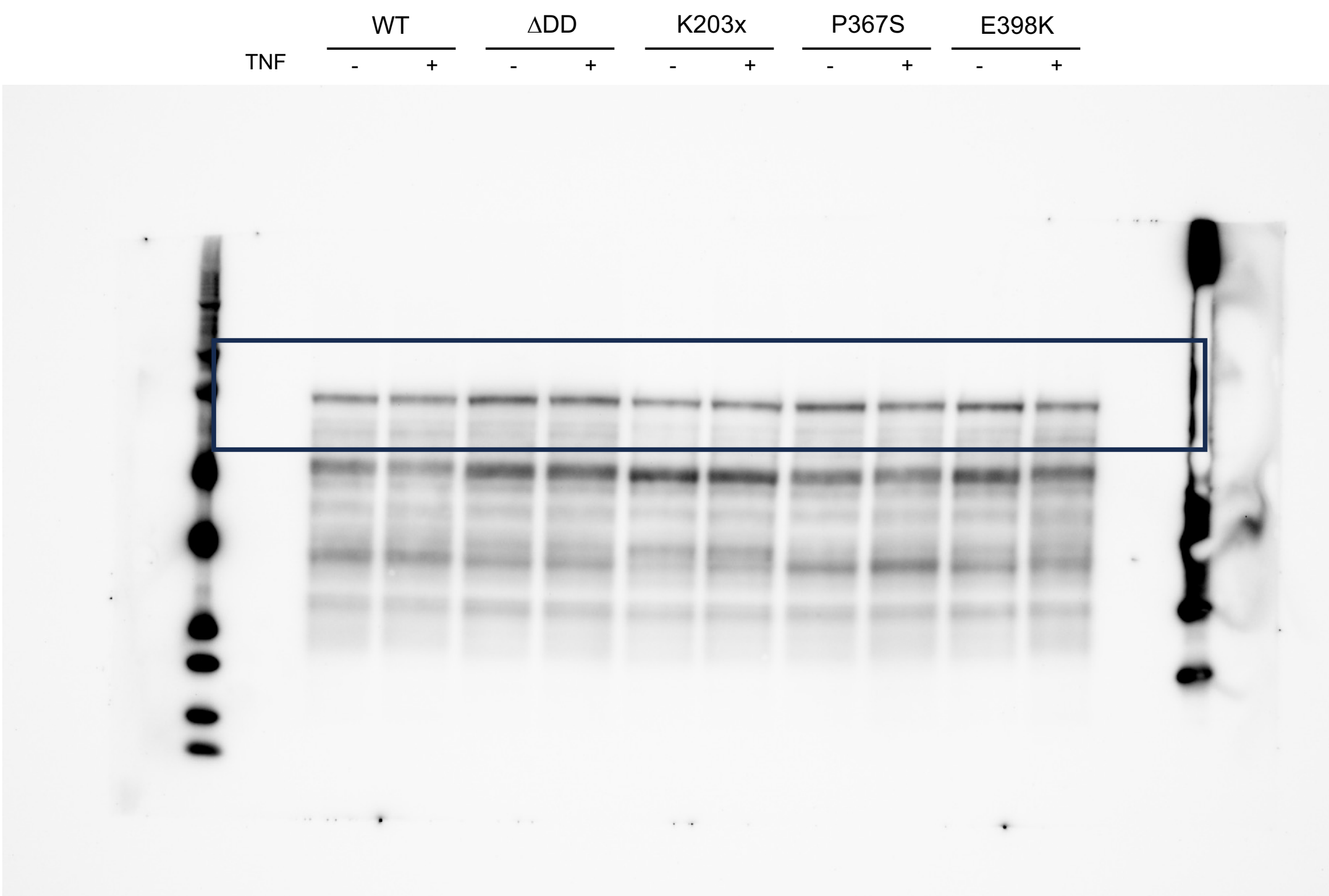

Full unedited Western Blots for Supplemental Figure 8B
Whole Cell Lysate
anti-β Actin Immunoblot

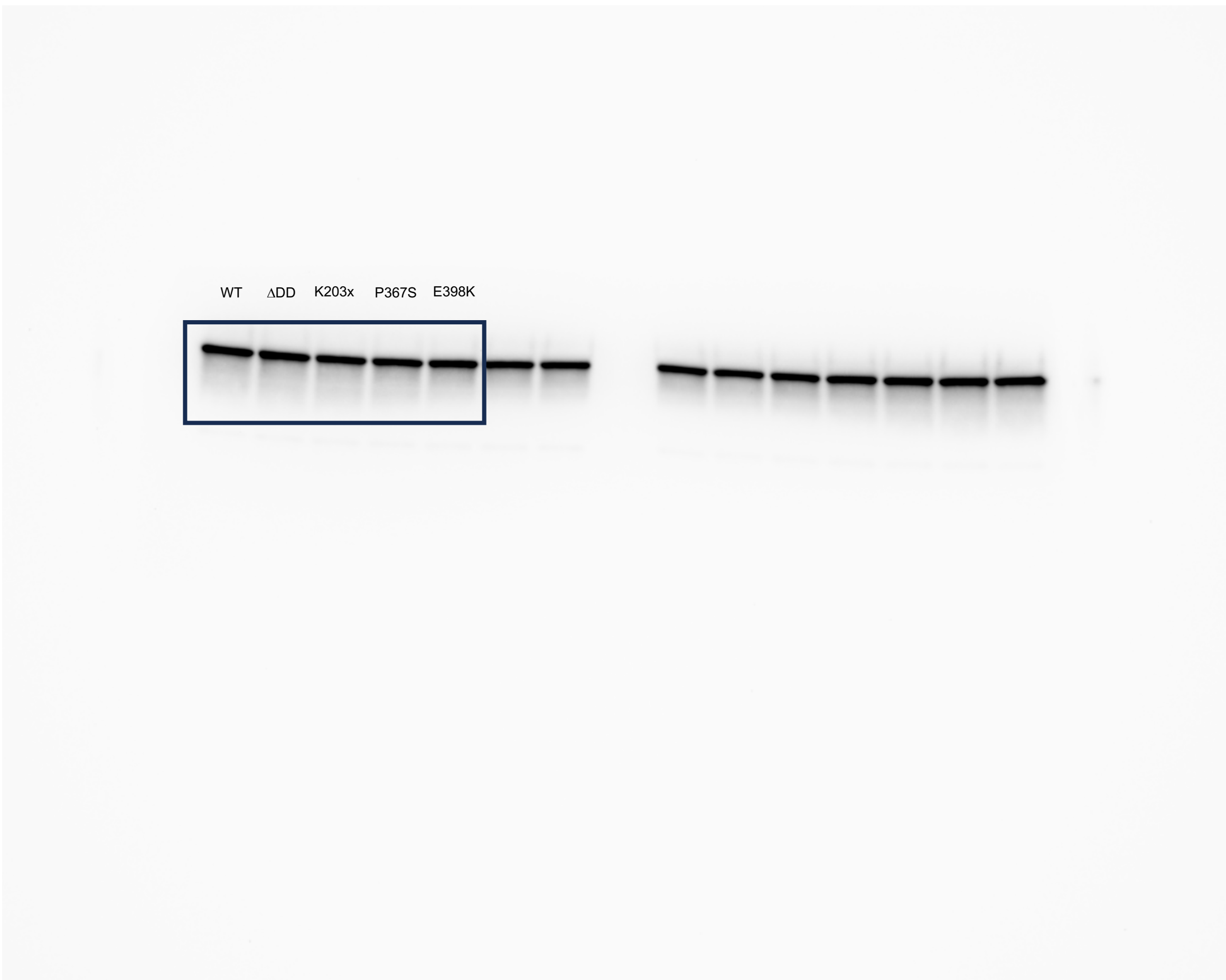

Full unedited Western Blots for Supplemental Figure 8B
Whole Cell Lysate
anti-LC3A/B No Bafilomycin Immunoblot

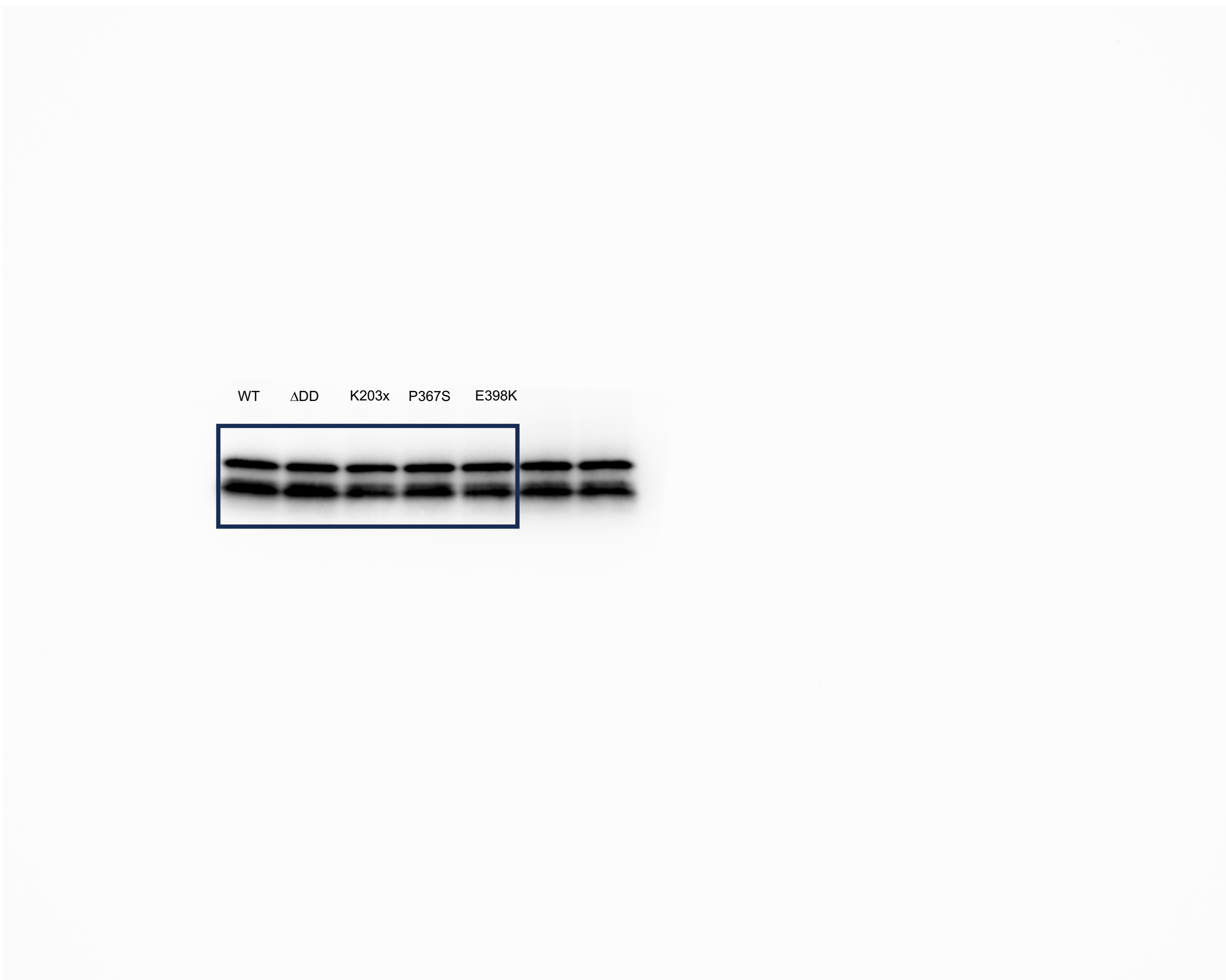

Full unedited Western Blots for Supplemental Figure 8B
Whole Cell Lysate
anti-LC3A/B With Bafilomycin Immunoblot

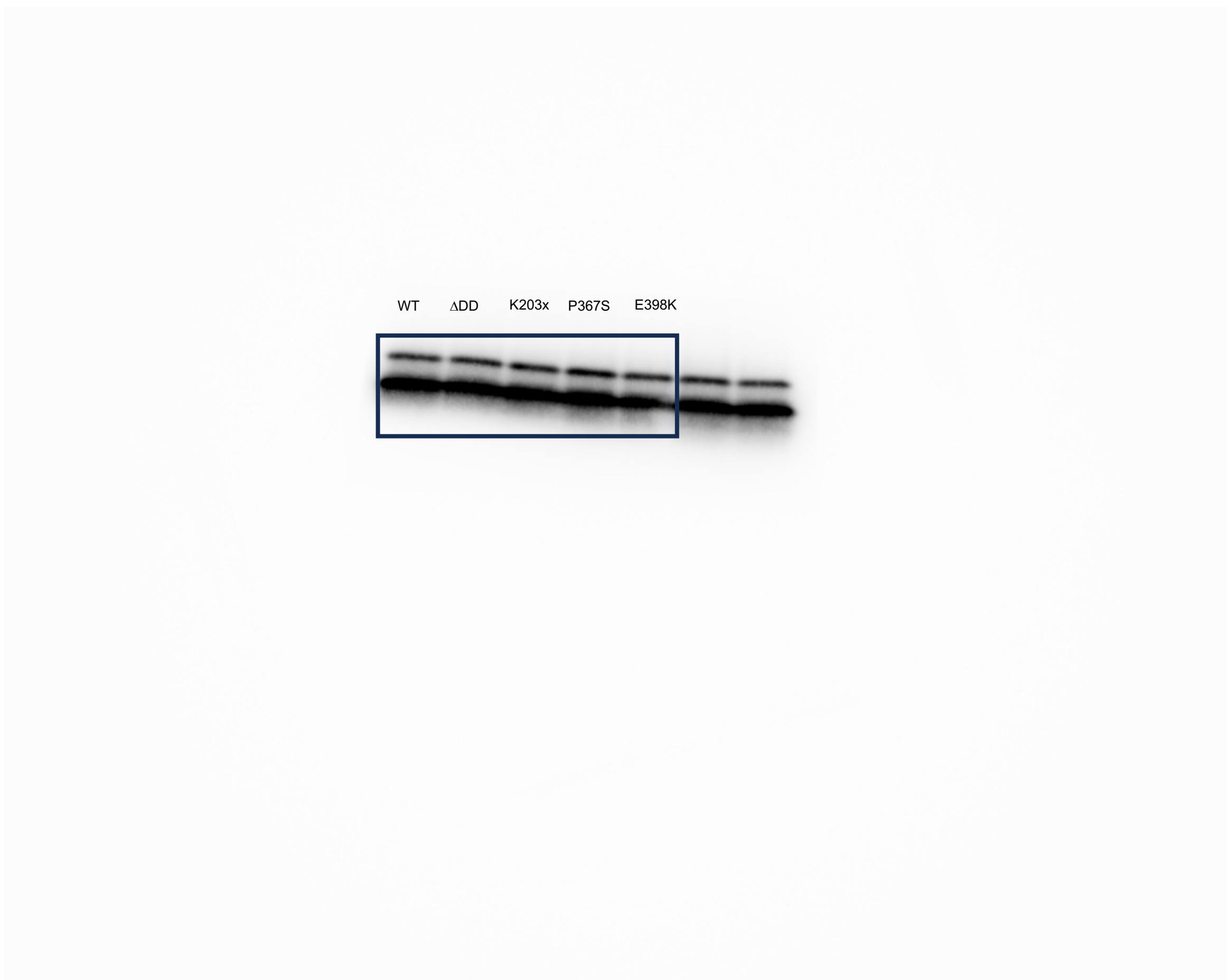

Full unedited Western Blots for Supplemental Figure 8D
Whole Cell Lysate
anti-β-Actin Immunoblot

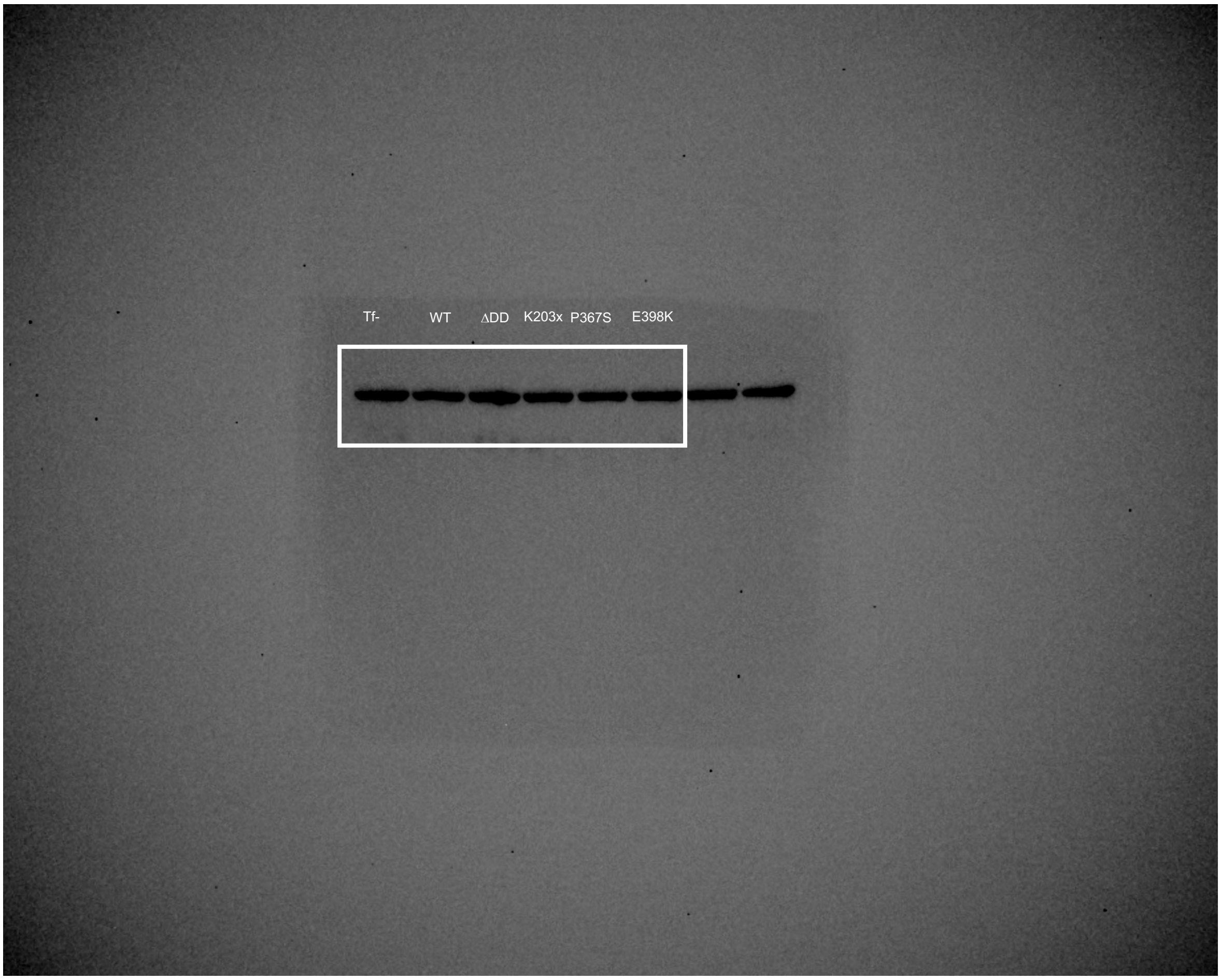

Full unedited Western Blots for Supplemental Figure 8D
Whole Cell Lysate
anti-ATF-4 Immunoblot

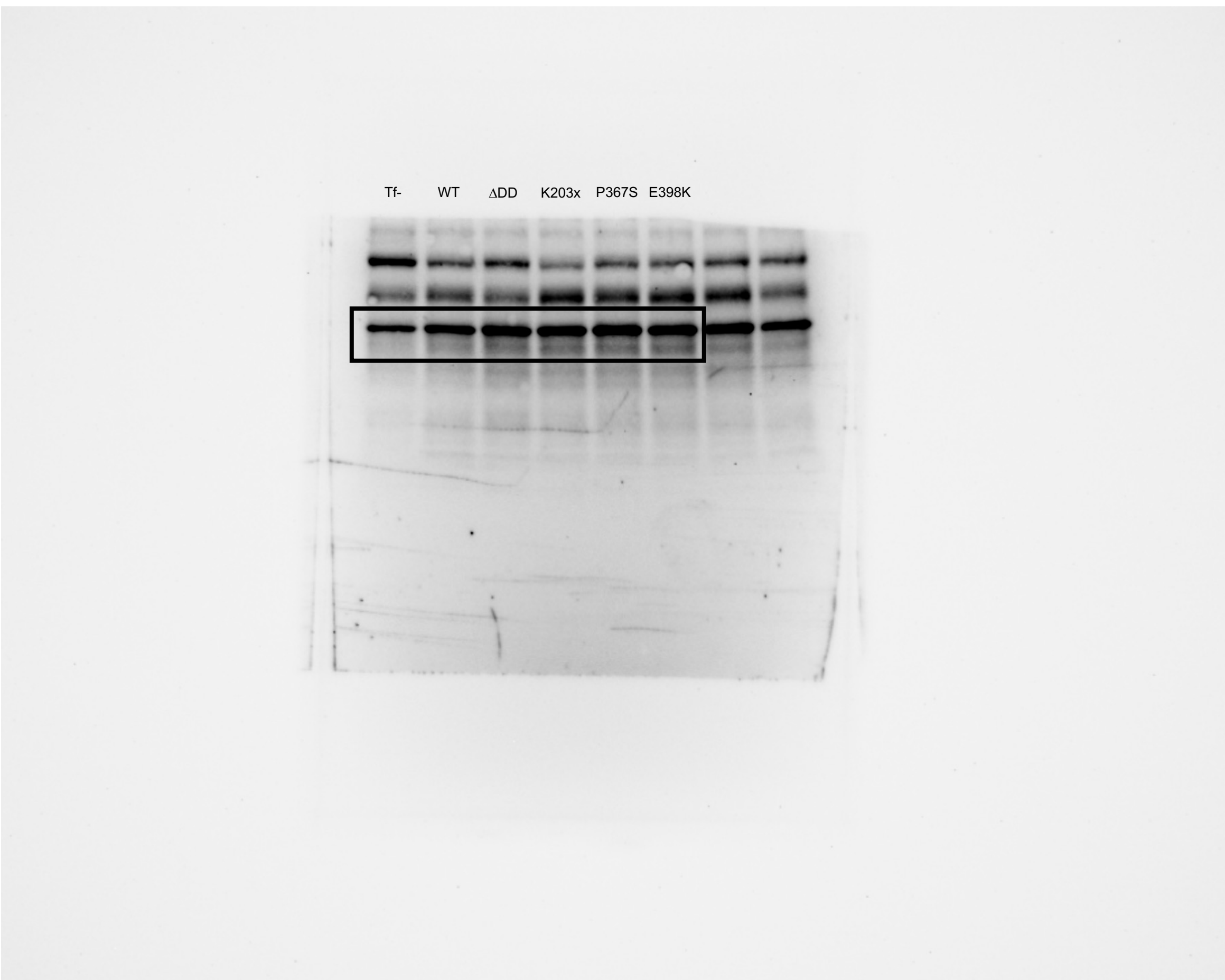

Full unedited Western Blots for Supplemental Figure 8D
Whole Cell Lysate
anti-phospho-P38 Immunoblot

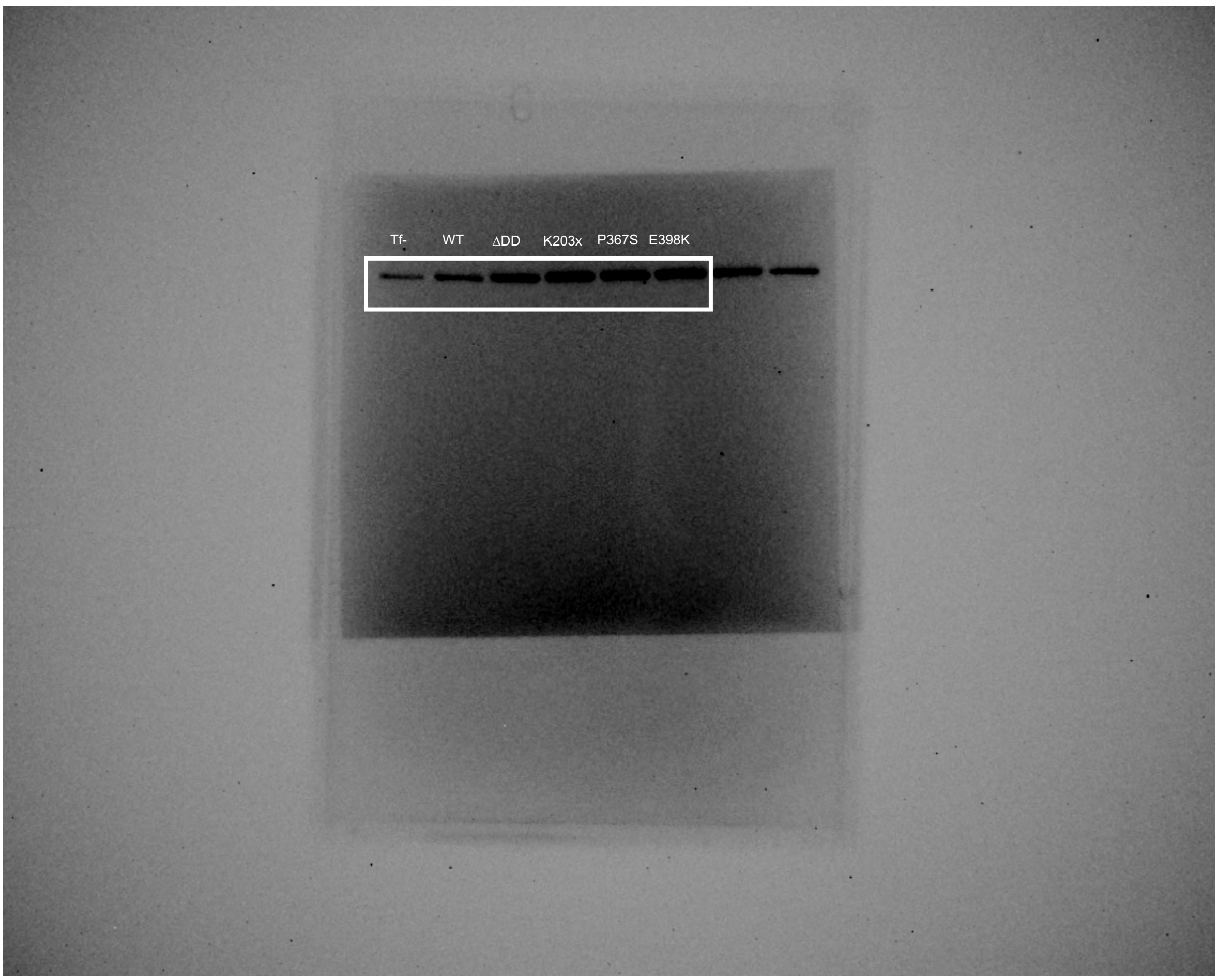

Full unedited Western Blots for Supplemental Figure 8D
Whole Cell Lysate
anti-Total-P38 Immunoblot

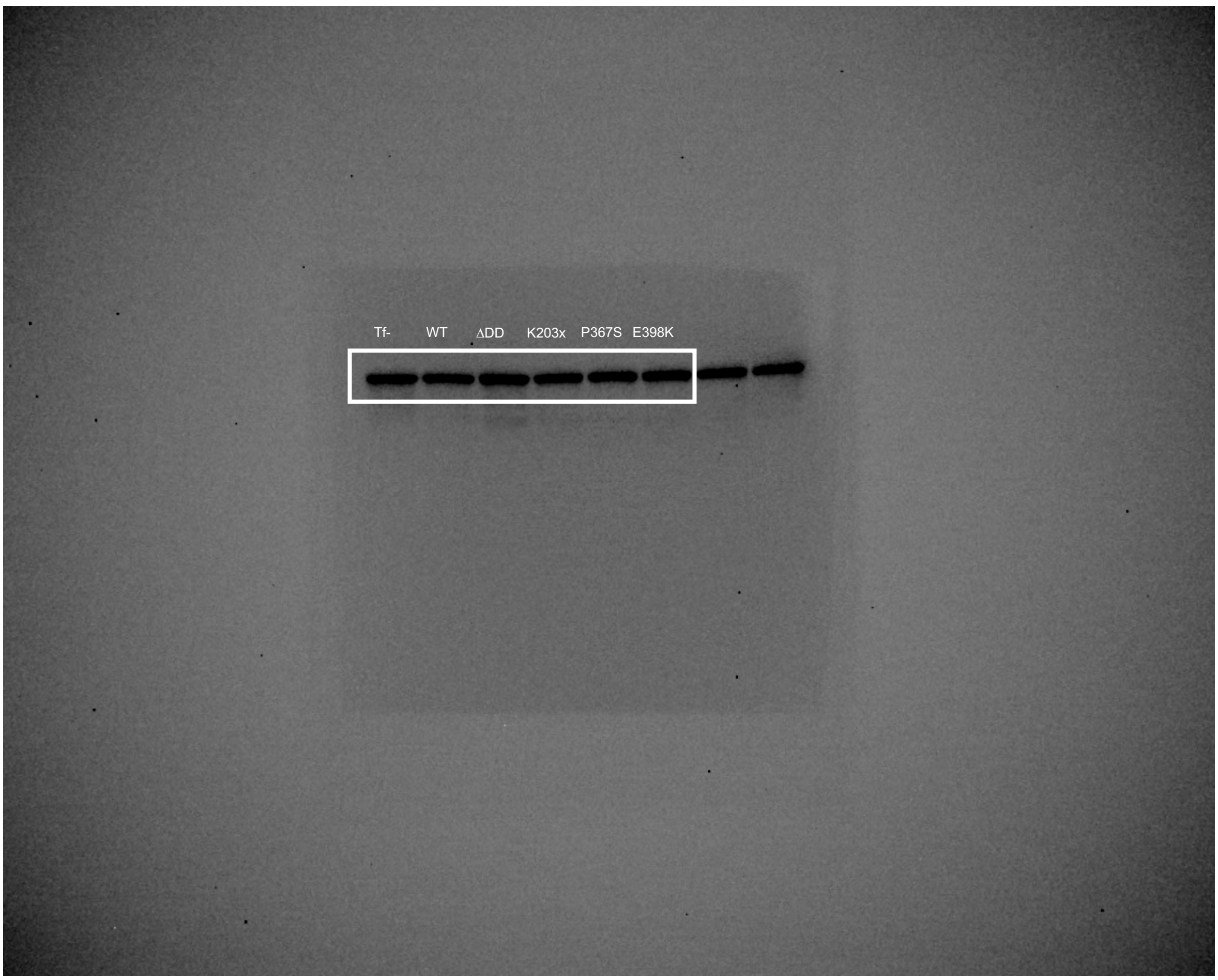

Supplement: Supplementary file 2 [file jci-136-196975-s198.pdf]
